# Sulfur-Centered Mechanism in Catalytic Methanolysis of Hydrosilanes Mediated by Air-Stable Mo_3_S_4_ Clusters

**DOI:** 10.1021/acs.inorgchem.4c05438

**Published:** 2025-03-21

**Authors:** Juanjo Mateu-Campos, María Gutiérrez-Blanco, Eva Guillamón, Vicent S. Safont, Jordi Benet-Buchholz, Mónica Oliva, Rosa Llusar

**Affiliations:** † Departament de Química Física i Analítica, Universitat Jaume I, Av. Sos Baynat s/n, Castelló de la Plana 12071, Spain; ‡ Institute of Chemical Research of Catalonia - ICIQ, Av. Països Catalans 16, Tarragona 43007, Spain

## Abstract

Methanolysis of hydrosilanes is catalyzed by incomplete cubane-type Mo_3_(μ_3_-S)­(μ-S)_3_ clusters functionalized with diamino and imidazolyl amino ligands under mild conditions. Silane activation mediated by the air-stable [Mo_3_(μ_3_-S)­(μ-S)_3_Cl_3_(ImNH_2_)_3_]Cl (ImNH_2_ = (1-methyl-1*H*-imidazol-2-yl)­methanamine) ([**3**]­Cl) cluster salt has been elucidated through a comprehensive experimental and theoretical study. Our results support the operation of a sulfur-centered mechanism without direct participation of the metals in clear contrast with all previously reported mechanisms catalyzed by transition metal complexes. The reaction proceeds in two steps, with the first one being the rate-determining step. The process starts with the hydrosilane Si–H bond activation, which occurs at one of the bridging sulfur atoms of the Mo_3_(μ_3_-S)­(μ-S)_3_ cluster unit. This step takes place through a concerted and asynchronous transition state with the participation of one methanol molecule to yield the silyl ether product and a bis­(hydrosulfido) intermediate. Analysis of this transition state reveals that its imaginary frequency is basically associated with the silane hydride transfer and the formation of the Si–O bond in agreement with the observed KIE results. The second step consists in the hydrogen release from the bis­(hydrosulfido) intermediate, from which the cluster catalyst is recovered. The same mechanism operates for the diamino [Mo_3_S_4_Cl_3_(en)_3_]Cl (en = ethylenediamine) ([**1**]­Cl) and [Mo_3_S_4_Cl_3_(dmen)_3_]^+^ (dmen = N,N′-dimethylethylenediamine) ([**2**]­Cl) cluster salts. The calculated free energy barriers for those cluster catalysts agree with the observed catalytic activities, giving further support to our mechanistic proposal.

## Graphical abstract

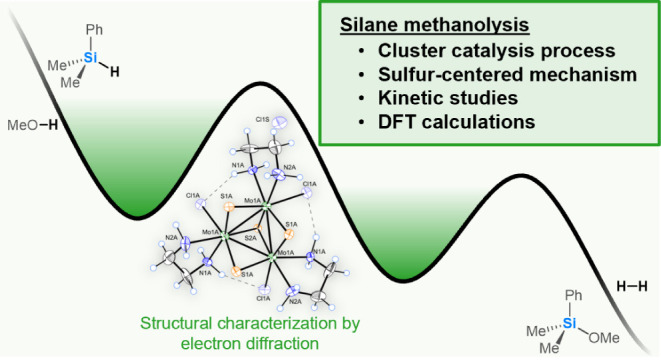

## Introduction

The cross dehydrogenative coupling (CDC) of hydrosilanes with alcohols is a useful reaction for synthesizing silyl ethers, which are important protecting groups in organic synthesis.^1^ Recently, there has been increasing attention on the hydrogen evolved during that process, enhancing the relevance of this system in the fields of hydrogen storage and production.^2^ Although this transformation is thermodynamically favorable, a catalyst is essential to overcome the slow kinetics of the reaction eq 1.^3^ To address this challenge, many efficient catalysts, both homogeneous and heterogeneous,^4,5^ have been reported for hydrosilane alcoholysis, which includes transition metal complexes of molybdenum,^6^ manganese,^7^ rhenium,^8^ iron,^9^ ruthenium,^10^ cobalt,^11^ iridium,^12^ nickel,^13^ platinum,^14^ copper^15^ or gold.^16^

1 $$R_{3}\mathrm{SiH}+{\mathrm{HOR}}^{\prime }\to R_{3}{\mathrm{SiOR}}^{\prime }+H_{2}$$

Different catalytic mechanisms have been proposed, common pathways include the nucleophilic attack of alcohol on η^1^-M–H–(SiR_3_) or η^2^-M–(H–SiR_3_) complexes as illustrated in Figure 1.^8,10,12^ Luo and Crabtree introduced the first mechanism proposal based on the formation of these σ-complexes, which facilitate the formation of M-H species upon nucleophilic attack.^17^ Recent investigation shows that hydrogen tunneling can also play a key role in these reaction mechanisms.^18^ Alternatively, hydrosilane activation may occur by oxidative addition of the silane to the metal center, rather than through metal-silane interactions.^7,9,11^ In contrast, copper and gold catalysis involve metal hydride and metal alkoxide intermediates in which silane activation proceeds by a σ-bond metathesis mechanism.^15,16^ Silane activation through the synergetic play of a metal and a ligand has also been reported.^19^ In particular, metal–sulfur complexes allow for cooperative Si–H bond activation. Oestreich and coworker reported an in-depth experimental and theoretical investigation on the reaction mechanism mediated by ruthenium­(I) thiolate complexes.^20^ According to their studies, the mechanism involves the heterolytic splitting of the Si–H bond across the polar Ru–S bond and occurs without changes in the metal oxidation state to generate a ruthenium hydride and a silylthiolate moiety.

**Figure 1:**
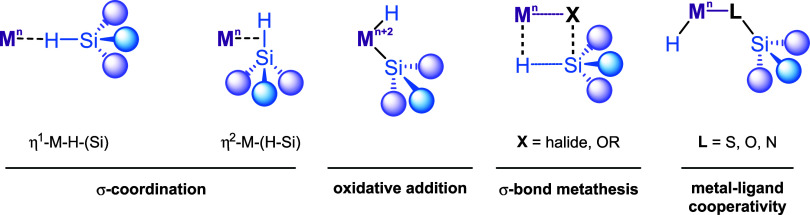
Possible activation modes for hydrosilanes in metal complexes.

The overall Si–H bond activation event in all mechanisms in Figure 1 results in the generation of metal hydrides. In contrast, our work on the hydrosilylation of nitro compounds to afford aniline using incomplete cubane type Mo_3_S_4_ clusters does not provide any evidence on the formation of Mo–H species.^21^ On the other hand, we have shown that H_2_ activation catalyzed by Mo_3_S_4_ complexes takes place through a sulfur-centered mechanism which involves two of the bridging sulfur ligands of the cluster unit.^22–24^ In simple terms, a silicon atom can be considered a ‘bulkier’ hydrogen atom. Although this is certainly an oversimplification because the Si–H bond in hydrosilanes is weaker than the H–H bond in molecular hydrogen, quantified by a difference in the bond energy of 18 kcal·mol^–1^.^25^ Thus, the heterolytic cleavage of hydrosilanes is energetically more feasible. Given the ability of the Mo_3_S_4_ system to activate dihydrogen with the concomitant formation of hydrogenosulfido Mo_3_(μ_3_-S)­(μ-S)­(μ-SH)_2_ intermediates, its potential to activate Si–H bond deserves some attention. In this way, hydrosilane activation would occur without direct participation of the metal and therefore without the generation of metal hydrides. For this purpose, the methanolysis of dimethylphenylsilane was chosen as a model reaction and three different Mo_3_S_4_ cluster catalysts were included in our study.

Herein, we report the results of our mechanistic study on the methanolysis of dimethylphenylsilane promoted by Mo_3_S_4_ cluster catalysts. The synthesis and crystal structure of one of these catalysts with formula [Mo_3_S_4_Cl_3_(en)_3_]Cl is also presented. By using NMR and ESI-MS techniques in combination with properly planned control experiments, we were able to demonstrate that hydrosilane Si–H bond activation occurs with a direct participation of the bridging sulfur atoms. Supported by kinetic studies and DFT calculations, we provide compelling evidence of a sulfur-centered reaction mechanism. This cluster catalysis process takes place through a concerted transition state, enabling simultaneous activation of both, the silane and methanol molecules.

## Results and Discussion

From previous studies, we know that the trinuclear diamino [Mo_3_S_4_Cl_3_(dmen)_3_]^+^ cluster cation is an efficient catalyst for the hydrosilylation of nitro compounds to form anilines.^21^ Due to the complexity of this reaction mechanism, we decided to select a simpler reaction, such as the methanolysis of hydrosilanes, to gain knowledge into the Si–H bond activation mediated by these trinuclear complexes. For that purpose, we have chosen two diamino and one imidazolyl amino cluster cations of formula [Mo_3_S_4_Cl_3_(en)_3_]^+^ (en = ethylenediamine) (**1** ^+^), [Mo_3_S_4_Cl_3_(dmen)_3_]^+^ (dmen = N,N′-dimethylethylenediamine) (**2** ^+^) and [Mo_3_S_4_Cl_3_(ImNH_2_)_3_]^+^ (ImNH_2_ = (1-methyl-1*H*-imidazol-2-yl)­methanamine) (**3** ^+^). These last two compounds are excellent catalysts for the direct hydrogenation of alkynes, nitro and azo compounds. Hydrogen activation in these cases occur at the bridging sulfur sites of the Mo_3_(μ_3_-S)­(μ-S)_3_ unit.

### Synthesis and Characterization of the Catalysts

The new trinuclear cluster cation **1** ^**+**^ has been prepared by partial substitution of the outer ligands of the Mo_3_S_4_Cl_4_(PPh_3_)_3_(H_2_O)_2_ cluster precursor. This synthetic procedure developed in our group and represented in Scheme 1 has been successfully employed for the synthesis of other Mo_3_S_4_ clusters decorated with diphosphino, aminophosphino, imidazolyl amino and other diamino cluster compounds.^26^

**Scheme 1:**
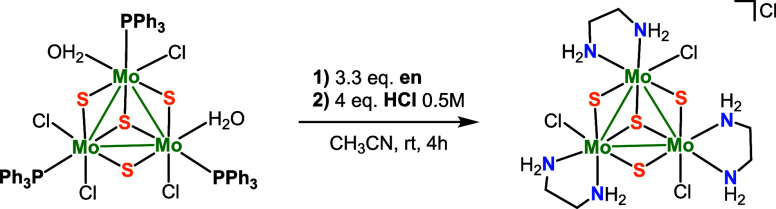
Synthetic Route for the Preparation of Cluster [Mo_3_S_4_Cl_3_(en)_3_]­Cl

The [**1**]Cl cluster salt was isolated in 70% yield as an air-stable green solid. Nanometric needles of [**1**]­Cl were crystallized by slow evaporation from an acetonitrile solution at room temperature. All attempts to obtain larger single crystals failed so that conventional single crystal X-ray diffraction techniques could not be used for structure determination. Single-crystal electron diffraction techniques (3D-ED) were recently demonstrated to provide diffraction data of sufficiently high quality from crystals whose size is in the range of those found in powder samples. Advanced instruments combining both electron microscopy and diffraction techniques (Synergy-ED Rigaku-JEOL) now allow for selecting and recording diffraction data from nanosized crystallites and resolving their structure with similar accuracy to single crystal X-ray diffraction.^27,28^ Consequently, 3D-ED data were collected at 100 K directly from the synthesized catalyst powder crystallized by slow evaporation from acetonitrile sample solutions in the presence of NH_4_PF_6_. For the sample preparation four 10 μL drops of the acetonitrile suspension were deposited on a copper-graphite grid and carefully evaporated until only residual amounts of acetonitrile were present (for more details see Section S3). The sample was then cryo-prepared at 215 K and after sublimating the residual acetonitrile and water amounts, measured at 100 K under vacuum conditions. Crystallite selection was performed by TEM imaging (see Figure 2 for a picture of crystallite 5).

**Figure 2:**
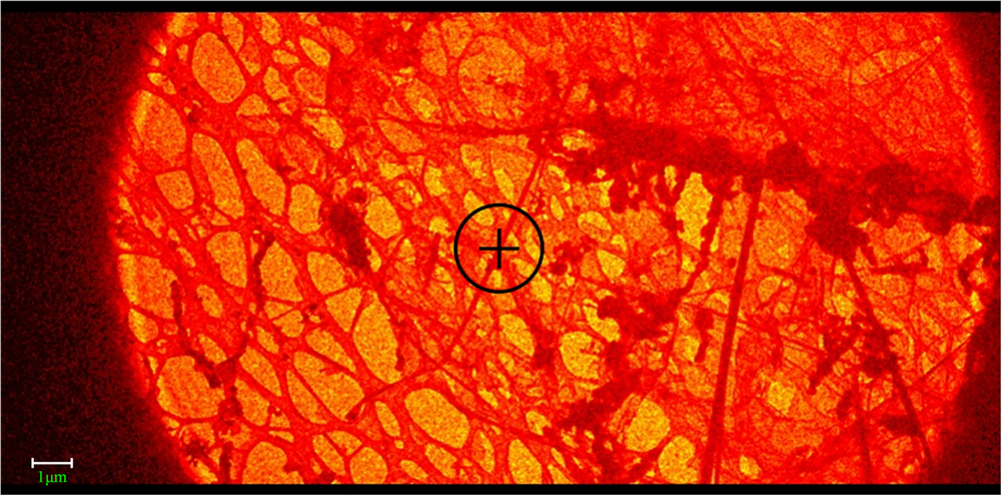
TEM image showing in the central sphere the crystal needle 5 used for unit cell indexation and structure solution of the cluster [**1**]­Cl. The diameter of the sphere is of 2 μm.

Data collection from 11 selected crystallites was performed. From them, six could be indexed in the trigonal space group *P*3*c*1. Crystal number 5 was used for structure solution and refinement was performed based on the merged data of crystals 5, 2, and 3. Final data refined to a R1 value of 13.70% with a completeness of 100% and data resolution of d: 0.7 Å. The [**1**]­Cl salt crystallizes with two independent cluster cations per asymmetric unit, two chloride atoms and acetonitrile and water solvent molecules. Figure 3 shows ORTEP representation of one of the **1** ^+^ cations together with the most relevant bond distances. The structure contains an incomplete cubane-type cluster in which the molybdenum and sulfur atoms occupy adjacent vertices with a missing metal atom. The capping sulfur atoms lie on a *C*_3_ symmetry axis. Each molybdenum atom presents a pseudo-octahedral coordination environment with three sulfur ligands, one chlorine, and two nitrogen atoms from the diamine ligand. In general, the metal–metal and metal–sulfur distances within the Mo_3_S_4_ cluster follow the tendencies observed for other trinuclear Mo_3_S_4_ species.^29^

**Figure 3:**
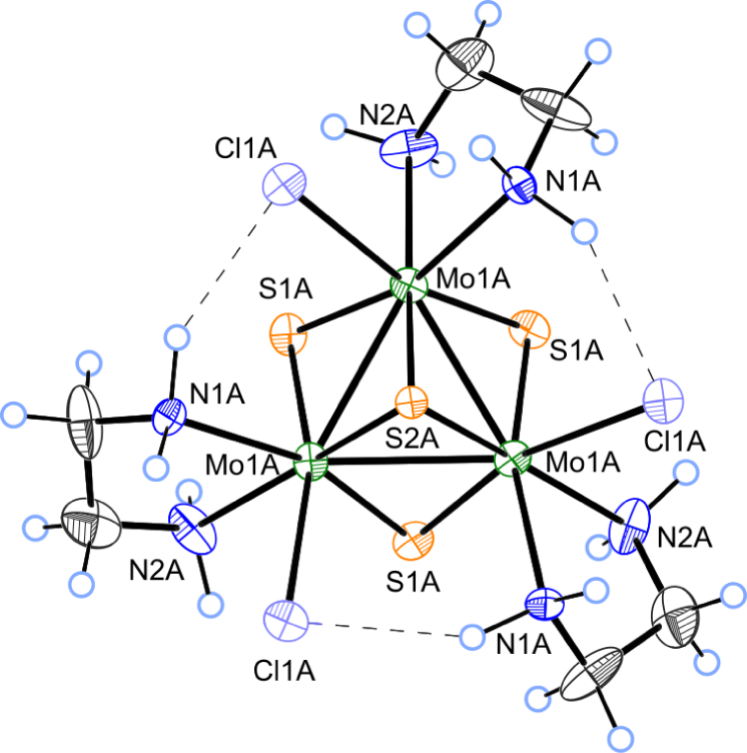
ORTEP-Drawing (thermal ellipsoids draw at 50% level) showing the structure of the **1** ^**+**^ cluster. Main bond distances: Mo–Mo = 2.711(5) Å, Mo–S2A = 2.338(7) Å, Mo–S1A_(trans‑Cl)_ = 2.260(7) Å and $\mathrm{Mo}-S1A_{\left({\mathrm{cis‐NH}}_{2}\right)}=2.271(7)\;Å$ . CCDC reference number 2406527.

The three metal atoms define an equilateral triangle with Mo–Mo bond distances of 2.711(5) and 2.726(5) Å – one per crystallographically independent cluster unit – which are consistent with a single metal–metal bond. As observed for other Mo_3_S_4_ clusters derivatized with bidentate ligands, only one of all possible isomers is formed, where the diamine ligand is coordinated asymmetrically to the metal cluster core with one nitrogen atom coordinated trans and the other cis to the capping sulfur atom (μ_3_-S).^30^ This specific arrangement leads to a *C*_3_ symmetry cluster endowed with intrinsic backbone chirality. The short Cl···H­(−N) distances in the cluster compound (2.43–2.93 Å) between the chlorine atom and the hydrogen atom of the amino groups of the adjacent metal center evidence the presence of hydrogen bonding interactions, as previously observed for other amino-containing Mo_3_S_4_ clusters.^21,29^

The structural integrity of the cluster in solution was proved by multinuclear NMR spectroscopy and electrospray ionization mass spectrometry (ESI-MS) (see Figures S1–S5). Consistent with the *C*_3_ symmetry of the complex, the ^13^C NMR spectrum reveals two singlets corresponding to the ethylene bridged groups of the diamine. Nonetheless, ^1^H NMR displays six distinct signals registered as three multiplets and three broad signals. Multidimensional ^1^H–^13^C gradient HSQC and COSY correlation experiments were required for the complete signal assignment. The former technique was useful to discriminate between the diastereotopic protons of the amino moieties (NH) and the protons of the ethylene bridged groups. Note that one of the diastereotopic protons of *C*_2_ (see Figure S3) overlaps with one proton of the amino moiety (H_D_). Finally, COSY experiments were employed to identify the proton signals of the nitrogen atom (H_A_ and H_B_), which are next to the carbon containing diastereotopic protons (*C*_2_). The positive ESI-MS spectrum shows a peak centered at *m*/*z* = 702.7170 assigned to the **1** ^+^ cation on the basis of the *m*/*z* value and its isotopic pattern (see Figure S5). Other peaks appear at *m*/*z* = 666.7409 [M–HCl]^+^, *m*/*z* = 630.7645 [*M*-(HCl)_2_]^+^ and *m*/*z* = 592.7880 [M–(HCl)_3_]^+^ due to successive losses of HCl molecules at the applied voltage. This effect has been previously observed for the aminophosphino [Mo_3_S_4_Cl_3_(edpp)_3_]^+^ (edpp = (2-aminoethyl)­diphenylphosphine) cluster during collision induced decomposition (CID) experiments.^31^

### Catalytic Performance

In this work, we chose the cross dehydrogenative coupling of methanol and dimethylphenylsilane as probe reaction to unveil the Si–H activation mechanism mediated by incomplete cubane-type Mo_3_(μ_3_-S)­(μ-S)_3_ clusters. First, we tested the catalytic activity of three Mo_3_S_4_ complexes (see Figure 4), **1** ^+^and **2** ^+^ differ in the nature of the amino group (primary and secondary, respectively) while in **3** ^+^, one of the amino groups in **1** ^+^ has been replaced by an imidazolyl moiety. Catalytic experiments were conducted under typical reaction conditions employed for catalytic protocols using precious metal (30 °C and 1 mol % of catalyst) in a Schlenck flask. Although all three catalysts proved to be active, the lowest performance was observed for the diamino cluster complex **2** ^**+**^. That is methylation of the amino group decreases the catalytic activity. The most active catalyst is the imidazolyl amino complex **3** ^**+**^; therefore replacement of one of the −NH_2_ groups in **1** ^+^ by the imidazolyl group enhances its performance.

**Figure 4:**
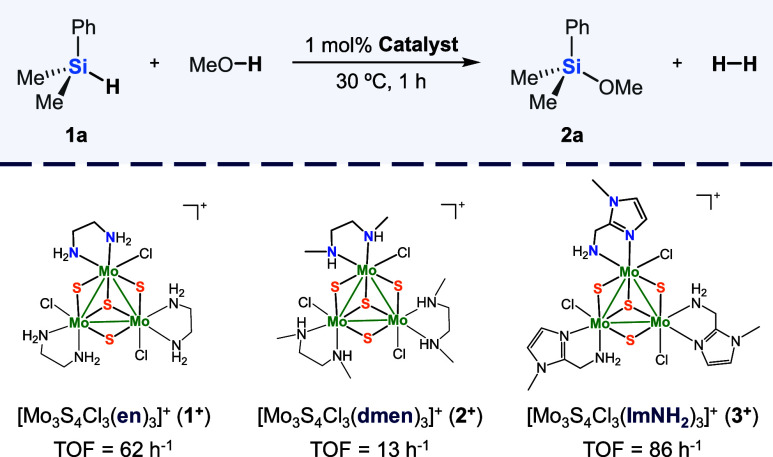
Amino-containing Mo_3_S_4_ cluster catalysts screening for the methanolysis of dimethylphenylsilane. Reaction conditions: silane **1a** (0.5 mmol), MeOH (2 mL), 1 mol % catalyst, 30 °C, 1 h. TOF values are calculated from the silane conversion after 1 h of reaction. Silane conversion was determined by GC using anisole as internal standard. Experiments were performed at least twice.

Silane evolution was assessed using complex **3** ^**+**^ as catalyst by monitoring the reaction mixture by gas chromatography. Full conversion was achieved after 120 min as seen in Figure 5a. During the chemical reaction the expected methoxosilane (**2a**) coexists with residual amounts (7%) of the disiloxane (**2b**) sideproduct. The presence of traces of water in the alcohol can generate silanol species which react to generate the disiloxane side product. Hydrogen evolution was also assessed under the same catalytic conditions using an inverted buret setup (open system) or pressure transducer (closed system) implemented in the Man on the Moon X104 kit (see Section S4 for further details). Both reaction profiles, depicted in Figure 5a,b, are in full agreement.

**Figure 5:**
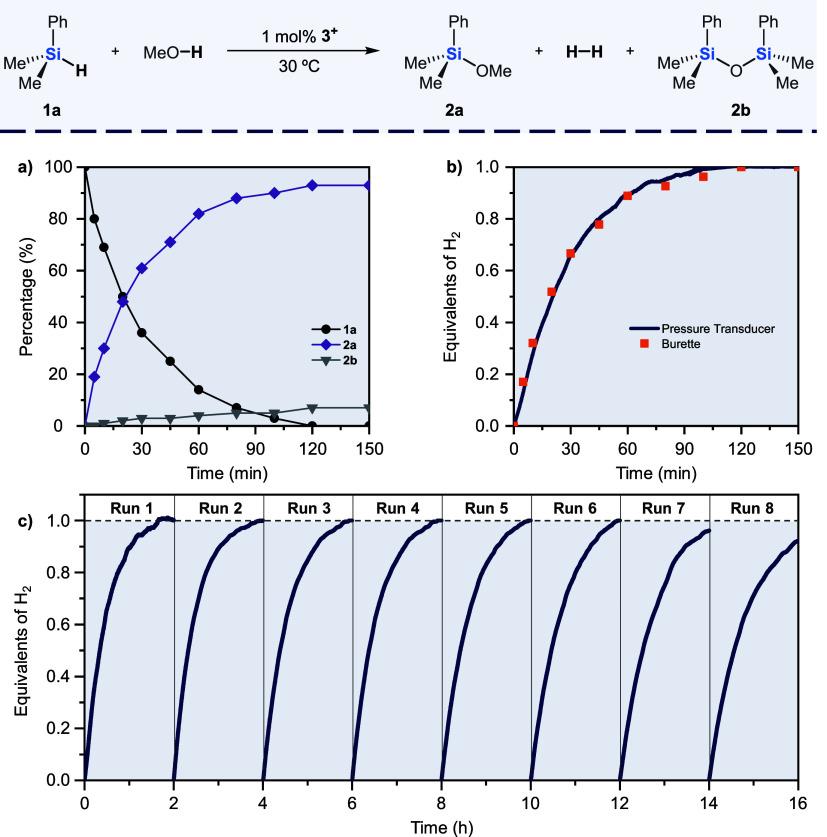
Concentration/time diagram for silane evolution using cluster **3** ^**+**^ (a). Comparative hydrogen evolution monitoring using an inverted buret setup and a pressure transducer device (Man on the Moon X104 kit) (b). Kinetic profile (obtained using the pressure transducer device) for the recycling experiments by successive additions of silane to a methanolic solution of the cluster catalyst (c). Reaction conditions: silane **1a** (0.5 mmol), MeOH (2 mL), 1 mol % **3** ^**+**^, 30 °C.

Next, the cluster evolution along the reaction course was investigated. Initially, the molecular peak at *m*/*z* = 856 assigned to the **3** ^+^ cluster catalysts coexists with a minor peak centered at *m*/*z* = 820 attributed to one HCl loss.^29^ Reaction monitoring for 120 min reveals that catalysis occurs without degradation or modification of the cluster catalysts (see Figure S10). Hypothetical hydrido and/or silyl-molybdenum cluster intermediates are not detected, as occurred during the reduction of nitrobenzene to aniline with hydrosilanes catalyzed by the **2** ^+^ cluster.^21^ Notice that monitoring of the diphosphino Mo_3_S_4_ cluster by ESI-MS during the catalytic transfer hydrogenation of nitrobenzene with formic acid allowed the detection of formate and hydride cluster intermediates.^32^ Therefore, silane activation presumably occurs without Mo–Cl bond cleavage.

It is established that ESI-MS techniques fail sometimes to detect certain reaction intermediates.^33^ For that reason, we decided to monitor by NMR spectroscopy the reaction of the **3** ^+^ cluster in the presence of 1 to 50 equiv of dimethylphenylsilane (**1a**) in DMSO-*d*_6_. The ^1^H NMR spectra registered (Figure S11) do not show any signal due to hydride formation. Characteristic hydride signals for diphosphino Mo_3_S_4_ cluster hydrides appear between −3.0 and −2.0 ppm.^34^ However, hydride formation can not be completely ruled out because we have previously observed that the hydride signal in the aminophosphino W_3_S_4_ hydride overlaps with the proton signals of the NH_2_ group (4.5–1.0 ppm) due to a fast exchange causing an increase in their intensity.^35^ However, in our case amino proton signals integrate as two for NH_2_ group so formation of Mo–H species can be ruled out. We can also conclude that there is not partial decoordination of the imidazolyl amino ligand that allows the formation of metal-silane σ-complexes, since no loss of the cluster catalyst *C*_3_ symmetry is observed during the ^1^H NMR reaction monitoring.

Once the cluster integrity during the catalytic process was demonstrated and therefore the operation of a cluster catalysis mechanism was established, we proceeded to evaluate the reusability of Mo_3_S_4_ cluster in the methanolysis of dimethylphenylsilane. This experiment was carried out at 30 °C by successive additions of silane **1a** and the hydrogen evolution was recorded for two hours.^24^ As shown in Figure 5c, the cluster complex **3** ^**+**^ was used during six runs without a significant loss in the catalytic performance. Then, the protocol was applied up to eight times, although longer reaction times were needed. Analysis of the resulting reaction mixture by ESI-MS and ^1^H NMR (see Figure S12) confirmed the integrity of the cluster unit after the eighth cycle. Thus, we attribute the decrease in catalytic activity to a change in the solvent composition due to an increase of the silyl ether (**2a**) concentration after successive additions of silane.

### Mechanistic Insights

At this point, we consider the possibility of a ligand assisted mechanism with participation of the cluster sulfur atoms. As stated in the introduction, hydrogen activation in the hydrogenation of azobenzene, diphenylacetylene or sulfoxides catalyzed by Mo_3_S_4_ clusters takes place through sulfur-centered mechanisms.^22–24^ To obtain experimental evidence in this regard, we blocked two of the bridging sulfurs by the addition of dimethyl acetylenedicarboxylate (dmad) during the catalytic protocol, which resulted in the null conversion of the silane. It is well-known that Mo_3_S_4_ clusters react with symmetrical alkynes to form dithiolene [Mo_3_(μ_3_-S)­(μ-S)­(μ-SC­(CO_2_CH_3_)=C­(CO_2_CH_3_)­S)­Cl_3_(ImNH_2_)_3_]^+^ adducts in which only one of the bridging sulfur atom remains present.^36,37^ In our case, the formation of the dithiolene adduct was confirmed by ESI-MS with the appearance of a major signal centered at *m*/*z* = 998 (see Figure S13).

Next, different tests were carried out to gain evidence of the origin of the hydrogen evolved in the presence of PhMe_2_SiH/CH_3_OH, PhMe_2_SiH/CD_3_OD and PhMe_2_SiD/CH_3_OH mixtures. Reactions were monitored by ^1^H NMR in DMSO-*d*_*6*_ or CD_3_OD using a Young’s tube (see Section S7 for further details). As expected, the use of PhMe_2_SiH and CH_3_OH yields the formation of H_2_, detected as a singlet at 4.61 ppm (Figure S14a). When the PhMe_2_SiH/CD_3_OD mixture is employed, HD is formed as the major product detected as a triplet centered at 4.52 ppm (see Figure S14b). In this case, H_2_ is also formed (HD/H_2_ *ca*. 7/3), very likely due to the presence of trace amounts of H_2_O in the deuterated methanol.^18^ The reaction between PhMe_2_SiD and CH_3_OH also produces a mixture of HD/H_2_ in *ca*. 8/2 ratio (see Figure S14c).

Finally, we investigated the reaction kinetics of the methanolysis of dimethylphenylsilane (**1a**) with respect the silane (notice that methanol, the other reactant, is the solvent of the reaction) at 30 °C (see Figure S15). No induction period is observed, which indicates that cluster **3** ^+^ is the catalyst in this process instead of a precatalyts. The time course of the reaction was monitored by measuring the H_2_ evolution using the pressure transducer implemented in the Man on the Moon X104 kit. The ln­[PhMe_2_SiH] *vs*. time linear dependence is indicative of a first-order kinetics and leads to a rate constant *k*_1_ = 5.78 × 10^–4^ s^–1^. This kinetic result suggests that the silane participates in the rate determining step or, in a previous one. In addition, we used the same methodology to evaluate the kinetic isotope effect (KIE). Kinetic results evidence a negligible effect on the reaction rate when deuterated methanol is used ( ${\mathrm{KIE}}_{{\mathrm{CH}}_{3}OH/{\mathrm{CD}}_{3}OD}=1.04$ ); however, a noticeable KIE is observed using PhMe_2_SiD (KIE_Si**H**/Si**D**_ = 1.93). These results pinpoint the Si–H bond activation as the rate determining step, which is consistent with a first-order reaction on the silane concentration (see Figure S15).

### Computational Studies

Our experimental results clearly indicate a direct participation of the sulfur atoms in the reaction mechanism and suggest that no metal-hydride species are generated during the catalytic cycle. Also, decoordination of the outer ligands and therefore generation of vacant coordination sites which could favor the formation of a η^1^-M–H–(SiR_3_) or η^2^-M–H–(SiR_3_) σ-complex is discarded. A metathesis mechanism was also rejected because chlorosilanes are not detected as reaction product. At this point, we undertook a theoretical study on the interaction between the hydrosilane and the [Mo_3_S_4_Cl_3_(ImNH_2_)_3_]^+^ cluster metal and/or sulfur atoms. All attempts to obtain stable adducts approaching the hydride of the silane toward the Mo atoms and the silicon atom toward the bridging sulfurs failed; therefore, a ligand assisted mechanism (see Figure 1) was also discarded. Based on this outcome, hydrosilane activation through sulfur–silicon and sulfur-hydride interactions, as illustrated in the catalytic cycle represented in Figure 6, was investigated. To do so, DFT calculations have been carried out at the UBP86-D3/BS2//UBP86-D3/BS1 level considering the solvent effects by means of PCM method. Further computational details can be found in Section S9.

**Figure 6:**
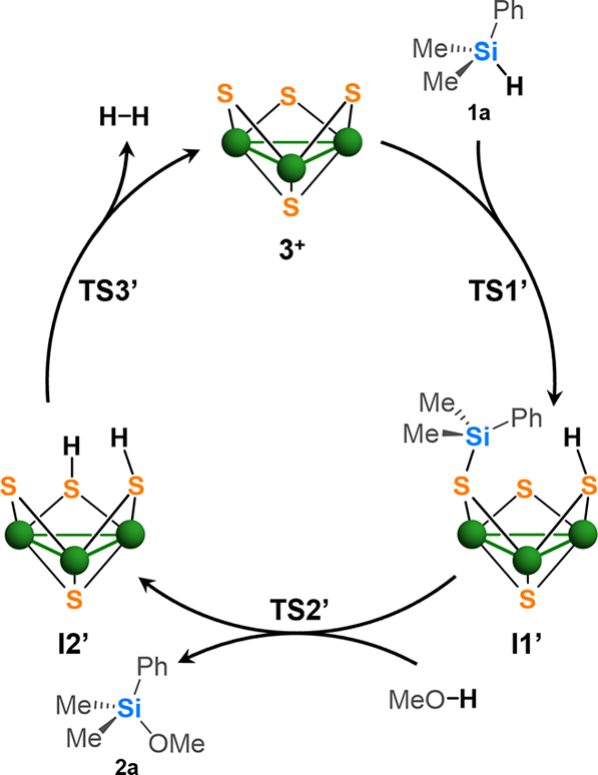
Tentative catalytic cycle for silane methanolysis by cluster **3** ^**+**^ through a stepwise mechanism.

This initial approach entails silane activation at the bridging sulfur atoms and the formation of an intermediate **I1’** bearing S–Si and S–H functionalities (see Figure 6). Next step involves a proton transfer from the methanol molecule to the third bridging sulfur atoms with the subsequent generation of methoxosilane (**2a**) and the bis­(hydrosulfido) **I2’** intermediate. Finally, hydrogen is released from this intermediate to recover the active cluster catalyst **3** ^**+**^. Despite we were able to elucidate a reaction pathway for this stepwise mechanism, the computational data did not align with the experimental results. An analysis of the Gibbs free energy barriers for this catalytic cycle (see Figure S16) shows that silane activation requires an energy barrier of 8.8 kcal·mol^–1^, while 26.1 kcal·mol^–1^ are needed for the second step. These computed energy values disagree with our experimental results which evidence that the rate determining step corresponds to the activation of silane as shown by the significant KIE effect observed when using PhMe_2_SiD instead of PhMe_2_SiH. Otherwise, we have considered other possible mechanism in which the methanol molecule is initially activated at the bridging sulfur atoms to yield a new intermediate containing S–O and S–H moieties. Unfortunately, the calculated energy barrier (Δ*G* ^‡^ = 40.8 kcal·mol^–1^) is not compatible with the mild experimental conditions (30 °C) required for this transformation, since Eyring analysis from kinetic experimental data shows that the activation barrier should be lower (Δ*G* ^‡^ = 22.3 kcal·mol^–1^), so that we also ruled out such possibility.

At this point, we considered an alternative sulfur-centered pathway inspired on the concerted mechanism reported by Huertos and coworkers.^18^ To do so, we postulate a transition state **TS1**, represented in Figure 7, where the silane and methanol molecules are pointing to the bridging sulfurs of the cluster core. This step, that connects reactants with the bis­(hydrosulfido) intermediate **I1** *via* **TS1**, is computed to occur with a free energy barrier of 24.7 kcal·mol^–1^, which aligns with the experimental barrier (Δ*G* ^‡^ = 22.3 kcal·mol^–1^). A reactant complex could be found after optimization of the intrinsic reaction coordinates (IRC) calculation from **TS1** which resulted in an intermediate lying 0.3 kcal·mol^–1^ above the separated reactants, thus being kinetically irrelevant. Analysis of the **TS1** Mulliken charges of the hydrogen atoms involved in the transference shows a negative charge for the silane hydrogen atom and a positive charge for the one bound to the oxygen atom of the methanol molecule (see Table S16). This suggests that hydrogen atoms are initially transferred to the cluster unit as a hydride and subsequently as a proton.

**Figure 7:**
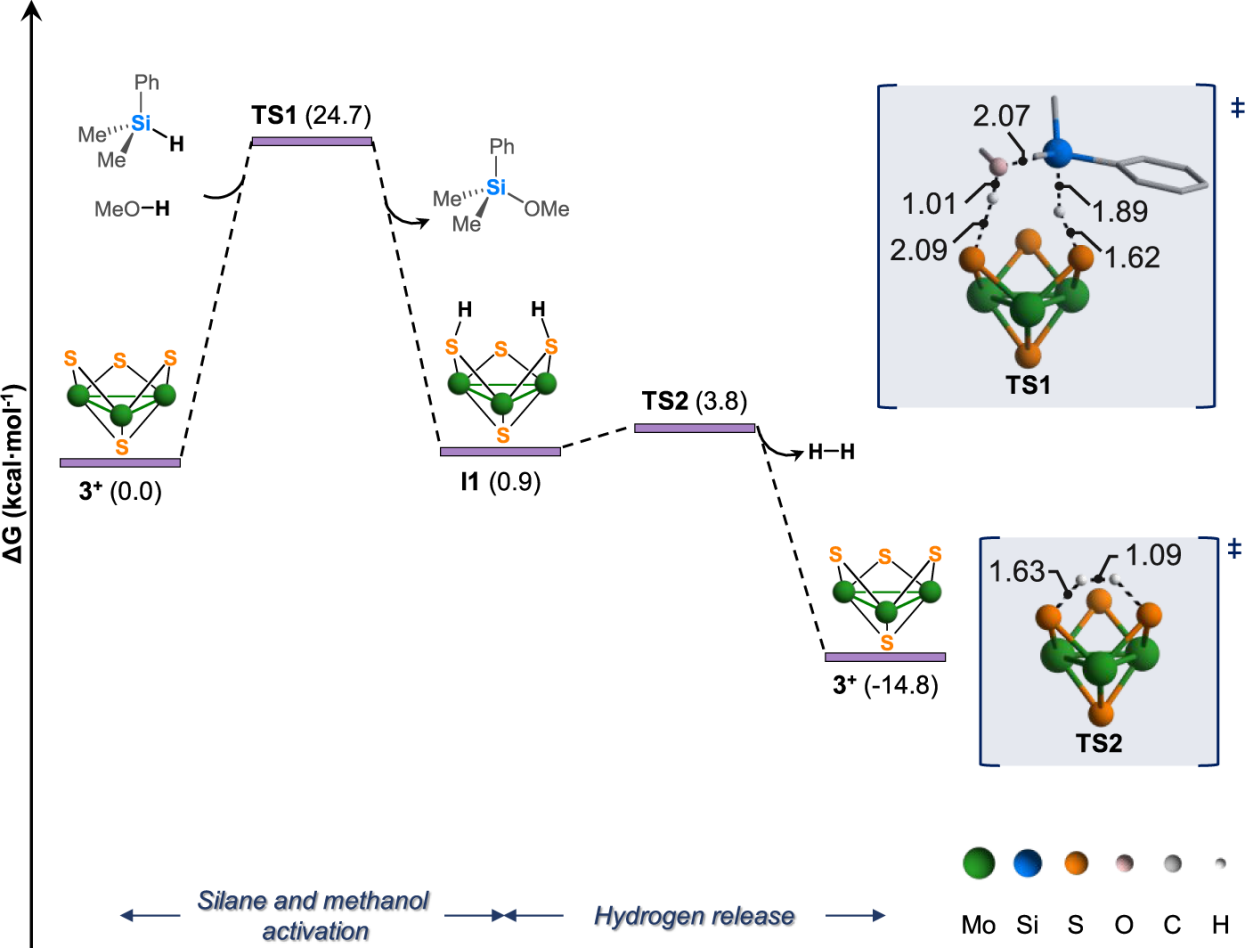
Gibbs energy profile for the methanolysis of dimethylphenylsilane catalyzed by cluster **3** ^**+**^. Free energies are given in kcal·mol^–1^, quoted relative to **3** ^**+**^ + dimethylphenylsilane + methanol. Ligands and hydrogen atoms are omitted for clarity. Selected bond distances are given in Å.

Structural analysis of this transition state shows a Si···H bond length of 1.89 Å, which indicates that the hydride is being transferred to the bridging sulfur. Although the proton of the methanol molecule points to the adjacent bridging sulfur atom, the O···H distance (1.01 Å) is consistent with the presence of an O–H bond. In fact, analysis of the imaginary eigenvalues evidence that this transition state is basically associated to the hydride transfer and the formation of the Si–O bond. The methanol proton is only transferred during the optimization of the last considered structure of the IRC. Thus, through this concerted but very asynchronous transition state, the hydride from the silane and the proton from the methanol molecules are transferred to the cluster leading to the formation of bis­(hydrosulfido) **I1** intermediate and the silyl ether product.

The computed bis­(hydrosulfido) **I1** presents a relative free energy of 0.9 kcal·mol^–1^. Analysis of the interatomic distances in the **I1** cluster intermediate reveals the shortening of one of the Mo–Mo bonds from 2.79 to 2.70 Å. This shortening has been previously attributed to the two electron reduction of the trimetallic Mo_3_^IV^ core with 6 CSE to Mo_2_^III^Mo^IV^ (8 CSE) with the concomitant formation of a double Mo–Mo bond.^23,24^ Finally, molecular hydrogen is released through **TS2** with a nondemanding Gibbs energy barrier of 2.9 kcal·mol^–1^ and the initial **3** ^**+**^ cluster catalyst is recovered.

According to these computational results, the rate determining step is attributed to **TS1**. This asynchronous transition state is mainly associated with the hydride transfer to the cluster core and the formation of the Si–O bond, which agrees with the first-order reaction kinetics. The negligible contribution from the O–H bond of the methanol to this process is in good agreement with the lack of KIE effect found when deuterated methanol is employed in the reaction. Additionally, theoretical KIEs were calculated using Eyring theory to provide additional support for this mechanism (see Section S9.3 for further details). Theoretical results are consistent with experimental observations, since the use of deuterated silane shows a significant effect on the reaction rate compared to deuterated methanol (KIE_Si**H**/Si**D**_ = 2.48; ${\mathrm{KIE}}_{{\mathrm{CH}}_{3}OH/{\mathrm{CD}}_{3}OD}=1.31$ ). The agreement between the calculated and experimental KIE values (KIE_Si**H**/Si**D**_ = 1.93; ${\mathrm{KIE}}_{{\mathrm{CH}}_{3}OH/{\mathrm{CD}}_{3}OD}=1.04$ ) is well within the limits of the so-called chemical accuracy, and, overall gives further support to the proposed mechanism.

We have also computed this mechanism using the UPBE0-D3 functional. Algarra and coworkers demonstrated that the use of this functional is an accurate method for the description of Mo_3_S_4_ clusters and its reactivity.^38^ Incidentally, UPBE0-D3 calculations revealed a splitting of the asynchronous **TS1** (see Section S9.2 for further details). Thus, after hydride transfer from the silane, an intermediate containing a silanol fragment is formed. Subsequently, proton is transferred to the cluster through a barrierless transition state. Unfortunately, in this case, silane activation is computed to require a higher barrier (Δ*G* ^‡^ = 34.3 kcal·mol^–1^), so the use of this functional is inconsistent with the experimental activation barrier (Δ*G* ^‡^ = 22.3 kcal·mol^–1^) (see Table S17).

Next, to gain further support to the reaction mechanism represented in Figure 7, we decided to compute the effect of the ancillary ligands on the catalytic activity of complexes **1** ^**+**^ and **2** ^**+**^ using the UBP86-D3 functional. The calculated reaction mechanism for both clusters (see Section S9.4) exhibits the same reaction profile as the one calculated for catalyst **3** ^**+**^. A comparative analysis of the free energy barriers needed to reach **TS1**, the rate determining step in the methanolysis of silanes, shows a good correlation between the evaluated catalytic activity **3** ^**+**^ > **1** ^**+**^ > **2** ^**+**^ (see Figure 4) and these free energy values. Reaching **TS1** requires relative free energies Δ*G* ^‡^ = 24.7, 25.6, and 27.5 kcal·mol^–1^ for **3** ^**+**^, **1** ^**+**^ and **2** ^**+**^, respectively. We attribute the higher catalytic performance of complex **3** ^**+**^ to the presence of π-interactions between the cluster bidentate ligand and the aromatic ring of the silane **1a**.

Finally, we analyzed the noncovalent interactions (NCI) within **TS1** (see Section S9.5). To do so, we calculated the NCI plots that correlate the electron density with the reduced density gradient. The resulting interactions are illustrated as colored isosurfaces where the red and blue regions correspond to repulsive and attractive interactions, whereas the green regions are indicative of van der Waals interactions. Notably, the presence of green isosurfaces, likely attributed to π-interactions, is confirmed between the organic ligand and the silane for each cluster as shown in Figure S18.^39,40^ Distance analysis of **TS1** in catalyst **3** ^**+**^ (see Figure S19) suggests partial π-stacking interactions between the Csp^2^ atoms in the C=C group of the imidazolyl ligand and the phenyl ring of the silane.^41^ In the case of clusters **1** ^**+**^ and **2** ^**+**^, N–H···π and C–H···π interactions are observed, respectively.^42^ Thus, the calculated free energies for **TS1** in each cluster are in accordance with the energy contributions of the π-interaction, following the order *E*_π‑stacking_ > *E*_NH‑π_ > *E*_CH‑π_.

## Conclusions

Reported reaction mechanisms for the homogeneous catalytic activation of hydrosilanes by transition metal complexes share the formation of metal hydride species during the catalytic cycle. In this work, we show for the first time an homogeneous Mo_3_(μ_3_-S)­(μ-S)_3_ metal cluster catalyst in which hydrosilane activation occurs without direct participation of the metals. To accomplish this, we integrated spectroscopic and spectrometric techniques, kinetic analyses that include KIE experiments, and control experiments with computational methodologies to formulate a reaction mechanism that presents a solid scientific foundation. For that purpose, the methanolysis of hydrosilanes was chosen as a model reaction based on previous findings of our group on the hydrogenation of nitroarenes using hydrosilanes. The reaction follows a two-step kinetic pathway, with the first one acting as the rate limiting step. Initially, hydrosilane activation occurs *via* a concerted but extremely asynchronous transition state **TS1** facilitated by the presence of a methanol molecule. In this step, the hydride of the silane and the proton of the methanol are transferred to two of the bridging sulfur atoms of the cluster core with calculated free energy barriers in line with the mild conditions required for the reaction. Examination of the transition state indicates that the imaginary frequency is mainly associated to the silane hydride transfer in agreement with the KIE experiements. This first step yields the silyl ether product and the bis­(hydrosulfido) intermediate **I1**. Subsequently, hydrogen is released from **I1** through the energetically nondemanding **TS2**, regenerating the diamimo or imidazolyl amino cluster catalyst. Calculations conducted on three different [Mo_3_S_4_Cl_3_(LL)_3_]^+^ cluster catalysts (LL = en, dmen, and ImNH_2_) confirm that an analogous mechanism operates independently of the ancillary ligand, while the calculated free energy barriers for the rate determining step are consistent with the observed catalytic activities giving further support to our mechanistic proposal.

## Supplementary Material



## Notes

Additional experimental details, crystallographic information, NMR and ESI-MS spectra, DFT results, and Cartesian coordinates for all optimized structures (PDF)

## Notes

Dedicated to Prof. Dr. Vladimir P. Fedin on the occasion of his 70th birth anniversary.
