# Supplementary material for: Sulfur-Centered Mechanism in Catalytic Methanolysis of Hydrosilanes Mediated by Air-Stable Mo3S4 Clusters
Source: Inorg Chem. 2025 Mar 21;64(13):6552–60. doi: 10.1021/acs.inorgchem.4c05438 (PMC12129257; doi:10.1021/acs.inorgchem.4c05438)
Supplement: Supplementary file 1 [file ic4c05438_si_001.pdf]

# Supporting Information

# Sulfur-Centered Mechanism in Catalytic Methanolysis of Hydrosilanes Mediated by Air-Stable $Mo_3S_4$ Clusters

*Juanjo Mateu-Campos,[a] María Gutiérrez-Blanco,[a] Eva Guillamón,[a] Vicent S. Safont,[a] Jordi Benet-Buchholz,[b] Mónica Oliva,*[a] and Rosa Llusar,*[a]*

[a] Departament de Química Física i Analítica, Universitat Jaume I. Av. Sos Baynat s/n, 12071 Castelló de la Plana, Spain

[b] Institute of Chemical Research of Catalonia - ICIQ, Av. Països Catalans 16, 43007 Tarragona, Spain

Corresponding authors email: oliva@uji.es, rosa.llusar@uji.es

## Table of contents

**1. Materials and methods ........................................................................................S2**

**2. Synthesis of cluster [1]Cl ........................................................................................S2**

**3. Crystallographic details........................................................................................S6**

**4. General procedures for the catalytic methanolysis of dimethylphenylsilane .................S16**

**5. Catalytic performance ........................................................................................S17**

**6. Synthesis of deuterated dimethylphenylsilane (1b)..........................................................S20**

**7. Hydrogen detection by $^1$H-NMR spectroscopy..........................................................S20**

**8. Kinetic Isotope Effect........................................................................................S21**

**9. Computational details........................................................................................S22**

**10. References ........................................................................................S75**

## 1. Materials and methods

All reactions were performed under nitrogen atmosphere, unless otherwise stated. Cluster complex $[Mo_3S_4Cl_3(ImNH_2)_3]Cl$ was prepared according to the published procedure replacing $HBF_4$ by HCl.[1] All other reagents were obtained from commercial sources and used as received or prepared according to the literature.

Elemental analyses were performed with a Euro EA 3000 Elemental Analyzer. UV-Visible spectra were recorded on an Agilent Cary 60 spectrophotometer. Mass spectra were registered in a QTOF Premier instrument operated in the V-mode at a resolution of *ca.* 10000 (FWHM) and a triple quadrupole mass spectrometer, both of them were equipped with an orthogonal Z-spray-electrospray interface (Waters, Manchester, UK). The temperature of the source block was set to 100 °C, and the desolvation temperature was set to 120 °C. A capillary voltage of 3.3 kV was used in the positive scan mode, and the cone voltage was set to Uc = 20 V. Sample solutions in $CH_3CN$ were injected with a syringe pump directly connected to the ESI source at a flow rate of 10 $\mu Lmin^{-1}$. The observed isotopic pattern of each compound perfectly matched the theoretical isotope pattern calculated from their elemental composition by using the MassLynx 4.1 program.[2] $^1H$, $^{13}C$, $^1H$-$^1H$ COSY NMR and $^1H$-$^{13}C$ gradient HSQC spectra were recorded on a Bruker Avance III HD 300 MHz or 400 MHz spectrometer. Gas chromatography analyses were performed on an Agilent 7820A GC System equipped with a FID and a capillary column Agilent (HP-5, 30m x 0.32mm x 0.25 µm). The GC yields were determined by GC-FID using anisole as an internal standard. Hydrogen evolution was monitored using a pressure transducer "Man on the Moon" series X104 kit.[3]

## 2. Synthesis of cluster [1]Cl

**Synthesis of $[Mo_3S_4Cl_3(en)_3]Cl$:** To a green solution of the trinuclear precursor $Mo_3S_4Cl_4(PPh_3)_3(H_2O)_2$ (500 mg, 0.362 mmol) in dry $CH_3CN$ (40 mL) was added a three-fold excess of the ligand ethylenediamine (80 µL, 1.191 mmol) under inert atmosphere. The reaction occurred with an immediate colour change to brown which gradually turns into a green suspension in 1-2 hours. After stirring the reaction mixture for 4 hours at room temperature the solid was filtered and washed with 30 mL of $CH_3CN$. Then, the solid was dissolved with $CH_3OH$ and concentrated under reduced pressure to obtain 50 mL of solution. Immediately, a freshly prepared methanolic solution of HCl (3.0 mL, 1.500 mmol) was added causing the partial precipitation of the desired product. Finally, the green solid was separated by filtration and washed with cold $CH_3OH$ (20 mL) and $CH_2Cl_2$ (50 mL) to remove the organic impurities to obtain 188.0 mg (70%) of the desired complex. **$^1$H-NMR** (400 MHz, DMSO-$d_6$): $\delta$ = 8.07 (m, 3H, NH, $H_A$), 6.84 (m, 3H, NH, $H_B$), 4.51 (m, 3H, $NH_2$, $H_C$), 3.54 (br, 3H, $CH_2$, $H_D$; 3H, $NH_2$,

$H_E$), 3.10 (br, 3H, $NH_2$, $H_F$), 2.67 (br, 6H, $CH_2$, $H_G$); **$^{13}$C-NMR** (101 MHz, DMSO-$d_6$): δ = 45.44 (s, $CH_2$, $C_1$), 45.27 (s, $CH_2$, $C_2$). **HRMS (ESI-TOF)** (20V, $CH_3CN$) *m/z* $[M]^+$ Calc for $Mo_3S_4C_6H_{24}N_6Cl_3$: 702.7156. Found 702.7170. **Elemental analysis** Calc. (%) for $Mo_3S_4C_6H_{24}N_6Cl_4$: C 9.8, H 3.3, N 11.4, S 17.4. Found C 9.2, H 3.6, N 11.2, S 16.9. **UV-Visible** ($5\cdot10^{-4}$ M, DMSO); λmax (ε, $L\cdot mol^{-1}\cdot cm^{-1}$) nm: 395 (6743), 656 (484) nm.

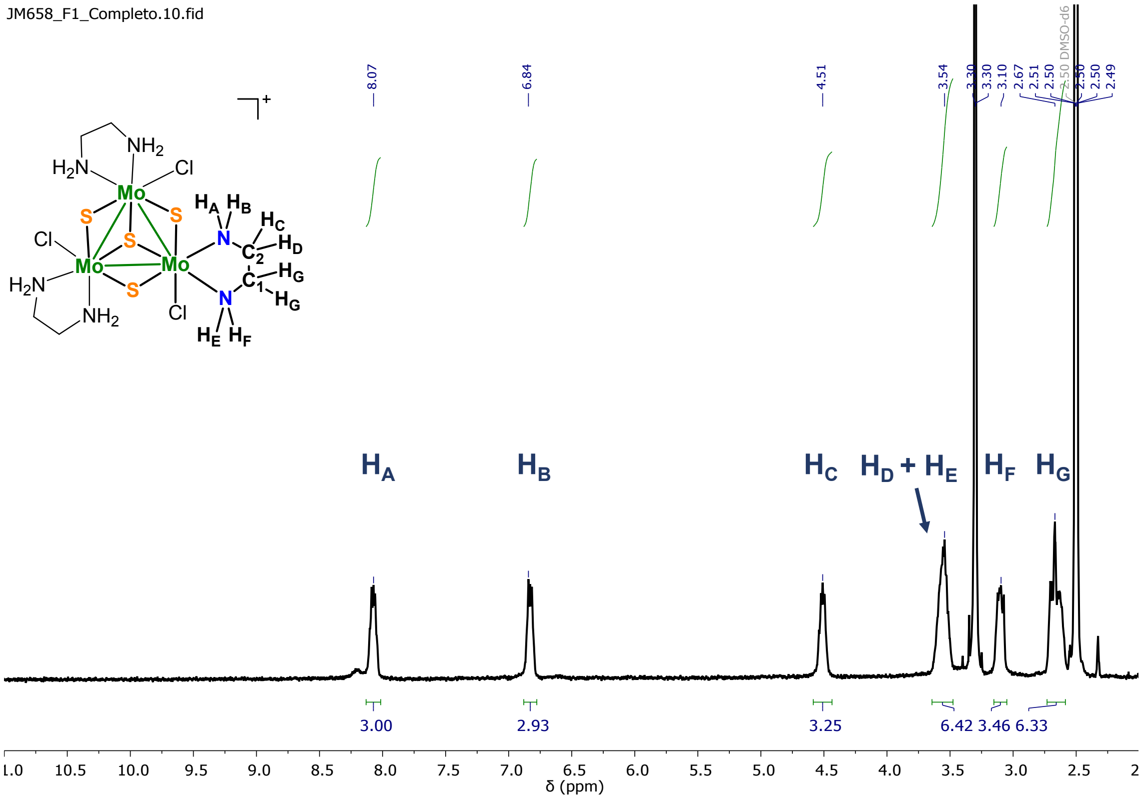


**Figure S1.** $^1$H-NMR spectrum of the $[Mo_3S_4Cl_3(en)_3]Cl$ complex in DMSO-$d_6$.

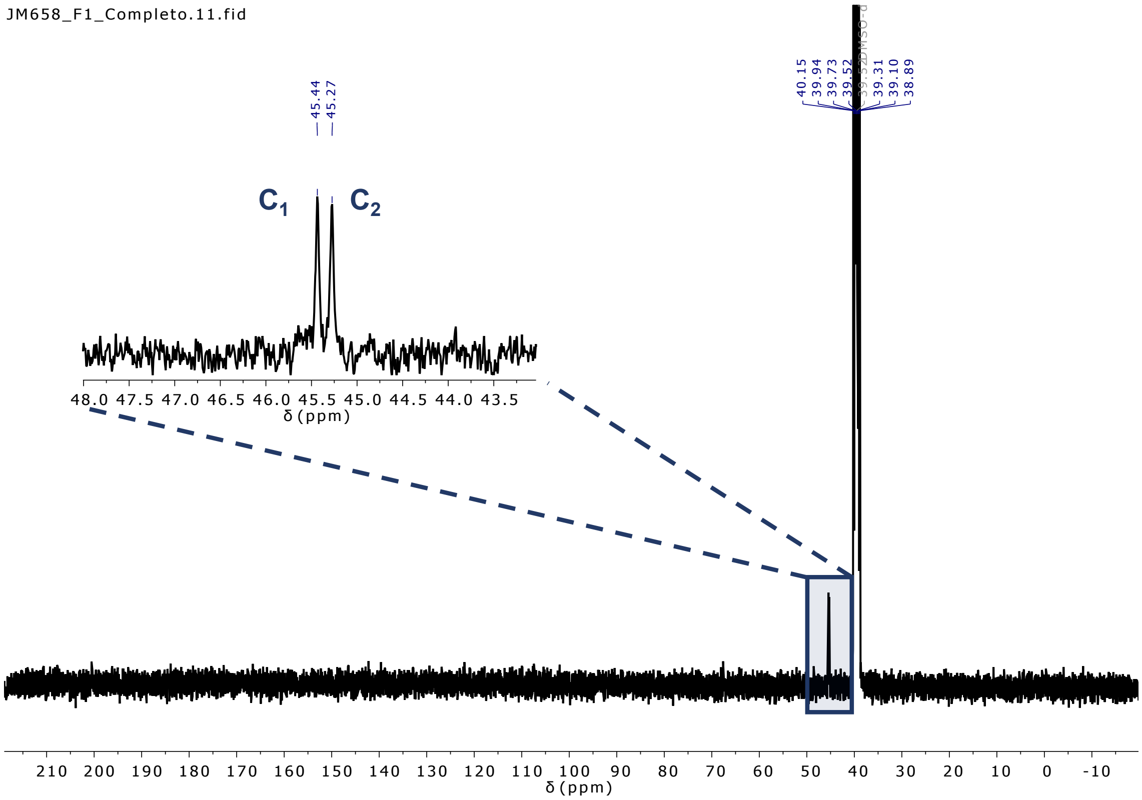


**Figure S2.** $^{13}$C-NMR spectrum of the $[Mo_3S_4Cl_3(en)_3]Cl$ complex in DMSO-$d_6$.

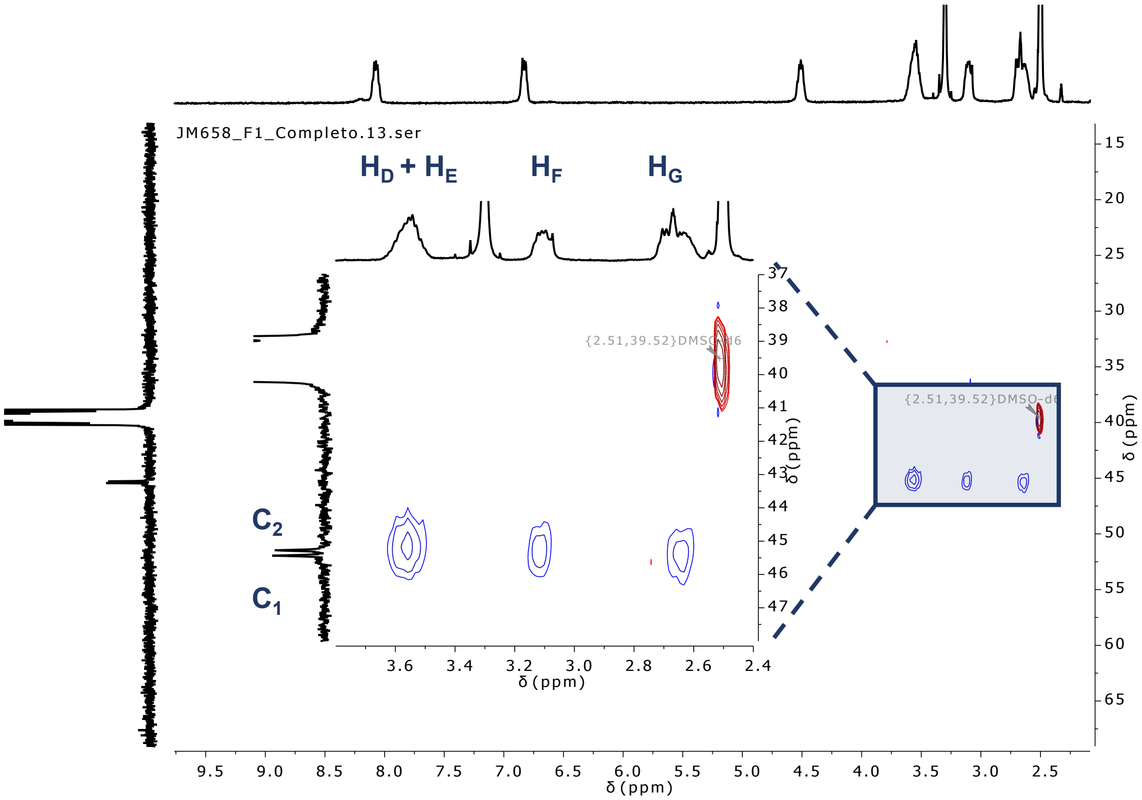


**Figure S3.** $^{1}$H-$^{13}$C gradient HSQC spectrum of the $[Mo_3S_4Cl_3(en)_3]Cl$ complex in DMSO-$d_6$.

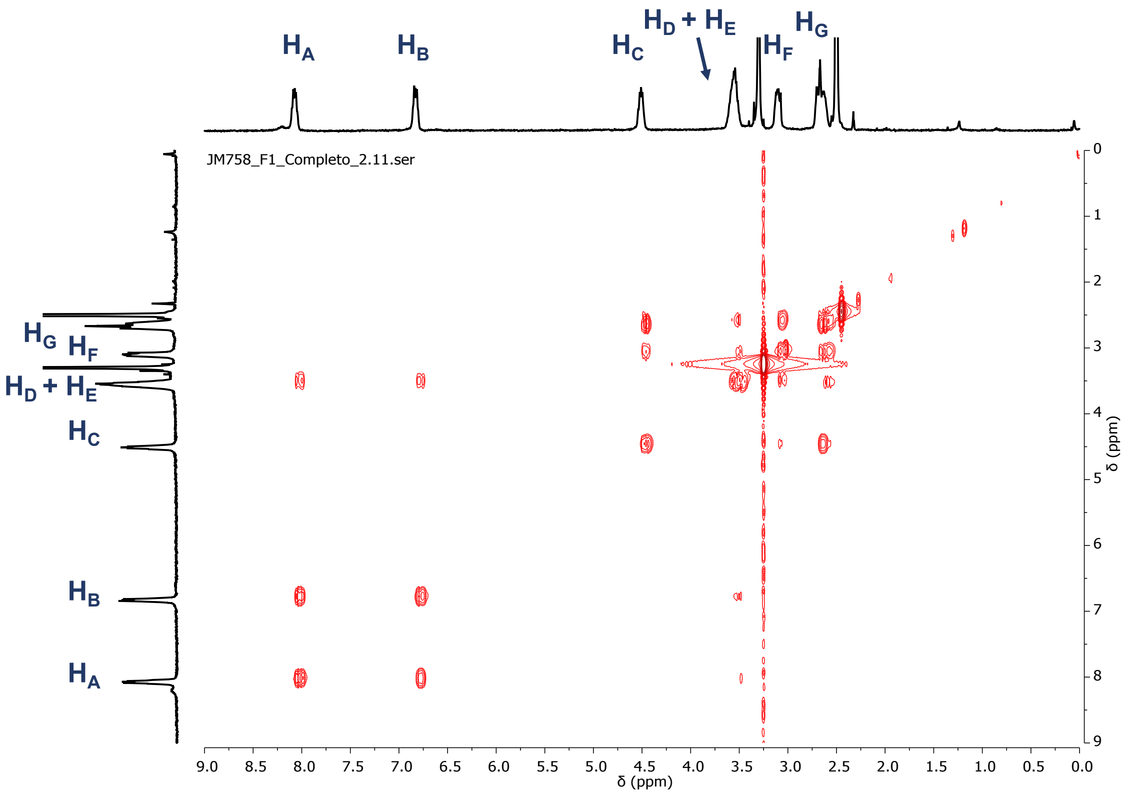


**Figure S4.** $^1$H-$^1$H COSY spectrum of the $[Mo_3S_4Cl_3(en)_3]Cl$ complex in DMSO-$d_6$.

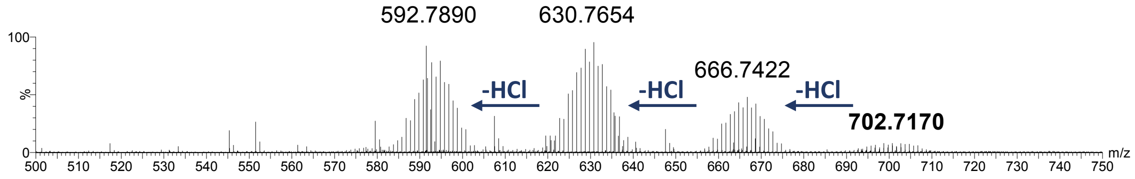


**Figure S5.** HRMS (ESI-TOF) spectrum of catalyst **1**$^+$ registered at 20 V in $CH_3CN$.

## 3. Crystallographic details

### Single Crystal Structure Determination *via* 3D Electron Diffraction of [1]Cl

Electron radiation features very strong interactions with the electrostatic potential of matter. If monochromatic electron radiation is applied to crystals in the nanometer size range, single crystal diffraction can be observed. Due to the strong interaction, data collected by using electron radiation are affected by dynamical scattering effects and by the ionic scattering factors. In structures refined using a simplistic kinematic approximation, these effects can lead to seemingly bad R-values.

**Sample preparation**

The sample used was available in a suspension in acetonitrile. Four 10 µl drops of this suspension were deposited carefully and consecutively with a micropipette on a copper-graphite grid (Lacey C only 200 TH Cu TED PELLA INC.). Once the acetonitrile was almost evaporated, the grid was inserted in the cryogenic bath at 215 K and mounted on the ELSA Gatan sample holder.

**Cryo-preparation**

The grid with the sample underwent cryo-preparation using an ELSA sample holder and the cryo-preparation stage from GATAN. In order to avoid ice formation and sublimate the residual acetonitrile, the sample was prepared at 215 K and mounted in the specimen chamber. The sample was held for 15 minutes at 215 K in the specimen chamber. After this the sample was cooled down. At 165 K and without interrupting the cooling, it was inserted into the column. The measurements were performed at 100 K.

**Device**

The sample grains were measured using a XtalLAB Synergy-ED from Rigaku-JEOL provided with a HyPix-ED detector and a LiB6 200 kV electron source (200 kV, 101.20 µA) in shutterless operation mode.[4] Wavelength: 0.0251 Å. Total dose for the three merged crystal grains: 22.618 $e^-/Å^2$, condenser strength of 1. Magnification diffraction: 50 cm, IL1 projection focus: hex 5301 (21245), CL 10 µm, SA 100 µm, distance (camera length): 643 mm, scan width: 0.25° and exposure time was depending on the crystal in the range 1.00-1.20 s/degree.

**Accessories:** ELSA (GATAN) sample holder and sample preparation stage for preparation and measurement of samples at low temperatures.

**Software:** Measurement and data processing: CrysAlis$^{Pro}$ 44.78a (Rigaku).[5] Structure processing: Olex2 Version 1.5-ac7-012 © OlexSys Ltd. 2004 - 2024.[6]

### 3.1. Measurement and refinement

The sample [**1**]Cl was explored on the XTalLAB Synergy-ED at 100 K. 11 crystals were measured at 100 K from which 6 could be indexed and gave the same trigonal cell.

Initial indexing was performed with the AutoChem 6 software system in combination with Olex 2 giving a trigonal cell with the space group *P*3c1 (No. 158).[7] Three other crystals could be indexed in the monoclinic space group $P2_1/c$. The crystals in the monoclinic cell resulted to be the same compound as the described structure in this paper, but instead of a $Cl^-$ salt it corresponded to an acetonitrile solvate of the $PF_6^-$ salt. These data were not published since they did not reach publishable quality. In addition, two more crystals could not be properly indexed. Unit cell parameters for the selected crystal in the trigonal cell are shown in Table S1.

**Table S1.** Trigonal unit cell (*P*3c1) indexed at 100 K for selected grains 05, 02 and 03.

| | Unit cell parameters | | | | | | Volume |
|---|---|---|---|---|---|---|---|
| **Grain** | **A (Å)** | **B (Å)** | **C (Å)** | **α (°)** | **β (°)** | **γ (°)** | **(Å$^3$)** |
| 05 | 15.401(4) | 15.401(4) | 11.4797(14) | 90 | 90 | 120 | 2358.2(8) |
| 02 | 15.380(3) | 15.380(3) | 11.504(4) | 90 | 90 | 120 | 2356.6(11) |
| 03 | 15.362(3) | 15.362(3) | 11.4801(18) | 90 | 90 | 120 | 2346.2(8) |

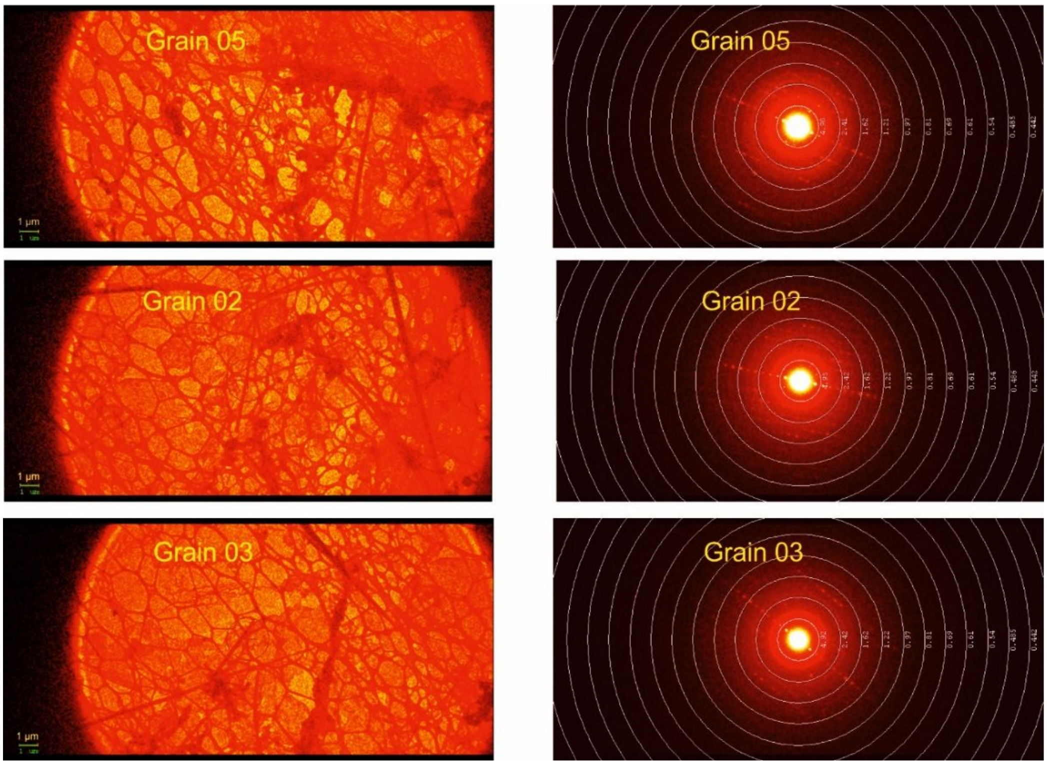


**Figure S6.** Grain snapshots and chosen diffraction images for all selected measurements. Grains 05, 02 and 03.

A structure solution was successfully obtained from several crystals measured at 100 K. One of the best datasets measured corresponded to crystal 05 which was used for structure solution using the program ShelXT.[8] (see Tables S5 to S8 for structure refinement results, R1: 13.45 % and Completeness: 100.0 %). The instrument was operated, and the diffraction data were processed in the program CrysAlis$^{Pro}$. Absorption correction was performed using spherical harmonics implemented in SCALE3 ABSPACK scaling algorithm in CrysAlis$^{Pro}$. To enhance the reflections-to-parameter ratio and reduce the dynamic effects, the datasets of the 3 best selected crystals were merged resulting in a single improved data set (Crystals: 05, 02 and 03). By merging data, a completeness of 100 % up to a resolution of 0.70 Å was achieved. An overview of data collection parameter and quality statistics for all single data collections is show in Table S2 and Table S3. Images and selected frames of the measured crystals are shown in Figure S6.

The merged dataset was used for structure refinement with a kinematical approximation using ShelXL in the crystallographic program suite OLEX2.[9] The structure refined to a final R1-value of 13.76 % (see Table S4 for overview and Tables S9 to S12). An ORTEP-plot drawing showing the asymmetric unit for structure [**1**]Cl is represented in Figure S7. The asymmetric unit contains two times 1/3 of the metal cluster, 2/3 of a chlorine anion, 0.5333 molecules of water and 0.2 molecules of acetonitrile. The two fragments of the main metal cluster al located on a 3-fold rotation axes and show $C_3$-symmetry. The chlorine atom is disordered sharing its position with the water molecules and the acetonitrile molecule. The water molecules are disordered in two positions with a ratio of 0.33333:0.2. The charge balance for each 1/3 of cluster in the asymmetric unit is +4 for Mo, -1 for Cl1A -2 for S1A, -0.6666 for 1/3 S2A and -0.3333 for 06666/2 Cl1S which gives a total of 0. Non-hydrogen atoms were assigned anisotropic displacement parameters unless stated otherwise. The hydrogen atoms were placed in idealized positions and included as riding. Isotropic displacement parameters for all H atoms were constrained to multiples of the equivalent displacement parameters of their parent atoms with $U_{iso}(H)$ = 1.2 (rigid H-atoms) or 1.5 (hydrogen atoms belonging to -OH) $U_{eq}$(parent atom). Enhanced rigid bond restraints, with standard uncertainties in the range of 0.01-0.005 Å$^2$ were applied.[10,11] The experimental details and the results of the kinematically refined data are given in Tables S5 to S12.

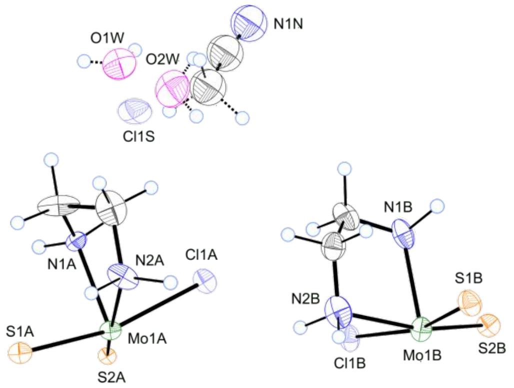


**Figure S7.** ORTEP-Drawing (thermal ellipsoids draw at 50% level) showing the atoms in the asymmetric unit of structure **[1]**Cl.

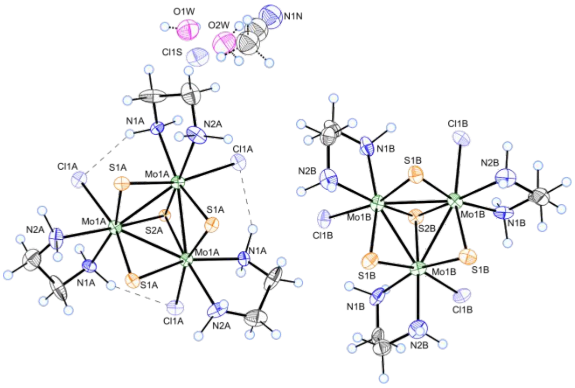

**Figure S8.** ORTEP-Drawing (thermal ellipsoids draw at 50% level) showing the molecules present in the crystal of **[1]**Cl after applying symmetry operations (Symmetry $C_3$ of the cluster).

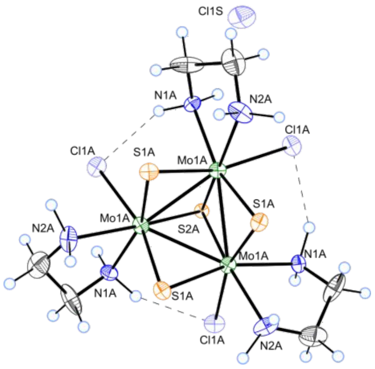


**Figure S9.** ORTEP-Drawing (thermal ellipsoids draw at 50% level) showing the **[1]**Cl cluster. Water and acetonitrile molecules were omitted for the sake of clarity.

**Table S2.** Data collection parameter overview.

| Dataset (grain) | Number of frames | Scan range (°) | Scan width (°) | Exposure time (min) | Total time (min:s) | Total Dose ($e^-/Å^2$) |
|---|---|---|---|---|---|---|
| 05 | 448 | -62 to +49.75 | 0.25 | 1.20 | 09:20 | 8.400 |
| 02 | 300 | -40 to+34.75 | 0.25 | 1.00 | 05:11 | 4.500 |
| 03 | 488 | -60 to +61.75 | 0.25 | 1.00 | 08:19 | 7.320 |
| **Merged crystals:** 05, 02, 03 | | | | Total: | 22:50 | 22.618 |

**Table S3.** Data quality statistics overview for selected single data collections and merged data for crystals 05, 02 and 03 (resolution of 0.80 Å). Point group symmetry: $P2_1$.

| Dataset (grain) | Data Fo>4σ | Compl. (%) | Redund. | $<F^2>$ | $<F^2/\sigma(F^2)>$ | $R_{int}$ | $R_{rim}$ | $R_{pim}$ | $CC^*$ |
|---|---|---|---|---|---|---|---|---|---|
| 05 | 2235 | 100 | 6.1 | 1987.91 | 5.30 | 0.198 | 0.205 | 0.086 | 0.996 |
| 02 | 1857 | 99.4 | 4.3 | 2963.89 | 4.74 | 0.174 | 0.178 | 0.093 | 0.992 |
| 03 | 2576 | 100 | 6.6 | 1962.98 | 4.91 | 0.220 | 0.238 | 0.094 | 0.992 |
| **Merged crystals: 05, 02, 03** | 3379 | 100 | 15.4 | 2137.58 | 7.17 | 0.254 | 0.271 | 0.066 | 0.992 |

**Table S4.** Kinematical refinement parameter for all single data collections and merged data.

| Dataset (grain) | $R_{int}$ Olex (%) | R1 (%) | wR2 (%) | Resolution (Å) | Resolution (I/σ(I)) |
|---|---|---|---|---|---|
| **05** | 15.68 | 13.45 | 30.66 | 0.80 | 7.8 |
| **02** | 17.60 | 13.90 | 33.11 | 0.80 | 5.4 |
| **03** | 21.82 | 13.63 | 36.75 | 0.70 | 5.2 |
| **Merged crystals 05, 02, 03** | 25.91 | 13.76 | 31.46 | 0.70 | 8.1 |

## 3.2. Data tables

### Tables for crystal 05

**Table S5.** Crystal data and structure refinement for JM1175A2LT05_JBB80

| CRYSTAL 05 | |
|---|---|
| **Identification code** | JM1175A2LT05_JBB80 |
| **Empirical formula** | $C_{13.2}H_{53}Cl_8Mo_6N_{12.6}O_{1.6}S_8$ |
| **Formula weight** | 1529.85 |
| **Temperature/K** | 102.4 |
| **Crystal system** | trigonal |
| **Space group** | *P*3c1 |
| **a/Å** | 15.401(4) |
| **b/Å** | 15.401(4) |
| **c/Å** | 11.4797(14) |
| **α/°** | 90 |
| **β/°** | 90 |
| **γ/°** | 120 |
| **Volume/Å$^3$** | 2358.2(11) |
| **Z** | 2 |
| **$\rho_{calc}$g/cm$^3$** | 2.155 |
| **μ/mm$^{-1}$** | 0.000 |
| **F(000)** | 468.0 |
| **Crystal size/mm$^3$** | 0.005 × 0.0001 × 0.00007 |
| **Radiation** | Electron radiation (λ = 0.0251) |
| **2Θ range for data collection/°** | 0.108 to 1.798 |
| **Index ranges** | $-19 \leq h \leq 19$, $-19 \leq k \leq 19$, $-14 \leq l \leq 14$ |
| **Reflections collected** | 10508 |
| **Independent reflections** | 3219 [$R_{int}$ = 0.1568, $R_{sigma}$ = 0.1283] |
| **Data/restraints/parameters** | 3219/197/176 |
| **Goodness-of-fit on F$^2$** | 1.507 |
| **Final R indexes [I>=2σ (I)]** | $R_1$ = 0.1345, $wR_2$ = 0.2904 |
| **Final R indexes [all data]** | $R_1$ = 0.1680, $wR_2$ = 0.3066 |
| **Largest diff. peak/hole / e Å$^{-3}$** | 0.45/-0.42 |

**Table S6.** Bond lengths for JM1175A2LT05_JBB80

| CRYSTAL 05 | | | | | |
|---|---|---|---|---|---|
| **Atom** | **Atom** | **Length/Å** | **Atom** | **Atom** | **Length/Å** |
| Mo1A | Mo1A$^{1}$ | 2.713(7) | Mo1B | Mo1B$^{4}$ | 2.728(8) |
| Mo1A | Mo1A$^{2}$ | 2.713(7) | Mo1B | Cl1B | 2.472(11) |
| Mo1A | Cl1A | 2.512(9) | Mo1B | S1B$^{3}$ | 2.278(13) |
| Mo1A | S1A | 2.254(10) | Mo1B | S1B | 2.270(12) |
| Mo1A | S1A$^{1}$ | 2.270(10) | Mo1B | S2B | 2.317(11) |
| Mo1A | S2A | 2.331(11) | Mo1B | N1B | 2.23(2) |
| Mo1A | N1A | 2.242(18) | Mo1B | N2B | 2.20(2) |
| Mo1A | N2A | 2.205(18) | N1B | C1B | 1.44(3) |
| N1A | C1A | 1.44(3) | N2B | C2B | 1.47(3) |
| N2A | C2A | 1.54(3) | C1B | C2B | 1.53(3) |
| C1A | C2A | 1.55(4) | C1N | C2N | 1.404(15) |
| Mo1B | Mo1B3 | 2.728(8) | C2N | N1N | 1.101(14) |
| $^{1}$-Y,+X-Y,+Z; $^{2}$+Y-X,-X,+Z; $^{3}$+Y-X,1-X,+Z; $^{4}$1-Y,1+X-Y,+Z | | | | | |

**Table S7.** Bond angles for JM1175A2LT05_JBB80

| CRYSTAL 05 | | | | | | | |
|---|---|---|---|---|---|---|---|
| **Atom** | **Atom** | **Atom** | **Angle/°** | **Atom** | **Atom** | **Atom** | **Angle/°** |
| Mo1A$^{1}$ | Mo1A | Mo1A$^{2}$ | 60.0 | Cl1B | Mo1B | Mo1B$^{3}$ | 139.9(3) |
| Cl1A | Mo1A | Mo1A$^{2}$ | 97.0(3) | Cl1B | Mo1B | Mo1B$^{4}$ | 97.8(4) |
| Cl1A | Mo1A | Mo1A$^{1}$ | 140.1(2) | S1B | Mo1B | Mo1B$^{4}$ | 98.4(3) |
| S1A | Mo1A | Mo1A$^{2}$ | 98.0(3) | S1B$^{4}$ | Mo1B | Mo1B$^{4}$ | 53.0(3) |
| S1A | Mo1A | Mo1A$^{1}$ | 53.4(3) | S1B$^{4}$ | Mo1B | Mo1B$^{3}$ | 98.2(3) |
| S1A$^{2}$ | Mo1A | Mo1A$^{2}$ | 52.9(3) | S1B | Mo1B | Mo1B$^{3}$ | 53.3(3) |
| S1A$^{2}$ | Mo1A | Mo1A$^{1}$ | 97.6(3) | S1B | Mo1B | Cl1B | 163.6(4) |
| S1A$^{2}$ | Mo1A | Cl1A | 90.9(3) | S1B$^{4}$ | Mo1B | Cl1B | 91.3(4) |
| S1A | Mo1A | Cl1A | 164.4(4) | S1B | Mo1B | S1B$^{4}$ | 96.1(6) |
| S1A | Mo1A | S1A$^{2}$ | 94.7(5) | S1B$^{4}$ | Mo1B | S2B | 105.6(4) |
| S1A$^{2}$ | Mo1A | S2A | 106.1(3) | S1B | Mo1B | S2B | 105.9(4) |
| S1A | Mo1A | S2A | 106.6(3) | S2B | Mo1B | Mo1B$^{4}$ | 53.9(2) |
| S2A | Mo1A | Mo1A$^{1}$ | 54.42(19) | S2B | Mo1B | Mo1B$^{3}$ | 53.9(2) |
| S2A | Mo1A | Mo1A$^{2}$ | 54.41(19) | S2B | Mo1B | Cl1B | 86.0(3) |
| S2A | Mo1A | Cl1A | 85.8(3) | N1B | Mo1B | Mo1B$^{4}$ | 141.6(5) |
| N1A | Mo1A | Mo1A$^{2}$ | 143.7(4) | N1B | Mo1B | Mo1B$^{3}$ | 97.8(6) |
| N1A | Mo1A | Mo1A$^{1}$ | 99.0(5) | N1B | Mo1B | Cl1B | 79.5(6) |

| | | | | | | | |
|---|---|---|---|---|---|---|---|
| N1A | Mo1A | Cl1A | 80.8(5) | N1B | Mo1B | S1B | 89.6(6) |
| N1A | Mo1A | S1A | 89.7(5) | N1B | Mo1B | S1B[4] | 163.3(7) |
| N1A | Mo1A | S1A[2] | 162.0(5) | N1B | Mo1B | S2B | 87.7(5) |
| N1A | Mo1A | S2A | 89.3(4) | N2B | Mo1B | Mo1B[4] | 141.5(6) |
| N2A | Mo1A | Mo1A[2] | 139.6(6) | N2B | Mo1B | Mo1B[3] | 136.7(6) |
| N2A | Mo1A | Mo1A[1] | 137.8(6) | N2B | Mo1B | Cl1B | 82.1(6) |
| N2A | Mo1A | Cl1A | 81.4(6) | N2B | Mo1B | S1B | 83.5(6) |
| N2A | Mo1A | S1A | 84.4(6) | N2B | Mo1B | S1B[4] | 88.5(6) |
| N2A | Mo1A | S1A[2] | 86.7(6) | N2B | Mo1B | S2B | 161.8(6) |
| N2A | Mo1A | S2A | 162.0(5) | N2B | Mo1B | N1B | 76.6(8) |
| N2A | Mo1A | N1A | 76.3(6) | Mo1B | S1B | Mo1B[3] | 73.7(4) |
| Mo1A | S1A | Mo1A[1] | 73.7(3) | Mo1B[4] | S2B | Mo1B[3] | 72.1(4) |
| Mo1A[1] | S2A | Mo1A[2] | 71.2(4) | Mo1B | S2B | Mo1B[3] | 72.1(4) |
| Mo1A | S2A | Mo1A[1] | 71.2(4) | Mo1B | S2B | Mo1B[4] | 72.1(4) |
| Mo1A | S2A | Mo1A[2] | 71.2(4) | C1B | N1B | Mo1B | 111.7(15) |
| C1A | N1A | Mo1A | 110.7(12) | C2B | N2B | Mo1B | 111.0(15) |
| C2A | N2A | Mo1A | 111.9(12) | N1B | C1B | C2B | 110(2) |
| N1A | C1A | C2A | 106(2) | N2B | C2B | C1B | 106.9(19) |
| N2A | C2A | C1A | 106(2) | N1N | C2N | C1N | 165(10) |
| Mo1B[3] | Mo1B | Mo1B[4] | 60.000(1) | | | | |

[1]+Y-X,-X,+Z; [2]-Y,+X-Y,+Z; [3]1-Y,1+X-Y,+Z; [4]+Y-X,1-X,+Z

**Table S8.** Torsion angles for JM1175A2LT05_JBB80

| **CRYSTAL 05** | | | | | | | | | |
|---|---|---|---|---|---|---|---|---|---|
| **A** | **B** | **C** | **D** | **Angle/º** | **A** | **B** | **C** | **D** | **Angle/º** |
| Mo1A | N1A | C1A | C2A | 51(2) | Mo1B | N1B | C1B | C2B | 37(2) |
| Mo1A | N2A | C2A | C1A | 38(2) | Mo1B | N2B | C2B | C1B | 45(2) |
| N1A | C1A | C2A | N2A | 58(2) | N1B | C1B | C2B | N2B | 54(3) |

**Tables for Merged data crystals 05, 02 and 03**

**Table S9**. Crystal data and structure refinament for JM1175A2LT_merge050203_JBB70

| MERGED CRYSTALS 05, 03, 02 | |
|---|---|
| **Identification code** | JM1175A2LT_merge050203_JBB70 |
| **Empirical formula** | $C_{13.2}H_{53}Cl_8Mo_6N_{12.6}O_{1.6}S_8$ |
| **Formula weight** | 1529.85 |
| **Temperature/K** | 102 |
| **Crystal system** | trigonal |
| **Space group** | P3c1 |
| **a/Å** | 15.417(4) |
| **b/Å** | 15.417(4) |
| **c/Å** | 11.4897(16) |
| **α/°** | 90 |
| **β/°** | 90 |
| **γ/°** | 120 |
| **Volume/Å$^3$** | 2365.2(12) |
| **Z** | 2 |
| **$\rho_{calc}$g/cm$^3$** | 2.148 |
| **μ/mm$^{-1}$** | 0.000 |
| **F(000)** | 468.0 |
| **Crystal size/mm$^3$** | 0.005 × 0.0001 × 0.00007 |
| **Radiation** | Electron radiation (λ = 0.0251) |
| **2Θ range for data collection/°** | 0.108 to 2.054 |
| **Index ranges** | $-22 \leq h \leq 22$, $-22 \leq k \leq 22$, $-16 \leq l \leq 16$ |
| **Reflections collected** | 38734 |
| **Independent reflections** | 4808 [$R_{int}$ = 0.2591, $R_{sigma}$ = 0.1239] |
| **Data/restraints/parameters** | 4808/46/176 |
| **Goodness-of-fit on F$^2$** | 1.494 |
| **Final R indexes [I>=2σ (I)]** | $R_1$ = 0.1370, $wR_2$ = 0.2954 |
| **Final R indexes [all data]** | $R_1$ = 0.1673, $wR_2$ = 0.3109 |
| **Largest diff. peak/hole / e Å$^{-3}$** | 0.46/-0.39 |

**Table S10**. Bond lengths for JM1175A2LT_merge050203_JBB70

| MERGED CRYSTALS 05, 03, 02 | | | | | |
|---|---|---|---|---|---|
| **Atom** | **Atom** | **Length/Å** | **Atom** | **Atom** | **Length/Å** |
| Mo1A | Mo1A[1] | 2.711(5) | Mo1B | Mo1B[4] | 2.726(5) |
| Mo1A | Mo1A[2] | 2.711(5) | Mo1B | Cl1B | 2.495(8) |
| Mo1A | Cl1A | 2.508(7) | Mo1B | S1B[4] | 2.287(8) |
| Mo1A | S1A | 2.260(7) | Mo1B | S1B | 2.267(8) |
| Mo1A | S1A[2] | 2.271(7) | Mo1B | S2B | 2.318(8) |
| Mo1A | S2A | 2.338(7) | Mo1B | N1B | 2.270(16) |
| Mo1A | N1A | 2.248(12) | Mo1B | N2B | 2.233(15) |
| Mo1A | N2A | 2.212(12) | N1B | C1B | 1.45(2) |
| N1A | C1A | 1.439(19) | N2B | C2B | 1.49(2) |
| N2A | C2A | 1.55(2) | C1B | C2B | 1.53(2) |
| C1A | C2A | 1.54(3) | C1N | C2N | 1.406(14) |
| Mo1B | Mo1B[3] | 2.726(5) | C2N | N1N | 1.107(14) |
| [1]+Y-X,-X,+Z; [2]-Y,+X-Y,+Z; [3]1-Y,1+X-Y,+Z; [4]+Y-X,1-X,+Z | | | | | |

**Table S11.** Bond angles for JM1175A2LT_merge050203_JBB70

| MERGED CRYSTALS 05, 03, 02 | | | | | | | |
|---|---|---|---|---|---|---|---|
| **Atom** | **Atom** | **Atom** | **Angle/°** | **Atom** | **Atom** | **Atom** | **Angle/°** |
| Mo1A[1] | Mo1A | Mo1A[2] | 60.0 | Cl1B | Mo1B | Mo1B[4] | 140.42(18) |
| Cl1A | Mo1A | Mo1A[2] | 140.13(16) | Cl1B | Mo1B | Mo1B[3] | 97.9(2) |
| Cl1A | Mo1A | Mo1A[1] | 97.3(2) | S1B | Mo1B | Mo1B[4] | 53.6(2) |
| S1A[1] | Mo1A | Mo1A[1] | 53.0(2) | S1B[3] | Mo1B | Mo1B[4] | 98.0(2) |
| S1A[1] | Mo1A | Mo1A[2] | 97.9(2) | S1B[3] | Mo1B | Mo1B[3] | 52.9(2) |
| S1A | Mo1A | Mo1A[2] | 53.4(2) | S1B | Mo1B | Mo1B[3] | 98.5(2) |
| S1A | Mo1A | Mo1A[1] | 98.2(2) | S1B[3] | Mo1B | Cl1B | 91.1(3) |
| S1A | Mo1A | Cl1A | 164.0(3) | S1B | Mo1B | Cl1B | 163.1(3) |
| S1A[1] | Mo1A | Cl1A | 90.9(2) | S1B | Mo1B | S1B[3] | 95.9(4) |
| S1A | Mo1A | S1A[1] | 95.2(3) | S1B[3] | Mo1B | S2B | 105.6(3) |
| S1A | Mo1A | S2A | 106.7(2) | S1B | Mo1B | S2B | 106.2(2) |
| S1A[1] | Mo1A | S2A | 106.3(2) | S1B | Mo1B | N1B | 89.7(4) |
| S2A | Mo1A | Mo1A[2] | 54.56(12) | S2B | Mo1B | Mo1B[4] | 53.98(15) |
| S2A | Mo1A | Mo1A[1] | 54.56(12) | S2B | Mo1B | Mo1B[3] | 53.99(14) |
| S2A | Mo1A | Cl1A | 85.6(2) | S2B | Mo1B | Cl1B | 86.5(2) |
| N1A | Mo1A | Mo1A[2] | 99.1(3) | N1B | Mo1B | Mo1B[3] | 141.5(4) |
| N1A | Mo1A | Mo1A[1] | 143.9(3) | N1B | Mo1B | Mo1B[4] | 97.8(4) |

| | | | | | | | |
|---|---|---|---|---|---|---|---|
| N1A | Mo1A | Cl1A | 80.5(4) | N1B | Mo1B | Cl1B | 79.6(4) |
| N1A | Mo1A | S1A[1] | 161.6(4) | N1B | Mo1B | S1B[3] | 163.5(4) |
| N1A | Mo1A | S1A | 89.2(4) | N1B | Mo1B | S2B | 87.6(4) |
| N1A | Mo1A | S2A | 89.4(3) | N2B | Mo1B | Mo1B[3] | 141.0(4) |
| N2A | Mo1A | Mo1A[2] | 138.0(4) | N2B | Mo1B | Mo1B[4] | 137.6(4) |
| N2A | Mo1A | Mo1A[1] | 139.3(4) | N2B | Mo1B | Cl1B | 80.8(4) |
| N2A | Mo1A | Cl1A | 81.1(4) | N2B | Mo1B | S1B[3] | 88.1(5) |
| N2A | Mo1A | S1A[1] | 86.3(4) | N2B | Mo1B | S1B | 84.2(5) |
| N2A | Mo1A | S1A | 84.6(4) | N2B | Mo1B | S2B | 161.5(4) |
| N2A | Mo1A | S2A | 161.9(3) | N2B | Mo1B | N1B | 77.0(5) |
| N2A | Mo1A | N1A | 76.4(5) | Mo1B | S1B | Mo1B[4] | 73.5(3) |
| Mo1A | S1A | Mo1A[2] | 73.5(2) | Mo1B[3] | S2B | Mo1B | 72.0(3) |
| Mo1A | S2A | Mo1A[2] | 70.9(2) | Mo1B[3] | S2B | Mo1B[4] | 72.0(3) |
| Mo1A[2] | S2A | Mo1A[1] | 70.9(2) | Mo1B[4] | S2B | Mo1B | 72.0(3) |
| Mo1A | S2A | Mo1A[1] | 70.9(2) | C1B | N1B | Mo1B | 110.6(10) |
| C1A | N1A | Mo1A | 111.8(8) | C2B | N2B | Mo1B | 108.8(10) |
| C2A | N2A | Mo1A | 111.5(8) | N1B | C1B | C2B | 109.7(14) |
| N1A | C1A | C2A | 106.5(15) | N2B | C2B | C1B | 107.5(13) |
| C1A | C2A | N2A | 107.3(14) | N1N | C2N | C1N | 161(9) |
| Mo1B[3] | Mo1B | Mo1B[4] | 60.000(1) | | | | |
| [1]-Y,+X-Y,+Z; [2]+Y-X,-X,+Z; [3]+Y-X,1-X,+Z; [4]1-Y,1+X-Y,+Z | | | | | | | |

**Table S12.** Torsion angles for JM1175A2LT_merge050203_JBB70

| MERGED CRYSTALS 05, 03, 02 | | | | | | | | | |
|---|---|---|---|---|---|---|---|---|---|
| **A** | **B** | **C** | **D** | **Angle/º** | **A** | **B** | **C** | **D** | **Angle/º** |
| Mo1A | N1A | C1A | C2A | 48.5(15) | Mo1B | N1B | C1B | C2B | 37.0(14) |
| Mo1A | N2A | C2A | C1A | 37.3(16) | Mo1B | N2B | C2B | C1B | 47.8(15) |
| N1A | C1A | C2A | N2A | -55.8(16) | N1B | C1B | C2B | N2B | -57.1(17) |

## 4. General procedures for the catalytic methanolysis of dimethylphenylsilane

A closed reaction vessel (13.2 mL) containing a stirring bar in nitrogen atmosphere was charged with the molybdenum catalyst **3**$^+$ (0.0050 mmol of [$Mo_3S_4Cl_3(ImNH_2)_3$]Cl), internal standard (50 µL of anisole) and 2 mL of dried and degassed MeOH. Afterwards, the reaction vessel was immersed in a thermostated silicon bath (30 ºC) for 20 minutes and then, the silane (0.5 mmol) was added. After the corresponding reaction time, ethyl acetate (2 mL) was added, and a sample was taken to be analyzed by GC.

In the case of hydrogen monitoring, the same described procedure was followed without addition of the internal standard. The reaction vessel was connected to an inverted burette setup or a pressure transducer (Man on the Moon series X104 kit). In this case, the reaction mixture was immersed in the thermostated (30 ºC) bath until the gas volume or pressure was stabilized, then the silane was added (0.5 mmol). The solution was stirred until the gas volume or pressure stabilized again, which was indicative that the reaction ended. The amount of hydrogen evolved was calculated from the collected gas or measured pressure inside the reaction vessel following the ideal gases law equation.

## 5. Catalytic performance

### 5.1. Catalyst monitoring

The integrity of cluster **3**$^{+}$ was assessed by ESI-MS spectrometry of the catalytic samples extracted at different times. In Figure S10 is represented the cluster monitoring during the reaction course.

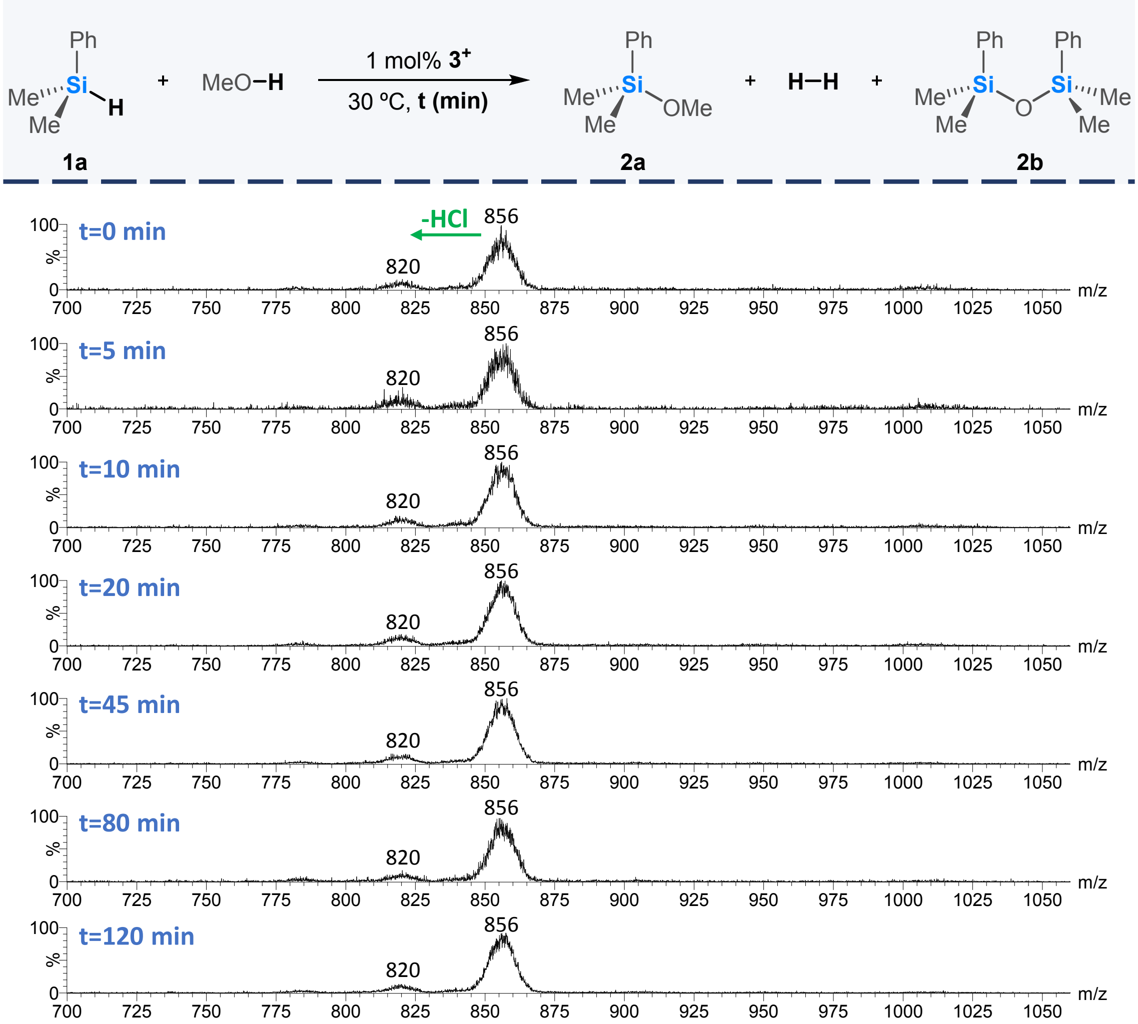

**Figure S10.** ESI-MS spectra of catalyst **3**$^+$ during silane methanolysis at different times. Spectra registered at 20 V in $CH_3CN$.

### 5.2. Cluster reactivity in front of dimethylphenylsilane

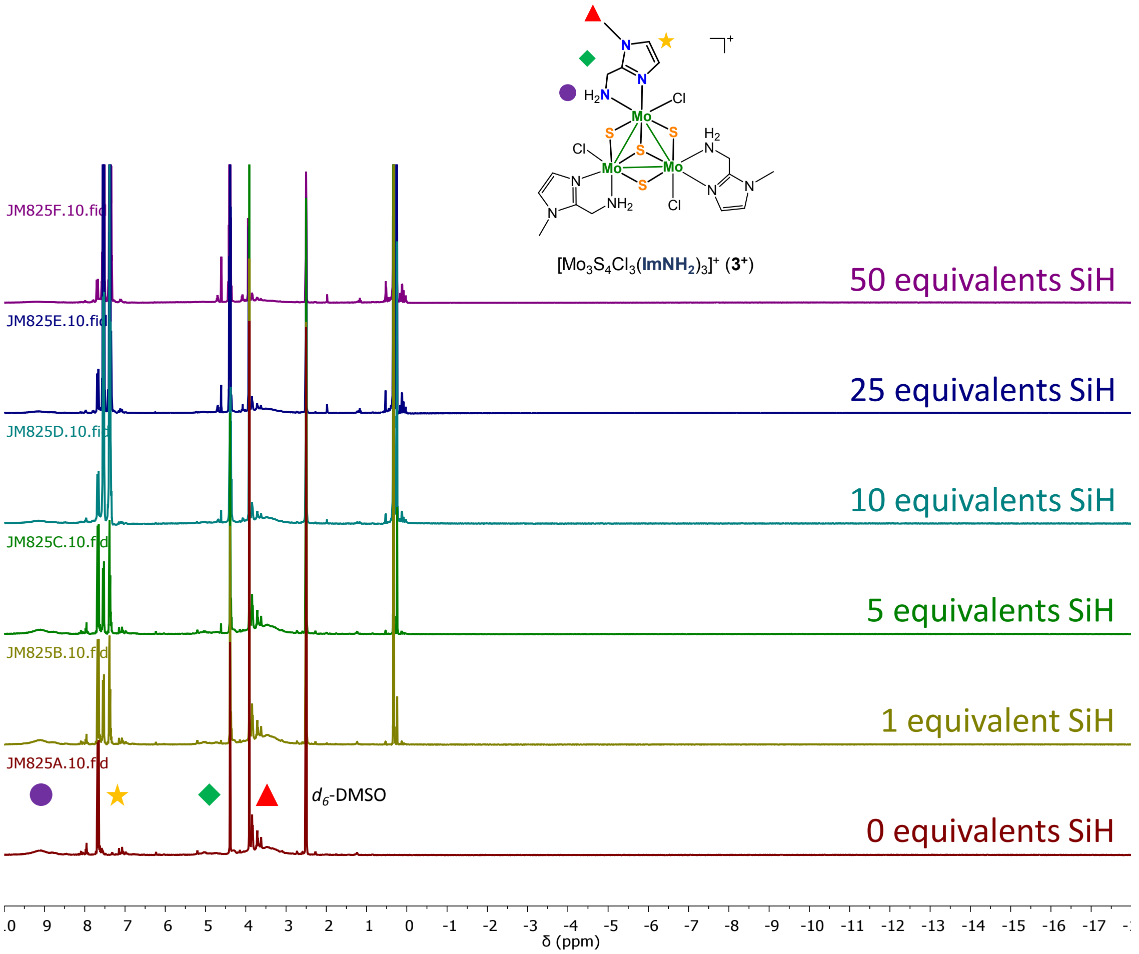


**Figure S11.** $^1$H-NMR (300 MHz, DMSO-$d_6$) spectra of the **3**$^+$ catalyst in the presence of 0-50 equivalents of dimethylphenylsilane. The mixture was allowed to react during 30 minutes between each silane addition.

### 5.3. Recycling experiments

Recycling experiments were performed by successive additions of dimethylphenylsilane (**1a**) to the reaction mixture. Once the hydrogen evolution was completed, the gas was released and the silane was introduced in the reaction vessel for the next run. After the eighth run, the reaction crude was concentrated under reduced pressure and the residue was rinsed five times with 5 mL of ethyl acetate and dried under vacuum. After that, the solid sample was analyzed by ESI-MS spectrometry and $^1$H-NMR spectroscopy to confirm the integrity of the cluster catalyst as can be seen in Figure S12. Notice that the intensity of the $^1$H-NMR signals is fairly the same before and after the catalytic process, so we assume that catalyst concentrations are almost similar.

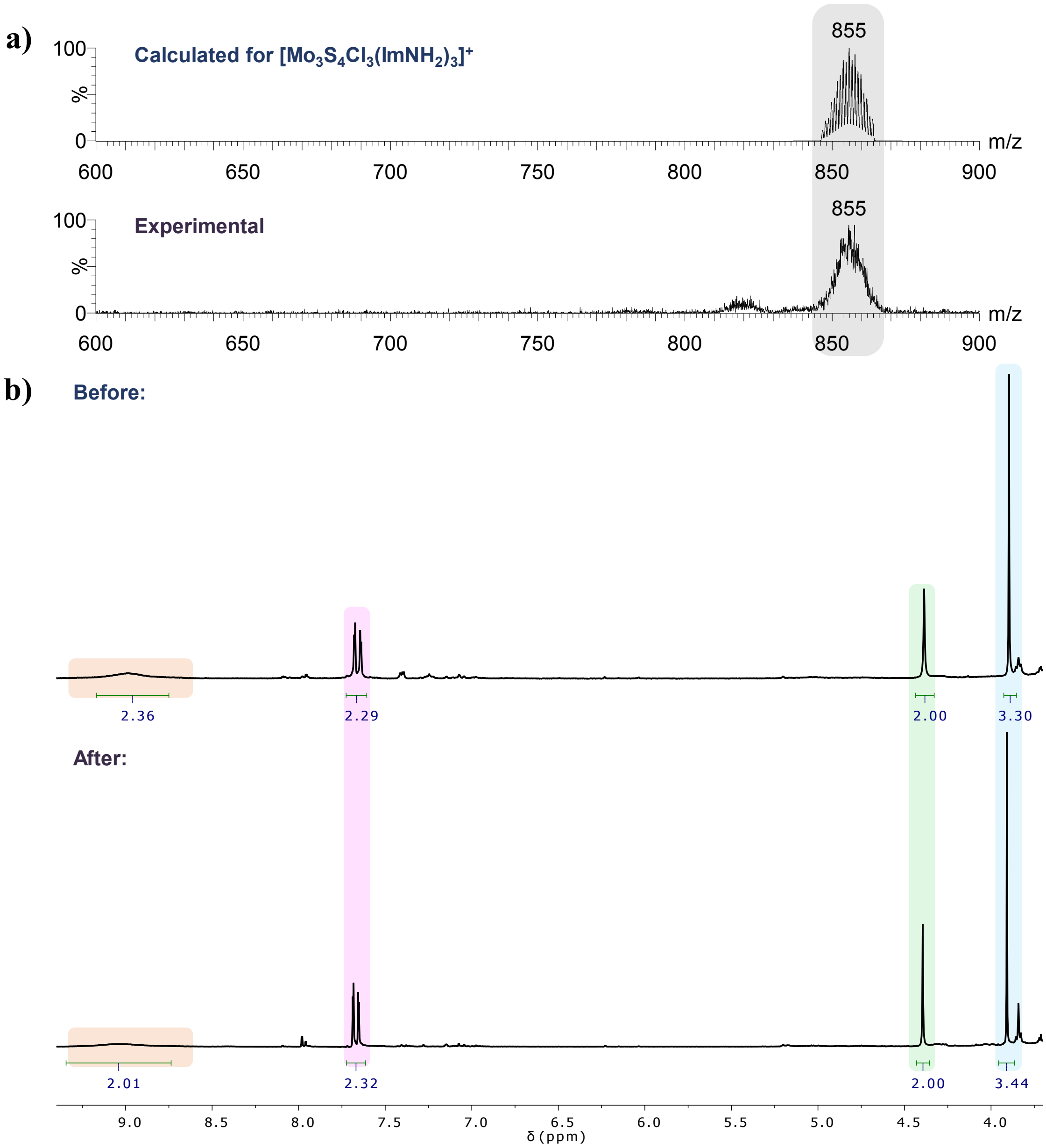


**Figure S12.** ESI-MS spectrum of catalyst **3**$^+$ after recycling experiments. Spectrum registered at 20 V in $CH_3CN$ (a). $^1$H-NMR (300 MHz, DMSO-$d_6$) spectra of catalyst **3**$^+$ before and after recycling experiments (b).

### 5.4. Mechanistic experiments. Sulfur-centered reactivity

Sulfur-centered reactivity was assessed by addition of three equivalents of dimethyl acetylenedicarboxylate to a methanolic solution of **3**$^+$ complex. Then, it was followed the experimental procedure described in Section 3 of the Supporting Information. After 150 min, the reaction mixture was analyzed by GC to quantify the organic species. Cluster integrity was analyzed by ESI mass as shown in Figure S13.

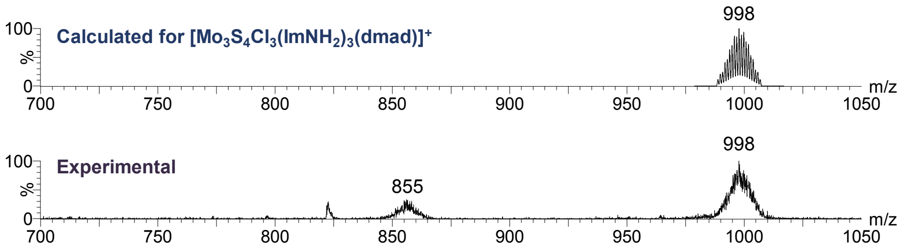


**Figure S13.** ESI-MS spectrum of catalyst **3**$^+$ after silane methanolysis. Spectrum registered at 20 V in $CH_3CN$.

## 6. Synthesis of deuterated dimethylphenylsilane (1b)

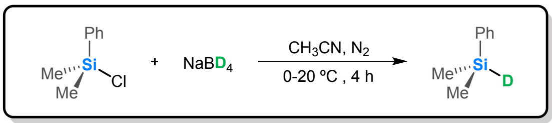


The synthesis of this compound was adapted from the literature.[12] In a 50 mL round bottom flask under nitrogen atmosphere, sodium borodeuteride (1.1030 g, 23.7 mmol). was mixed with dry $CH_3CN$ (20 mL). Then, chlorodimethylphenylsilane (2.0504 g, 12.0 mmol) was added dropwise over 15 minutes at 0 ºC and the mixture was stirred at this temperature for 30 minutes. Then, it was warmed to room temperature and stirred for 3 hours. After completing the reaction time, the mixture was quenched with dropwise addition of 10 mL of $NH_4Cl$ (sat. aqueous solution). The aqueous layer was then extracted with $CH_3CN$ (2×10 mL), and the combined organic phase was dried over anhydrous $Na_2SO_4$ and extracted with pentane (3×10 mL). The combined pentane phase was concentrated under reduced pressure and the residue was purified by column chromatography on silica gel (Pentane) to give the title compound **1b** as a colorless liquid. (1.5001 g, 91%). **$^1$H-NMR** (400 MHz, $CD_2Cl_2$): δ = 7.59-7.55 (m, 2H), 7.41-7.35 (m, 3H), 0.37 (s, 6H).

## 7. Hydrogen detection by $^1$H-NMR spectroscopy

The $^1$H-NMR spectra shown in Figure S14 were performed in high pressure NMR tube. In the case of the experiments in Figures 14a and 14c, the tube was charged with catalyst **3**$^+$ (7.1 mg, 0.008 mmol), silane **1a** or **1b** (20μL, 0.160 mmol) and $CH_3OH$ (20μL, 0.160 mmol). Then, the mixture was dissolved in 0.5 mL of DMSO-$d_6$. In the case of the experiment in Figure 14b, the tube was charged with catalyst **3**$^+$ (7.1 mg, 0.008 mmol) and silane **1b** (20μL, 0.160 mmol). Then, the mixture was dissolved in 0.5 mL of $CD_3OD$. We have not been able to obtain suitable $^2$H-NMR spectra due to intensity or overlapping issues.

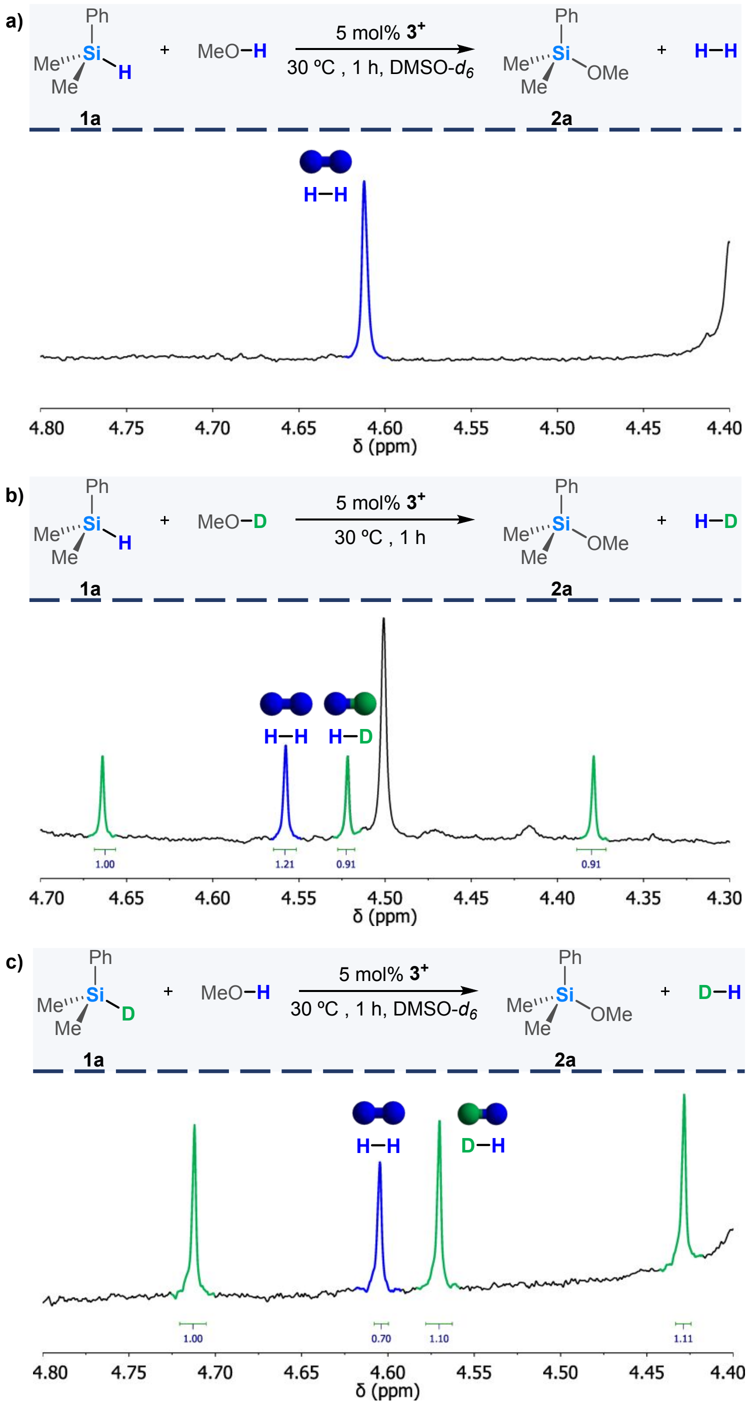


**Figure S14**. $^{1}$H-NMR (300 MHz, DMSO-$d_6$) spectra of $H_2$ gas produced inside Young's tube using a silane/methanol/catalyst ratio of 1/1/0.05 after 1h (a). $^{1}$H-NMR (300 MHz, $CD_3OD$) spectra of HD/$H_2$ gas mixture produced inside Young's tube using a silane/catalyst ratio of 1/0.05 after 1h (b). $^{1}$H-NMR (300 MHz, DMSO-$d_6$) spectra of HD/$H_2$ gas mixture produced inside Young's tube using a silane/methanol/catalyst ratio of 1/1/0.05 after 1h (c).

## 8. Kinetic Isotope Effect

Hydrogen evolution was determined by constant monitoring of the sample using a pressure transducer (Man on the Moon series X104 kit) as it is observed in Figure S15. These reactions were carried out modifying the silane ($PhMe_2SiH$ or $PhMe_2SiD$) and the solvent ($CH_3OH$ or

CD$_3$OD). The rate constants shown in this figure were calculated by fitting the kinetic data to a first-order equation.

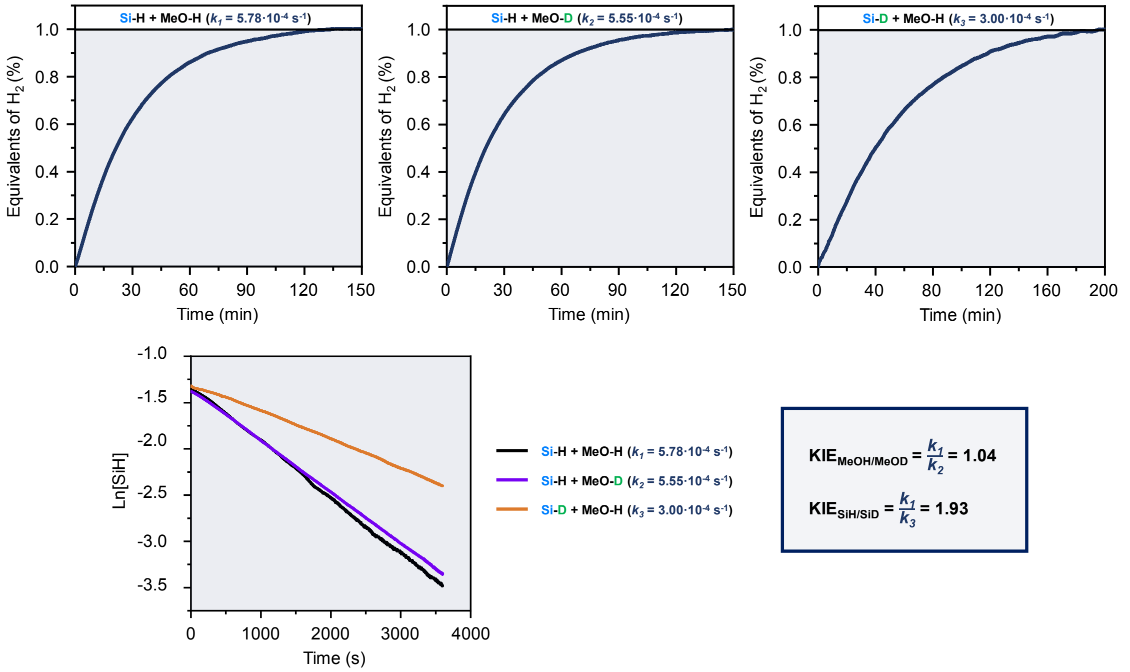


**Figure S15.** Reaction profiles (equivalents of $H_2$ *vs* time) and their reaction rate-constants. Reaction conditions: Silane (0.5 mmol), catalyst **3**$^+$ (1 mol%), T (ºC), solvent (2 mL), 150-200 min.

## 9. Computational details

Density functional theory (DFT) calculations were performed with Gaussian 16 (Revision C.02).[13] Geometry optimizations were carried out at the UBP86-D3/BS1 and UPBE0-D3/BS1 level (using Gaussian 16),[14–16] where Mo and S atoms were described using the SDD relativistic ECP and associated basis set,[17] added polarization functions for the latter ($\zeta$ = 0.503),[18] and the remaining atoms were described with the 6-31G(d,p) basis set.[19,20] Solvent effects (methanol, $\varepsilon$ = 32.613) were included self-consistently in these optimizations through the PCM method.[21,22] Note that Grimme's D3 (zero damping) dispersion corrections were implemented during the optimizations. We used the unrestricted formalism in our calculations because, based on our previous experience on spin crossing catalytic mechanisms mediated by $Mo_3S_4$ clusters (see ref. 23 of the manuscript), we investigated the possibility that the main transition structure could be triplet in their fundamental state. We found that TS1 in the triplet state lies 22.9 kcal·mol$^{-1}$ above the singlet state, and therefore we ruled out the possibility of spin-crossing processes in the present case. In addition, all stationary points found turned out to be closed-shell singlets.

All stationary points were characterized at this level of theory by analytical frequency calculations as either minima (all positive eigenvalues) or transition states (one imaginary

eigenvalue). Then, intrinsic reaction coordinate (IRC) calculations and subsequent geometry optimizations were used to confirm the minima linked by each transition state. The frequency calculations were also used to obtain the thermochemistry corrections (zero-point, thermal, and entropic energies) at the experimental temperature (303.15 K) and at the standard 1 atm pressure, on the basis of the IGRRHO (ideal gas/rigid rotor/harmonic oscillator) approach. However, these pressures and temperatures employed do not correspond to the 1 M concentration of the standard state used for species reacting in solution. Therefore, corrections (1.9355 $kcal\cdot mol^{-1}$) were applied to all Gibbs values to change the standard state to 1 M at 303.15 K. This correction has been calculated using the formula $RTln(Vm) = 1.9355\ kcal\cdot mol^{-1}$, where $Vm = 24.9\ L\cdot mol^{-1}$ and corresponds to the molar volume of an ideal gas at 1 atm and 303.15 K. Improved energetic values were obtained by performing single-point energy calculations with a larger basis set system (BS2), also including solvent effects through the PCM method. BS2 differs from BS1 in the employment of the 6-311+G(2d,2p) to describe Cl, C, N, O, and H atoms. Thus, the Gibbs energies in methanol shown in the text were obtained adding to the potential energies in methanol calculated at UBP86-D3/BS2//UBP86-D3/BS1, the Gibbs contribution at the UBP86-D3/BS1 level and the standard state correction.

The non-covalent interaction (NCI) analyses were obtained using the Multiwfn software.[23] The corresponding plots have been generated using the VMD software.[24] The three-dimensional (3D) structures shown in the text were depicted using VESTA software.[25]

### 9.1. Tentative mechanistic pathway shown in Figure 6

**Table S13**. Electronic energies ($E_{solv}$) computed with UBP86-D3/BS1(PCM) including solvent effects and dispersion corrections, and correction to Gibbs Free energies ($G_{corr}$) at the same level of theory for the reaction profile catalyzed by cluster **3**$^{+}$. Electronic energies ($E_{solv}$) also computed with UBP86-D3/BS2(PCM)//UBP86-D3/BS1(PCM) including solvent effects and dispersion corrections. Gibbs Free energies ($G_{solv}$) correspond to the sum of $E_{solv}$(BS2) and $G_{corr}$(BS1). All values are given in Hartrees. Standard state corrections (0.0031 Hartrees) are not included in this table and should be added to the last column values.

| Species | $E_{solv}$ (BS1,PCM,D3) | $G_{corr}$ (BS1,PCM,D3) | $E_{solv}$ (BS2,PCM,D3) | $G_{solv}$ (BS2,PCM,D3) |
|---|---|---|---|---|
| **Reagents and products** | | | | |
| **1a** | -601.6142 | 0.1308 | -601.7309 | -601.6001 |
| **2a** | -716.1929 | 0.1604 | -716.3500 | -716.1896 |
| **MeOH** | -115.7237 | 0.0265 | -115.7722 | -115.7457 |
| **$H_2$** | -1.1766 | -0.0018 | -1.1780 | -1.1798 |
| **Cluster species and transition states** | | | | |
| **3$^{+}$** | -2707.8576 | 0.3751 | -2708.2541 | -2707.8790 |
| **TS1'** | -3309.4849 | 0.5320 | -3309.9940 | -3309.4620 |

| | | | | |
|---|---|---|---|---|
| **I1’** | -3309.4953 | 0.5332 | -3310.0038 | -3309.4706 |
| **TS2’** | -3425.2126 | 0.5825 | -3425.7596 | -3425.1771 |
| **I2’** | -2709.0236 | 0.3909 | -2709.4216 | -2709.0307 |
| **TS3’** | -2709.0142 | 0.3868 | -2709.4130 | -2709.0262 |

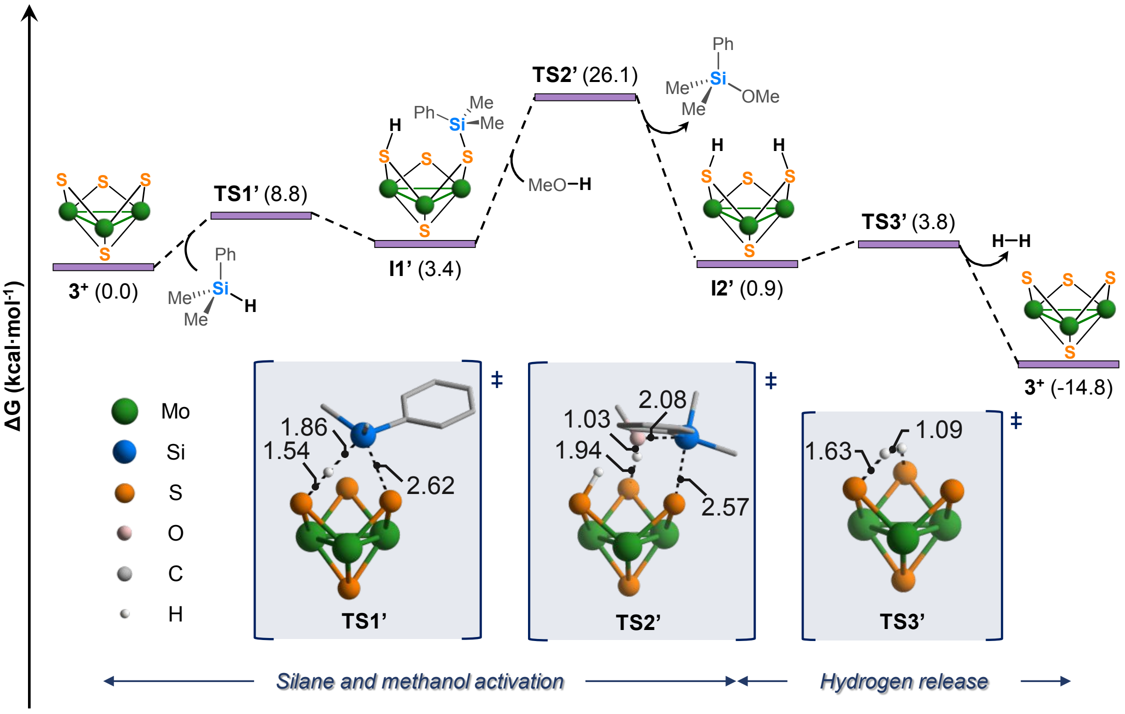


**Figure S16.** Gibbs free energy profile for the methanolysis of dimethylphenylsilane catalyzed by cluster **3**$^{+}$. Energies are given in kcal·mol$^{-1}$, quoted relative to **3**$^{+}$ + dimethylphenylsilane + methanol. Ligands and hydrogen atoms are omitted for clarity. Selected bond distances are given in Å.

**Table S14**. Electronic energies ($E_{solv}$) computed with UBP86-D3/BS1(PCM) including solvent effects and dispersion corrections, and correction to Gibbs Free energies ($G_{corr}$) at the same level of theory for methanol activation on catalyst **3**$^{+}$. Electronic energies ($E_{solv}$) also computed with UBP86-D3/BS2(PCM)//UBP86-D3/BS1(PCM) including solvent effects and dispersion corrections. Gibbs Free energies ($G_{solv}$) correspond to the sum of $E_{solv}$(BS2) and $G_{corr}$(BS1). All values are given in Hartrees. Standard state corrections (0.0031 Hartrees) are not included in this table and should be added to the last column values.

| Species | $E_{solv}$ (BS1,PCM,D3) | $G_{corr}$ (BS1,PCM,D3) | $E_{solv}$ (BS2,PCM,D3) | $G_{solv}$ (BS2,PCM,D3) |
|---|---|---|---|---|
| **TS1’’** | -2823.5413 | 0.4191 | -2823.9757 | -2823.5566 |
| **I1’’** | -2823.5422 | 0.4203 | -2823.9766 | -2823.5563 |

## 9.2. Proposed mechanistic pathway shown in Figure 7

**Table S15**. Electronic energies ($E_{solv}$) computed with UBP86-D3/BS1(PCM) including solvent effects and dispersion corrections, and correction to Gibbs Free energies ($G_{corr}$) at the same level of theory for the reaction profile catalyzed by cluster **3**$^{+}$. Electronic energies ($E_{solv}$) also computed with UBP86-D3/BS2(PCM)//UBP86-D3/BS1(PCM)including solvent effects and dispersion corrections. Gibbs Free energies ($G_{solv}$) correspond to the sum of $E_{solv}$(BS2) and $G_{corr}$(BS1). All values are given in Hartrees. Standard state corrections (0.0031 Hartrees) are not included in this table and should be added to the last column values.

| Species | $E_{solv}$ (BS1,PCM,D3) | $G_{corr}$ (BS1,PCM,D3) | $E_{solv}$ (BS2,PCM,D3) | $G_{solv}$ (BS2,PCM,D3) |
|---|---|---|---|---|
| **Reagents and products** | | | | |
| **1a** | -601.6142 | 0.1308 | -601.7309 | -601.6001 |
| **2a** | -716.1929 | 0.1604 | -716.3500 | -716.1896 |
| **MeOH** | -115.7237 | 0.0265 | -115.7722 | -115.7457 |
| **$H_2$** | -1.1766 | -0.0018 | -1.1780 | -1.1798 |
| **Cluster species and transition states** | | | | |
| **3**$^{+}$ | -2707.8576 | 0.3751 | -2708.2541 | -2707.8790 |
| **RC** | -3425.2460 | 0.5765 | -3425.7947 | -3425.2181 |
| **TS1** | -3425.2096 | 0.5775 | -3425.7567 | -3425.1793 |
| **I1** | -2709.0236 | 0.3909 | -2709.4216 | -2709.0308 |
| **TS2** | -2709.0142 | 0.3868 | -2709.4130 | -2709.0262 |

**Table S16**. Study of the charge distribution by an analysis of the Mulliken charges for **TS1** at the UBP86-D3/BS1(PCM) theory level. Highlighted hydrogen atoms (in red) are the corresponding to the hydride from silane and proton from methanol. Atom labelling corresponds to the cartesian coordinates.

| **TS1** | | | |
|---|---|---|---|
| **Atom** | **Atomic charge** | **Atom** | **Atomic charge** |
| Mo 1 | 0.04 | C 45 | 0.00 |
| Mo 2 | -0.06 | H 46 | 0.18 |
| S 3 | -0.27 | N 47 | -0.61 |
| S 4 | -0.18 | H 48 | 0.34 |
| S 5 | -0.04 | H 49 | 0.37 |
| S 6 | 0.19 | C 50 | -0.17 |
| Cl 7 | -0.34 | H 51 | 0.18 |
| Cl 8 | -0.34 | H 52 | 0.21 |
| Mo 9 | -0.02 | N 53 | -0.37 |
| Cl 10 | -0.35 | C 54 | -0.26 |
| N 11 | -0.43 | H 55 | 0.19 |
| C 12 | 0.44 | H 56 | 0.18 |
| C 13 | 0.44 | H 57 | 0.18 |
| N 14 | -0.43 | C 58 | 0.01 |
| C 15 | 0.44 | C 59 | 0.01 |

| | | | |
|---|---|---|---|
| N 16 | -0.43 | H 60 | 0.17 |
| C 17 | -0.01 | H 61 | 0.18 |
| H 18 | 0.18 | **H 62** | **-0.11** |
| N 19 | -0.61 | Si 63 | 0.67 |
| H 20 | 0.34 | C 64 | -0.56 |
| H 21 | 0.37 | H 65 | 0.16 |
| C 22 | -0.17 | H 66 | 0.17 |
| H 23 | 0.21 | H 67 | 0.15 |
| H 24 | 0.19 | C 68 | -0.58 |
| N 25 | -0.37 | H 69 | 0.15 |
| C 26 | -0.26 | H 70 | 0.16 |
| H 27 | 0.18 | H 71 | 0.15 |
| H 28 | 0.19 | C 72 | -0.09 |
| H 29 | 0.18 | C 73 | -0.12 |
| C 30 | 0.01 | C 74 | -0.12 |
| H 31 | 0.18 | C 75 | -0.10 |
| N 32 | -0.61 | H 76 | 0.12 |
| H 33 | 0.34 | C 77 | -0.11 |
| H 34 | 0.37 | H 78 | 0.12 |
| C 35 | -0.18 | C 79 | -0.13 |
| H 36 | 0.22 | H 80 | 0.11 |
| H 37 | 0.18 | H 81 | 0.12 |
| N 38 | -0.36 | H 82 | 0.12 |
| C 39 | -0.26 | O 83 | -0.50 |
| H 40 | 0.18 | **H 84** | **0.36** |
| H 41 | 0.18 | C 85 | -0.16 |
| H 42 | 0.18 | H 86 | 0.17 |
| C 43 | 0.02 | H 87 | 0.17 |
| H 44 | 0.17 | H 88 | 0.16 |

**Table S17**. Gibbs Free values calculated with different functionals. Energies are given in kcal·mol$^{-1}$, quoted relative to each cluster + dimethylphenylsilane + methanol.

| | **ΔG (kcal·mol$^{-1}$)** | |
|---|---|---|
| **Stationary Point** | **UBP86-D3** [a] | **UPBE0-D3** |
| **TS1** | 24.7 | - |
| **TS1A$_{PBE}$** | - | 34.3 |
| **IA$_{PBE}$** | - | 7.8 |
| **TS1B$_{PBE}$** | - | 6.4 |
| **I1** | 0.9 | -1.1 |
| **TS2** | 3.8 | 5.3 |
| **Products** | -14.8 | -14.0 |

[a]Values used in the main text

**Note:** In the mechanism calculated using the UPBE0-D3 functional, the proton transfer from **IA$_{PBE}$** to **I1** occurs through an apparent reduction of the G value from **IA$_{PBE}$** to **TS1B$_{PBE}$**. This unrealistic energy arises when dispersion and standard state corrections are applied to calculate relative free energies in neighboring stationary points that present similar potential energies.

### 9.3. Theoretical KIE calculations

The calculated KIEs have been obtained on the basis of Eyring theory using eq.1 and eq.2. To do so, the calculated energy barriers for H and D are obtained by performing frequency calculations for both isotopes on the reactants and transition state (**TS1**). Results are collected in Tables S18 and S19.

$$k = \frac{k_B T}{h} e^{-\Delta G/RT} \tag{1}$$

$$\frac{k_H}{k_D} = \frac{e^{-\Delta G_H/RT}}{e^{-\Delta G_D/RT}} \tag{2}$$

**Table S18**. Electronic energies ($E_{solv}$) computed with UBP86-D3/BS1(PCM) including solvent effects and dispersion corrections and, correction to Gibbs Free energies ($G_{corr}$) at the same level of theory for deuterated species. Electronic energies ($E_{solv}$) also computed with UBP86-D3/BS2(PCM)//UBP86-D3/BS1(PCM) including solvent effects and dispersion corrections. Gibbs Free energies ($G_{solv}$) correspond to the sum of $E_{solv}$(BS2) and $G_{corr}$(BS1). All values are given in Hartrees. Standard state corrections (0.0031 Hartrees) are not included in this table and should be added to the last column values.

| Species | $E_{solv}$ (BS1,PCM,D3) | $G_{corr}$ (BS1,PCM,D3) | $E_{solv}$ (BS2,PCM,D3) | $G_{solv}$ (BS2,PCM,D3) |
|---|---|---|---|---|
| | | **Deuterated species** | | |
| **$PhMe_2SiD$** | -2198.8121 | 0.3000 | -2199.0752 | -2198.7752 |
| **$CD_3OD$** | -2916.2089 | 0.5012 | -2916.6238 | -2916.1226 |
| **TS1_SiD** | -2916.1619 | 0.5015 | -2916,5755 | -2916.0740 |
| **TS1_OD** | -2199.9682 | 0.3109 | -2200.2335 | -2199.9226 |

**Table S19**. Thermodynamic data for KIE calculation.

| Species | ΔG (kcal·mol$^{-1}$) | ΔG/RT | $e^{-\Delta G/RT}$ |
|---|---|---|---|
| **TS1** | 24.7 | 41.01 | $1.54\cdot10^{-18}$ |
| **TS1_SiD** | 25.3 | 41.92 | $6.23\cdot10^{-19}$ |
| **TS1_OD** | 24.9 | 41.28 | $1.18\cdot10^{-18}$ |

When $PhMe_2SiD$ is used: $KIE_{theo} = \frac{k_H}{k_D} = 2.48$

When $CD_3OD$ is used: $KIE_{theo} = \frac{k_H}{k_D} = 1.31$

### 9.4. Comparative study of Gibbs free energy profile for clusters $1^+$, $2^+$and $3^+$

**Table S20**. Electronic energies ($E_{solv}$) computed with UBP86-D3/BS1(PCM) including solvent effects and dispersion corrections and, correction to Gibbs Free energies ($G_{corr}$) at the same level of theory for the reaction profile catalyzed by cluster **1**$^+$ and **2**$^+$. Electronic energies ($E_{solv}$) also computed with UBP86-D3/BS2(PCM)//UBP86-D3/BS1(PCM) including solvent effects and dispersion corrections. Gibbs Free energies ($G_{solv}$) correspond to the sum of $E_{solv}$(BS2) and $G_{corr}$(BS1). All values are given in Hartrees. Standard state corrections (0.0031 Hartrees) are not included in this table and should be added to the last column values.

| Species | $E_{solv}$ (BS1,PCM,D3) | $G_{corr}$ (BS1,PCM,D3) | $E_{solv}$ (BS2,PCM,D3) | $G_{solv}$ (BS2,PCM,D3) |
|---|---|---|---|---|
| **Cluster species and transition states (Catalyst 1$^+$)** | | | | |
| **1$^+$** | -2198.8121 | 0.3000 | -2199.0752 | -2198.7752 |
| **TS1** | -2916.1619 | 0.5015 | -2916,5755 | -2916.0740 |
| **I1** | -2199.9776 | 0.3146 | -2200.2422 | -2199.9277 |
| **TS2** | -2199.9682 | 0.3109 | -2200.2335 | -2199.9226 |
| **Cluster species and transition states (Catalyst 2$^+$)** | | | | |
| **2$^+$** | -2434.6599 | 0.4558 | -2434.9807 | -2434.5250 |
| **TS1** | -3152.0049 | 0.6556 | -3152.4764 | -3151.8208 |
| **I1** | -2435.8243 | 0.4713 | -2436.1468 | -2435.6755 |
| **TS2** | -2435.8160 | 0.4675 | -2436.1391 | -2435.6716 |

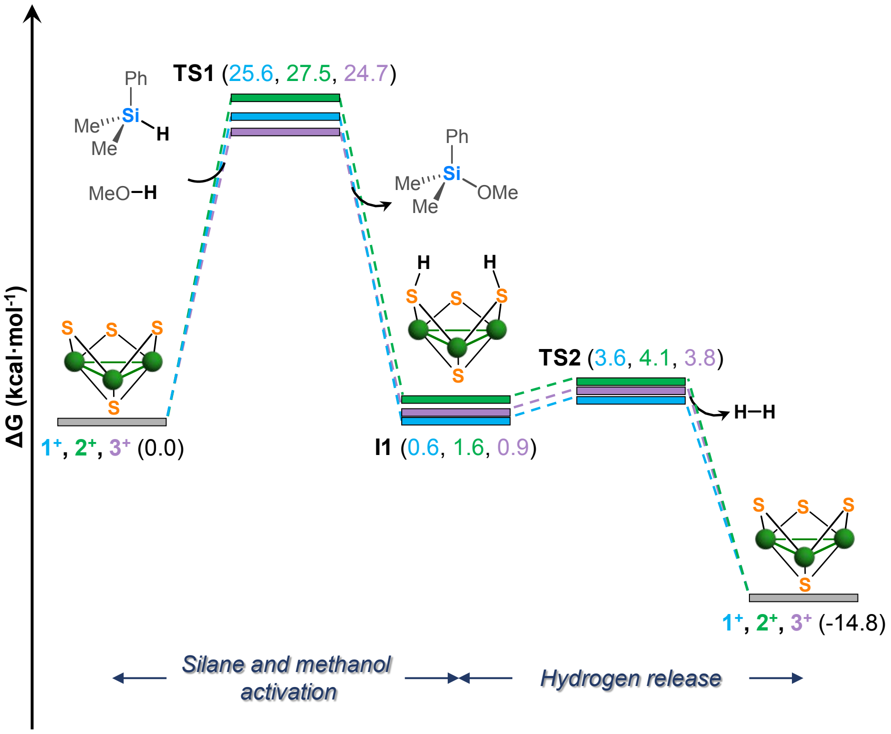


**Figure S17.** Free Gibbs energy profile for the methanolysis of dimethylphenylsilane catalyzed by clusters **1**+, **2**+ and **3**+. Energies are given in kcal·mol-1, quoted relative to each cluster + dimethylphenylsilane + methanol. Ligands and hydrogen atoms are omitted for clarity. Selected bond distances are given in Å.

### 9.5. Non-covalent interaction (NCI) analysis

Non-covalent interaction (NCI) analysis correlates the reduced density gradient (RDG) with a function of the electron density (ρ). The RDG can be expressed as a function of the gradient of ρ according to the eq.3 This correlation allows us to distinguish the different NCIs of a system depending on the region where they appear. At these regions, the RDG approaches zero, indicating the presence of an inflection point between two density maxima.

$$s(r) = \frac{1}{2(3\pi^2)^{\frac{1}{3}}} \frac{|\nabla\rho(r)|}{\rho(r)^{\frac{4}{3}}} \qquad (3)$$

The strength of the NCIs depends on the electron density at these points. The attractive or repulsive character of the interaction can be assessed by the study of the curvature of the electron density ($\nabla^2\rho$), which is dominated by attractive electron-nucleus interactions. Therefore, the local curvatures in the diagonalized Hessian of the electronic density matrix (containing the second-order partial derivatives) are good indicators, specifically the second eigenvalue ($\lambda_2$). Thus, $\lambda_2$ is negative for attractive interactions and *vice versa*. Thus, a plot of the

RDG against sign($\lambda_2$)ρ displays the NCIs as peaks of the electron density where RDG is zero.[26,27]

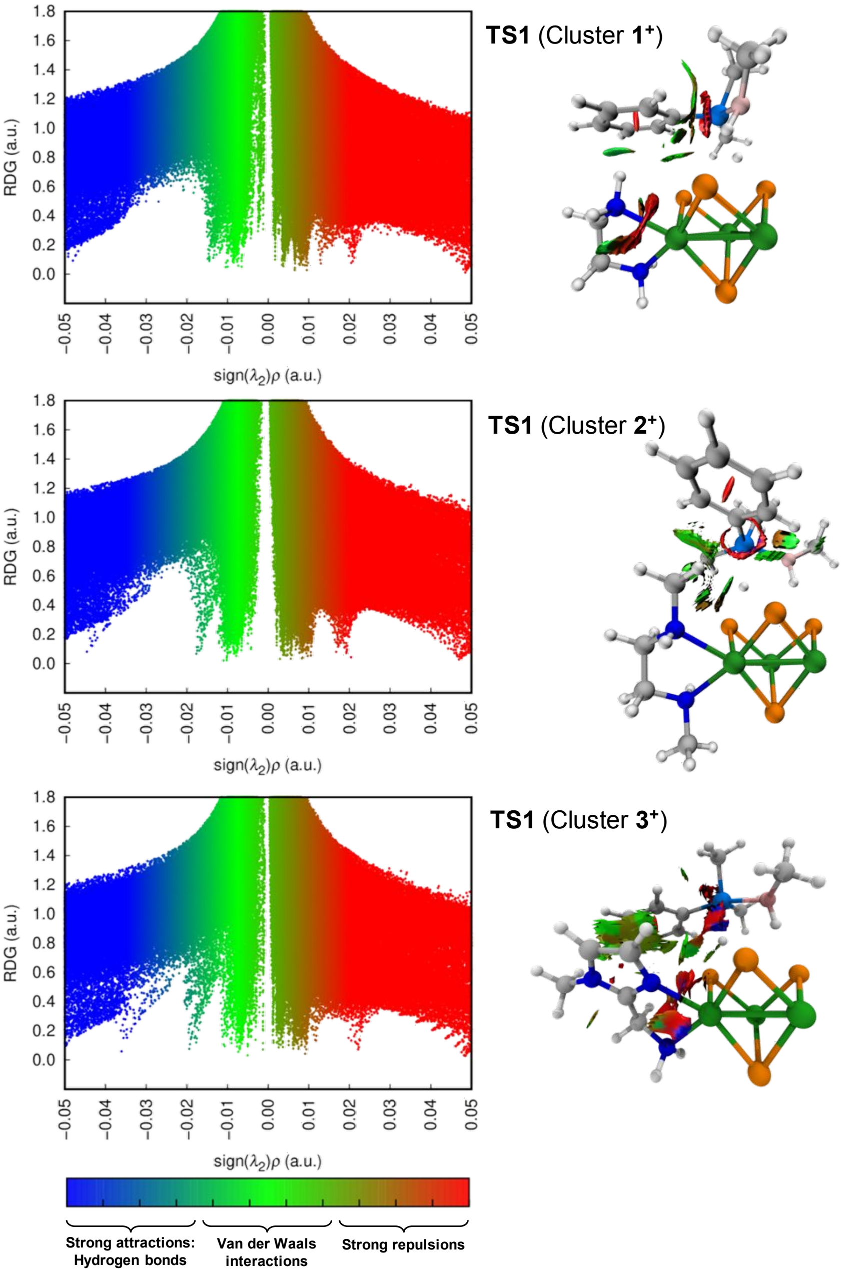


**Figure S18.** NCI analysis performed on TS1 of complexes $\mathbf{1}^+$, $\mathbf{2}^+$ and $\mathbf{3}^+$ by plotting the reduced density gradient (RDG) against the sign of the second eigenvalue of the Hessian matrix multiplied by the electron density (sign($\lambda_2$)ρ). The most relevant NCIs are displayed as isosurfaces (isovalue=0.5 a.u.).

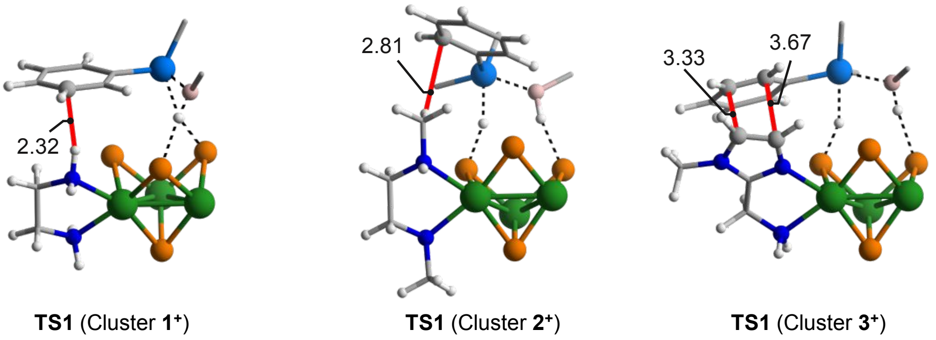


**Figure S19.** Distance analysis between the aromatic ring of the silane **1a** and the organic ligand of the cluster in the **TS1** for catalysts **1**$^+$, **2**$^+$ and **3**$^+$. Some ligands are omitted for clarity. Selected bond distances are given in Å.

### 9.6. 3D views of the computed 3$^+$ cluster species

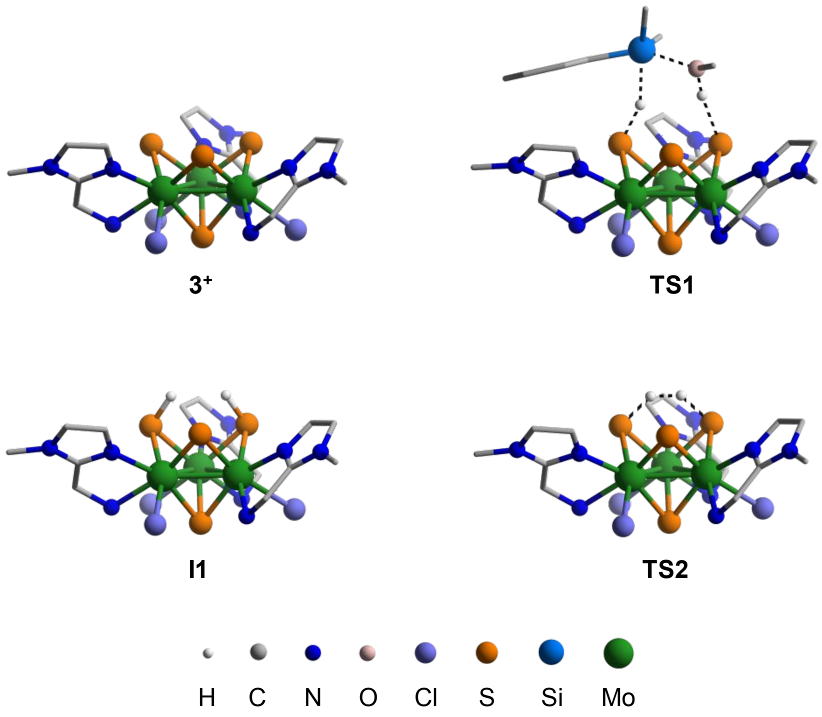


**Figure S20.** Optimized structures of intermediates and transition states for the Gibbs energy profile represented in Figure 7. Selected distances are given in Å.

## 9.7. Cartesian coordinates of the optimized structures

**Optimized with UBP86-D3**

### Stationary point: Dimethylphenylsilane (1a)

| | x | y | z |
|---|---|---|---|
| C | 5.8101 | 1.9807 | -2.3354 |
| C | 5.7717 | 0.6124 | -2.6932 |
| C | 6.1331 | -0.3384 | -1.7071 |
| C | 6.5171 | 0.0600 | -0.4162 |
| C | 6.5476 | 1.4260 | -0.0831 |
| C | 6.1932 | 2.3867 | -1.0450 |
| Si | 5.2534 | 0.0519 | -4.4249 |
| H | 5.0008 | 1.3022 | -5.2260 |
| C | 3.6683 | -0.9818 | -4.3332 |
| C | 6.6409 | -0.9585 | -5.2263 |
| H | 6.1163 | -1.4088 | -1.9479 |
| H | 6.7928 | -0.6929 | 0.3303 |
| H | 6.8469 | 1.7393 | 0.9228 |
| H | 6.2158 | 3.4520 | -0.7907 |
| H | 5.5374 | 2.7427 | -3.0756 |
| H | 3.3620 | -1.3253 | -5.3361 |
| H | 3.8320 | -1.8737 | -3.7035 |
| H | 2.8371 | -0.4041 | -3.8955 |
| H | 6.3463 | -1.3075 | -6.2308 |
| H | 7.5656 | -0.3653 | -5.3225 |
| H | 6.8688 | -1.8469 | -4.6118 |

### Stationary point: MeOH

| | x | y | z |
|---|---|---|---|
| C | -1.2011 | -0.4943 | 0.0123 |
| H | -0.8274 | -1.5317 | 0.0242 |
| H | -0.8432 | -0.0151 | -0.9228 |
| H | -2.3097 | -0.5330 | -0.0244 |
| O | -0.7027 | 0.1430 | 1.1932 |
| H | -1.0351 | 1.0596 | 1.1787 |

### Stationary point: Methoxydimethylphenylsilane (2a)

| | x | y | z |
|---|---|---|---|
| C | -8.0761 | -3.3444 | 0.6047 |
| C | -8.0397 | -3.6240 | 1.9816 |
| C | -6.8150 | -3.9296 | 2.6001 |
| C | -5.6323 | -3.9511 | 1.8420 |

| | | | |
|---|---|---|---|
| C | -5.6429 | -3.6751 | 0.4533 |
| C | -6.8898 | -3.3735 | -0.1478 |
| Si | -4.0430 | -3.6512 | -0.5539 |
| C | -2.6793 | -4.6195 | 0.3046 |
| O | -3.4617 | -2.0580 | -0.6358 |
| C | -4.2871 | -1.0252 | -1.1920 |
| C | -4.3738 | -4.3115 | -2.2909 |
| H | -6.9403 | -3.1609 | -1.2225 |
| H | -9.0282 | -3.1072 | 0.1178 |
| H | -8.9630 | -3.6056 | 2.5704 |
| H | -6.7824 | -4.1514 | 3.6723 |
| H | -4.6867 | -4.1909 | 2.3425 |
| H | -4.4921 | -1.1954 | -2.2676 |
| H | -5.2581 | -0.9447 | -0.6641 |
| H | -3.7542 | -0.0648 | -1.0894 |
| H | -3.4458 | -4.3016 | -2.8876 |
| H | -4.7419 | -5.3508 | -2.2447 |
| H | -5.1284 | -3.7124 | -2.8285 |
| H | -1.7599 | -4.6084 | -0.3046 |
| H | -2.4383 | -4.1961 | 1.2939 |
| H | -2.9819 | -5.6709 | 0.4469 |

**Stationary point: $H_2$**

| | x | y | z |
|---|---|---|---|
| H | 0.0000 | 0.0000 | -0.0254 |
| H | 0.0000 | 0.0000 | 0.7254 |

**Stationary point: Cluster $3^+$**

| | x | y | z |
|---|---|---|---|
| C | 0.3566 | 1.7164 | 0.3260 |
| N | 0.7946 | 0.4047 | 0.2643 |
| C | -0.0234 | -0.2512 | -0.5777 |
| N | -0.9714 | 0.5967 | -1.0598 |
| C | -0.7410 | 1.8486 | -0.4994 |
| Mo | 2.3130 | -0.8199 | 1.2754 |
| N | 0.9609 | -2.3104 | 0.2032 |
| C | 0.1932 | -1.6921 | -0.9184 |
| C | -2.0252 | 0.2534 | -2.0211 |
| Mo | 4.4062 | -0.7461 | 3.0869 |
| N | 3.1148 | -0.7612 | 4.9666 |
| C | 3.2915 | 0.4663 | 5.7983 |
| C | 4.6860 | 0.9635 | 5.5793 |
| N | 5.3646 | 0.6827 | 4.4526 |
| C | 6.5888 | 1.3230 | 4.5385 |

| | | | |
|---|---|---|---|
| C | 6.6411 | 2.0040 | 5.7371 |
| N | 5.4342 | 1.7641 | 6.3852 |
| C | 5.0265 | 2.2952 | 7.6909 |
| Mo | 4.7039 | -2.0872 | 0.6836 |
| N | 6.0741 | -3.7300 | 1.4738 |
| C | 7.4210 | -3.7148 | 0.8293 |
| C | 7.2522 | -3.1762 | -0.5565 |
| N | 6.2261 | -2.3681 | -0.8765 |
| C | 6.3831 | -2.0084 | -2.2039 |
| C | 7.5279 | -2.6090 | -2.6856 |
| N | 8.0630 | -3.3474 | -1.6354 |
| C | 9.3013 | -4.1332 | -1.6687 |
| S | 6.1939 | -0.5477 | 1.5946 |
| S | 3.1475 | 1.0744 | 2.3583 |
| S | 3.2116 | -2.6909 | 2.4297 |
| Cl | 5.7606 | -2.3790 | 4.5222 |
| S | 3.5158 | -0.6339 | -0.7102 |
| Cl | 3.6914 | -4.1045 | -0.5276 |
| Cl | 0.4229 | -0.9944 | 2.9950 |
| H | 5.6686 | -1.3653 | -2.7108 |
| H | 6.1605 | -3.6125 | 2.4998 |
| H | 5.6032 | -4.6272 | 1.2988 |
| H | 8.0696 | -3.0413 | 1.4179 |
| H | 7.8856 | -4.7144 | 0.8320 |
| H | 9.6239 | -4.2340 | -2.7127 |
| H | 9.1187 | -5.1351 | -1.2525 |
| H | 10.0890 | -3.6290 | -1.0885 |
| H | 8.0017 | -2.5879 | -3.6634 |
| H | 2.1252 | -0.8653 | 4.6766 |
| H | 3.3733 | -1.5984 | 5.5045 |
| H | 2.5646 | 1.2208 | 5.4472 |
| H | 3.0836 | 0.2713 | 6.8632 |
| H | 4.7194 | 1.4700 | 8.3509 |
| H | 4.1934 | 3.0040 | 7.5703 |
| H | 5.8826 | 2.8133 | 8.1412 |
| H | 7.3341 | 1.2407 | 3.7519 |
| H | 7.4144 | 2.6210 | 6.1869 |
| H | 1.5511 | -3.0877 | -0.1454 |
| H | 0.3185 | -2.6955 | 0.9073 |
| H | -0.7561 | -2.2216 | -1.1031 |
| H | 0.8069 | -1.7736 | -1.8336 |
| H | -1.5835 | 0.0115 | -2.9997 |
| H | -2.6960 | 1.1152 | -2.1277 |
| H | -2.6034 | -0.6069 | -1.6519 |
| H | 0.8469 | 2.4544 | 0.9554 |
| H | -1.3750 | 2.7000 | -0.7323 |

## Stepwise mechanism

**Stationary point: TS1’**

| | x | y | z |
|---|---|---|---|
| Mo | 0.2151 | 1.5493 | -0.9514 |
| Mo | 1.1725 | -0.9644 | -0.8776 |
| S | -0.4217 | -1.9304 | -2.3626 |
| S | 1.7132 | 0.6369 | -2.4991 |
| S | -1.5711 | 1.1223 | -2.4587 |
| S | -0.0288 | 0.0721 | 0.9065 |
| Cl | 1.1519 | -3.0600 | 0.5917 |
| Cl | 2.0260 | 2.6348 | 0.4637 |
| Mo | -1.5485 | -0.5466 | -0.8118 |
| Cl | -3.3190 | 0.6022 | 0.6249 |
| N | -3.3771 | -1.4163 | -1.6813 |
| C | -3.7268 | -2.6290 | -1.2193 |
| C | -0.4943 | 4.4152 | -1.6692 |
| N | 0.4125 | 3.4751 | -1.9877 |
| C | 4.0274 | -1.8205 | -1.4574 |
| N | 2.7783 | -2.1135 | -1.8550 |
| C | 2.8695 | -3.1326 | -2.7862 |
| H | 1.9861 | -3.5591 | -3.2541 |
| N | 3.0467 | -0.4867 | 0.2945 |
| H | 2.9835 | 0.5034 | 0.5977 |
| H | 3.0429 | -1.0765 | 1.1364 |
| C | 4.2949 | -0.7069 | -0.4943 |
| H | 4.5074 | 0.2246 | -1.0501 |
| H | 5.1600 | -0.9170 | 0.1562 |
| N | 4.9214 | -2.6181 | -2.1037 |
| C | 6.3803 | -2.5730 | -1.9574 |
| H | 6.8130 | -3.4297 | -2.4896 |
| H | 6.6516 | -2.6386 | -0.8932 |
| H | 6.7775 | -1.6394 | -2.3840 |
| C | 4.2021 | -3.4525 | -2.9529 |
| H | 4.6987 | -4.1891 | -3.5790 |
| N | -1.1691 | 2.9943 | 0.1485 |
| H | -1.9829 | 2.4580 | 0.5065 |
| H | -0.6547 | 3.3662 | 0.9567 |
| C | -1.6242 | 4.1075 | -0.7374 |
| H | -2.5014 | 3.7450 | -1.3037 |
| H | -1.9380 | 4.9913 | -0.1576 |
| N | -0.1760 | 5.5897 | -2.2810 |
| C | -0.9723 | 6.8189 | -2.2771 |
| H | -1.4004 | 6.9851 | -1.2783 |
| H | -1.7834 | 6.7483 | -3.0188 |

| | | | |
|---|---|---|---|
| H | -0.3183 | 7.6643 | -2.5287 |
| C | 1.3435 | 4.0617 | -2.8234 |
| H | 2.1767 | 3.4992 | -3.2328 |
| C | 0.9829 | 5.3772 | -3.0205 |
| H | 1.4182 | 6.1641 | -3.6290 |
| N | -2.0443 | -2.3538 | 0.4767 |
| H | -1.1509 | -2.7735 | 0.7952 |
| H | -2.5492 | -2.0109 | 1.3041 |
| C | -2.8619 | -3.3692 | -0.2485 |
| H | -3.4543 | -3.9933 | 0.4413 |
| H | -2.1674 | -4.0363 | -0.7911 |
| N | -4.9013 | -3.0217 | -1.7828 |
| C | -5.5968 | -4.2884 | -1.5289 |
| H | -5.8476 | -4.3729 | -0.4605 |
| H | -4.9627 | -5.1360 | -1.8295 |
| H | -6.5231 | -4.3032 | -2.1171 |
| C | -5.3156 | -2.0072 | -2.6403 |
| C | -4.3629 | -1.0119 | -2.5642 |
| H | -4.3183 | -0.0492 | -3.0652 |
| H | -6.2374 | -2.0871 | -3.2102 |
| H | -0.3304 | -0.8509 | -3.4514 |
| Si | -0.6081 | 0.4106 | -4.7934 |
| C | -2.3351 | -0.0833 | -5.4042 |
| H | -2.6597 | -1.0252 | -4.9302 |
| H | -3.0825 | 0.6929 | -5.1721 |
| H | -2.3082 | -0.2320 | -6.4981 |
| C | 0.6551 | -0.6811 | -5.7292 |
| H | 0.6462 | -0.4218 | -6.8036 |
| H | 1.6744 | -0.5508 | -5.3323 |
| H | 0.3777 | -1.7434 | -5.6219 |
| C | -0.2339 | 2.2142 | -5.2358 |
| C | 0.9837 | 2.5595 | -5.8683 |
| C | -1.2009 | 3.2347 | -5.0619 |
| C | 1.2203 | 3.8659 | -6.3291 |
| H | 1.7580 | 1.7986 | -6.0148 |
| C | -0.9676 | 4.5421 | -5.5158 |
| H | -2.1523 | 3.0073 | -4.5676 |
| C | 0.2431 | 4.8592 | -6.1571 |
| H | 2.1685 | 4.1071 | -6.8213 |
| H | -1.7313 | 5.3140 | -5.3720 |
| H | 0.4249 | 5.8784 | -6.5135 |

**Stationary point: I1'**

| | | x | y | z |
|---|---|---|---|---|
| Mo | 0.6238 | 1.1119 | 0.5411 | |

| | | | |
|---|---|---|---|
| Mo | 1.3630 | -1.4360 | 0.9232 |
| S | -0.1289 | -2.5001 | -0.6789 |
| S | 2.1913 | -0.0204 | -0.7723 |
| S | -1.2109 | 0.7965 | -0.9983 |
| S | 0.0913 | -0.1728 | 2.4894 |
| Cl | 1.0779 | -3.3990 | 2.5430 |
| Cl | 2.3408 | 2.2194 | 2.0222 |
| Mo | -1.2949 | -0.8965 | 0.7048 |
| Cl | -3.0865 | 0.5233 | 1.8725 |
| N | -3.1192 | -1.7546 | -0.1826 |
| C | -3.5412 | -2.9101 | 0.3582 |
| C | 0.2068 | 3.9440 | -0.4938 |
| N | 1.0373 | 2.9025 | -0.6783 |
| C | 4.2064 | -2.4961 | 0.7369 |
| N | 2.9906 | -2.7641 | 0.2333 |
| C | 3.1130 | -3.8908 | -0.5598 |
| H | 2.2571 | -4.3225 | -1.0722 |
| N | 3.1377 | -0.9468 | 2.2398 |
| H | 3.1107 | 0.0663 | 2.4580 |
| H | 3.0192 | -1.4649 | 3.1196 |
| C | 4.4402 | -1.2970 | 1.6001 |
| H | 4.7437 | -0.4387 | 0.9734 |
| H | 5.2328 | -1.4683 | 2.3474 |
| N | 5.1095 | -3.4129 | 0.2917 |
| C | 6.5435 | -3.4324 | 0.5989 |
| H | 6.9684 | -4.3749 | 0.2303 |
| H | 6.6941 | -3.3748 | 1.6872 |
| H | 7.0508 | -2.5867 | 0.1098 |
| C | 4.4310 | -4.3009 | -0.5365 |
| H | 4.9414 | -5.1317 | -1.0166 |
| N | -0.7361 | 2.7550 | 1.3837 |
| H | -1.6265 | 2.3103 | 1.6791 |
| H | -0.2784 | 3.1302 | 2.2234 |
| C | -0.9910 | 3.8473 | 0.3975 |
| H | -1.8735 | 3.5648 | -0.2057 |
| H | -1.2117 | 4.8065 | 0.8942 |
| N | 0.6204 | 5.0056 | -1.2372 |
| C | -0.0156 | 6.3256 | -1.2787 |
| H | 0.1944 | 6.8795 | -0.3503 |
| H | -1.0996 | 6.2073 | -1.4142 |
| H | 0.3837 | 6.8793 | -2.1379 |
| C | 2.0218 | 3.3132 | -1.5610 |
| H | 2.8235 | 2.6479 | -1.8701 |
| C | 1.7694 | 4.6213 | -1.9187 |
| H | 2.2800 | 5.3005 | -2.5955 |
| N | -1.9738 | -2.5089 | 2.1275 |

| | | | |
|---|---|---|---|
| H | -1.1307 | -2.9220 | 2.5713 |
| H | -2.5411 | -2.0625 | 2.8606 |
| C | -2.7585 | -3.5780 | 1.4428 |
| H | -3.4082 | -4.1275 | 2.1443 |
| H | -2.0404 | -4.3003 | 1.0138 |
| N | -4.7134 | -3.2940 | -0.2160 |
| C | -5.4683 | -4.5131 | 0.0942 |
| H | -5.6888 | -4.5546 | 1.1715 |
| H | -4.8918 | -5.4030 | -0.2004 |
| H | -6.4134 | -4.4908 | -0.4634 |
| C | -5.0518 | -2.3313 | -1.1622 |
| C | -4.0580 | -1.3751 | -1.1251 |
| H | -3.9610 | -0.4487 | -1.6821 |
| H | -5.9569 | -2.4154 | -1.7578 |
| H | 0.1738 | -1.7841 | -1.8333 |
| Si | -0.8248 | 0.7983 | -3.2079 |
| C | -2.1718 | -0.3080 | -3.9331 |
| H | -2.1539 | -1.3133 | -3.4805 |
| H | -3.1729 | 0.1284 | -3.7860 |
| H | -1.9973 | -0.4070 | -5.0188 |
| C | 0.8683 | 0.3365 | -3.8871 |
| H | 0.8449 | 0.4911 | -4.9821 |
| H | 1.6593 | 0.9652 | -3.4515 |
| H | 1.1154 | -0.7167 | -3.6849 |
| C | -1.1699 | 2.6284 | -3.5337 |
| C | -0.2516 | 3.4352 | -4.2428 |
| C | -2.3679 | 3.2299 | -3.0715 |
| C | -0.5222 | 4.7922 | -4.4925 |
| H | 0.6926 | 3.0106 | -4.5989 |
| C | -2.6419 | 4.5840 | -3.3173 |
| H | -3.0951 | 2.6331 | -2.5073 |
| C | -1.7177 | 5.3673 | -4.0336 |
| H | 0.2061 | 5.4011 | -5.0380 |
| H | -3.5733 | 5.0292 | -2.9522 |
| H | -1.9266 | 6.4250 | -4.2238 |

**Stationary point: TS2'**

| | | x | y | z |
|---|---|---|---|---|
| Mo | 0.3126 | 1.6391 | -0.9457 | |
| Mo | 1.2545 | -0.9128 | -0.7398 | |
| S | -0.2188 | -2.0095 | -2.2397 | |
| S | 2.0258 | 0.6834 | -2.3242 | |
| S | -1.4725 | 1.3016 | -2.5580 | |
| S | -0.0085 | 0.2271 | 0.9358 | |
| Cl | 1.1753 | -2.8918 | 0.8784 | |

| | | | |
|---|---|---|---|
| Cl | 2.1645 | 2.6966 | 0.5020 |
| Mo | -1.4093 | -0.4500 | -0.8604 |
| Cl | -3.2709 | 0.7793 | 0.3910 |
| N | -3.1774 | -1.3701 | -1.8139 |
| C | -3.5733 | -2.5413 | -1.2887 |
| C | -0.2868 | 4.5447 | -1.6073 |
| N | 0.5663 | 3.5728 | -1.9758 |
| C | 4.1232 | -1.8474 | -1.1328 |
| N | 2.8917 | -2.1248 | -1.5933 |
| C | 3.0170 | -3.1478 | -2.5167 |
| H | 2.1519 | -3.5614 | -3.0283 |
| N | 3.0914 | -0.4037 | 0.5054 |
| H | 3.0314 | 0.6102 | 0.7268 |
| H | 3.0279 | -0.9280 | 1.3863 |
| C | 4.3715 | -0.7074 | -0.1964 |
| H | 4.6531 | 0.1873 | -0.7816 |
| H | 5.1893 | -0.9202 | 0.5118 |
| N | 5.0375 | -2.6635 | -1.7281 |
| C | 6.4900 | -2.6153 | -1.5318 |
| H | 6.9358 | -3.5113 | -1.9823 |
| H | 6.7205 | -2.6024 | -0.4564 |
| H | 6.9074 | -1.7162 | -2.0110 |
| C | 4.3518 | -3.4890 | -2.6138 |
| H | 4.8696 | -4.2341 | -3.2119 |
| N | -1.0084 | 3.1025 | 0.1760 |
| H | -1.8508 | 2.5895 | 0.4984 |
| H | -0.4838 | 3.4102 | 1.0048 |
| C | -1.4062 | 4.2781 | -0.6525 |
| H | -2.3233 | 4.0065 | -1.2061 |
| H | -1.6390 | 5.1603 | -0.0327 |
| N | 0.0328 | 5.7090 | -2.2351 |
| C | -0.6746 | 6.9853 | -2.0912 |
| H | -0.6613 | 7.3080 | -1.0388 |
| H | -1.7155 | 6.8835 | -2.4335 |
| H | -0.1646 | 7.7399 | -2.7036 |
| C | 1.4691 | 4.1327 | -2.8626 |
| H | 2.2575 | 3.5464 | -3.3254 |
| C | 1.1433 | 5.4613 | -3.0371 |
| H | 1.5803 | 6.2360 | -3.6606 |
| N | -1.9827 | -2.1743 | 0.4856 |
| H | -1.1124 | -2.5884 | 0.8696 |
| H | -2.5202 | -1.7792 | 1.2677 |
| C | -2.7807 | -3.2211 | -0.2176 |
| H | -3.4205 | -3.7872 | 0.4797 |
| H | -2.0683 | -3.9310 | -0.6759 |
| N | -4.6955 | -2.9820 | -1.9217 |

| | | | |
|---|---|---|---|
| C | -5.4071 | -4.2343 | -1.6449 |
| H | -5.6592 | -4.2941 | -0.5754 |
| H | -4.7849 | -5.0973 | -1.9272 |
| H | -6.3345 | -4.2475 | -2.2317 |
| C | -5.0225 | -2.0450 | -2.8963 |
| C | -4.0741 | -1.0451 | -2.8166 |
| H | -3.9792 | -0.1268 | -3.3900 |
| H | -5.8905 | -2.1737 | -3.5375 |
| H | -0.7788 | 0.7902 | -3.6386 |
| Si | 2.5646 | 0.1337 | -4.7744 |
| C | 4.3564 | 0.0883 | -4.0821 |
| H | 4.5865 | 0.8918 | -3.3659 |
| H | 4.5282 | -0.8842 | -3.5921 |
| H | 5.0535 | 0.1486 | -4.9380 |
| O | 0.5683 | -0.4242 | -4.6170 |
| H | 0.3891 | -0.9661 | -3.7548 |
| C | -0.2394 | -1.0475 | -5.6711 |
| H | -1.2862 | -1.0505 | -5.3288 |
| H | -0.1439 | -0.4285 | -6.5724 |
| H | 0.0977 | -2.0757 | -5.8700 |
| C | 2.2066 | 1.8595 | -5.4537 |
| C | 0.9066 | 2.3501 | -5.7241 |
| C | 3.2989 | 2.7347 | -5.6790 |
| C | 0.7037 | 3.6526 | -6.2046 |
| H | 0.0345 | 1.7193 | -5.5331 |
| C | 3.0999 | 4.0425 | -6.1548 |
| H | 4.3220 | 2.4002 | -5.4773 |
| C | 1.8003 | 4.5049 | -6.4185 |
| H | -0.3138 | 4.0078 | -6.3987 |
| H | 3.9610 | 4.6994 | -6.3161 |
| H | 1.6421 | 5.5252 | -6.7823 |
| C | 2.7667 | -1.2245 | -6.1006 |
| H | 2.6891 | -2.2240 | -5.6364 |
| H | 2.0422 | -1.1703 | -6.9262 |
| H | 3.7799 | -1.1382 | -6.5274 |

**Stationary point: I2’**

| | | x | y | z |
|---|---|---|---|---|
| Mo | 0.4055 | 1.5014 | 0.1613 | |
| Mo | 1.1154 | -1.0630 | 0.2581 | |
| S | -0.4690 | -1.9677 | -1.3489 | |
| S | 1.8997 | 0.4743 | -1.3437 | |
| S | -1.4045 | 1.3229 | -1.4806 | |
| S | -0.1086 | 0.0465 | 1.9811 | |
| Cl | 0.7902 | -3.1713 | 1.6593 | |

| | | | |
|---|---|---|---|
| Cl | 2.2151 | 2.4070 | 1.6635 |
| Mo | -1.5595 | -0.4288 | 0.1592 |
| Cl | -3.3028 | 0.9063 | 1.4386 |
| N | -3.3898 | -1.1609 | -0.8249 |
| C | -3.8719 | -2.3297 | -0.3699 |
| C | -0.0085 | 4.4407 | -0.5189 |
| N | 0.7969 | 3.4192 | -0.8594 |
| C | 3.9269 | -2.1536 | -0.1036 |
| N | 2.6936 | -2.3458 | -0.5997 |
| C | 2.7720 | -3.3892 | -1.5049 |
| H | 1.8958 | -3.7515 | -2.0361 |
| N | 2.9197 | -0.7532 | 1.5737 |
| H | 2.9128 | 0.2358 | 1.8895 |
| H | 2.8103 | -1.3520 | 2.4020 |
| C | 4.2035 | -1.0567 | 0.8740 |
| H | 4.5129 | -0.1421 | 0.3361 |
| H | 5.0044 | -1.3207 | 1.5842 |
| N | 4.7982 | -3.0375 | -0.6617 |
| C | 6.2381 | -3.1156 | -0.3930 |
| H | 6.6356 | -4.0185 | -0.8738 |
| H | 6.4133 | -3.1812 | 0.6913 |
| H | 6.7504 | -2.2295 | -0.7980 |
| C | 4.0810 | -3.8243 | -1.5567 |
| H | 4.5621 | -4.6120 | -2.1304 |
| N | -0.8918 | 3.0450 | 1.2363 |
| H | -1.7803 | 2.5871 | 1.5195 |
| H | -0.3879 | 3.3069 | 2.0926 |
| C | -1.1680 | 4.2547 | 0.4081 |
| H | -2.0892 | 4.0666 | -0.1730 |
| H | -1.3454 | 5.1477 | 1.0304 |
| N | 0.3726 | 5.5728 | -1.1701 |
| C | -0.2748 | 6.8848 | -1.0619 |
| H | -0.2753 | 7.2185 | -0.0130 |
| H | -1.3092 | 6.8295 | -1.4335 |
| H | 0.2898 | 7.6053 | -1.6670 |
| C | 1.7294 | 3.9133 | -1.7551 |
| H | 2.5079 | 3.2806 | -2.1731 |
| C | 1.4721 | 5.2533 | -1.9602 |
| H | 1.9621 | 6.0004 | -2.5792 |
| N | -2.2790 | -2.1570 | 1.4236 |
| H | -1.4464 | -2.6517 | 1.7956 |
| H | -2.7934 | -1.7544 | 2.2183 |
| C | -3.1476 | -3.1137 | 0.6771 |
| H | -3.8402 | -3.6504 | 1.3468 |
| H | -2.4928 | -3.8662 | 0.2012 |
| N | -5.0127 | -2.6549 | -1.0351 |

| | | | |
|---|---|---|---|
| C | -5.8151 | -3.8650 | -0.8240 |
| H | -5.2257 | -4.7590 | -1.0774 |
| H | -6.6990 | -3.8172 | -1.4726 |
| H | -6.1418 | -3.9209 | 0.2255 |
| C | -5.2648 | -1.6432 | -1.9560 |
| C | -4.2515 | -0.7175 | -1.8138 |
| H | -4.0884 | 0.2221 | -2.3348 |
| H | -6.1318 | -1.6746 | -2.6105 |
| H | -0.1875 | -1.1874 | -2.4749 |
| H | -0.7032 | 0.7515 | -2.5459 |

**Stationary point: TS3'**

| | | x | y | z |
|---|---|---|---|---|
| Mo | 0.4928 | 1.4775 | -0.8332 | |
| Mo | 1.2207 | -1.1029 | -0.7329 | |
| S | -0.3613 | -1.9053 | -2.3355 | |
| S | 2.0240 | 0.4559 | -2.2784 | |
| S | -1.2424 | 1.2096 | -2.4676 | |
| S | -0.0048 | 0.0137 | 0.9835 | |
| Cl | 0.9136 | -3.1963 | 0.6921 | |
| Cl | 2.2885 | 2.4073 | 0.6816 | |
| Mo | -1.4620 | -0.4651 | -0.8313 | |
| Cl | -3.2174 | 0.8304 | 0.4880 | |
| N | -3.2869 | -1.1837 | -1.8295 | |
| C | -3.7754 | -2.3552 | -1.3878 | |
| C | 0.0375 | 4.4012 | -1.5347 | |
| N | 0.8618 | 3.3910 | -1.8633 | |
| C | 4.0191 | -2.2214 | -1.1046 | |
| N | 2.7827 | -2.3897 | -1.6026 | |
| C | 2.8430 | -3.4277 | -2.5154 | |
| H | 1.9610 | -3.7707 | -3.0498 | |
| N | 3.0397 | -0.8032 | 0.5782 | |
| H | 3.0464 | 0.1874 | 0.8857 | |
| H | 2.9218 | -1.3913 | 1.4129 | |
| C | 4.3177 | -1.1360 | -0.1194 | |
| H | 4.6553 | -0.2277 | -0.6507 | |
| H | 5.1088 | -1.4244 | 0.5921 | |
| N | 4.8747 | -3.1161 | -1.6694 | |
| C | 6.3135 | -3.2197 | -1.4033 | |
| H | 6.6953 | -4.1259 | -1.8906 | |
| H | 6.4888 | -3.2952 | -0.3198 | |
| H | 6.8397 | -2.3397 | -1.8035 | |
| C | 4.1443 | -3.8846 | -2.5696 | |
| H | 4.6120 | -4.6761 | -3.1491 | |
| N | -0.8158 | 3.0105 | 0.2386 | |

| | | | |
|---|---|---|---|
| H | -1.6945 | 2.5416 | 0.5325 |
| H | -0.3114 | 3.2929 | 1.0883 |
| C | -1.1183 | 4.2029 | -0.6051 |
| H | -2.0353 | 3.9862 | -1.1831 |
| H | -1.3148 | 5.0999 | 0.0057 |
| N | 0.3977 | 5.5320 | -2.1994 |
| C | -0.2742 | 6.8330 | -2.1071 |
| H | -0.2748 | 7.1828 | -1.0636 |
| H | -1.3094 | 6.7517 | -2.4714 |
| H | 0.2726 | 7.5545 | -2.7271 |
| C | 1.7846 | 3.8910 | -2.7657 |
| H | 2.5745 | 3.2686 | -3.1781 |
| C | 1.5027 | 5.2236 | -2.9860 |
| H | 1.9792 | 5.9721 | -3.6137 |
| N | -2.1891 | -2.2142 | 0.4149 |
| H | -1.3560 | -2.7113 | 0.7817 |
| H | -2.7057 | -1.8242 | 1.2141 |
| C | -3.0531 | -3.1576 | -0.3527 |
| H | -3.7472 | -3.7086 | 0.3035 |
| H | -2.3938 | -3.8981 | -0.8413 |
| N | -4.9228 | -2.6612 | -2.0508 |
| C | -5.7367 | -3.8654 | -1.8497 |
| H | -5.1579 | -4.7624 | -2.1166 |
| H | -6.6234 | -3.8008 | -2.4929 |
| H | -6.0585 | -3.9300 | -0.7992 |
| C | -5.1715 | -1.6349 | -2.9563 |
| C | -4.1498 | -0.7195 | -2.8073 |
| H | -3.9814 | 0.2256 | -3.3165 |
| H | -6.0420 | -1.6504 | -3.6066 |
| H | -0.1309 | -0.7662 | -3.4726 |
| H | -0.4200 | 0.2787 | -3.5162 |

**Stationary point: TS1’’**

| | | x | y | z |
|---|---|---|---|---|
| Mo | 0.0005 | 0.0206 | 0.0011 | |
| Mo | -0.0455 | 0.0719 | 2.6971 | |
| S | 2.3999 | 0.0601 | 2.9947 | |
| S | 0.0382 | 1.9470 | 1.3616 | |
| S | 2.3355 | -0.0788 | -0.4054 | |
| S | -0.5754 | -1.8462 | 1.3911 | |
| Cl | -0.1342 | -1.4534 | 4.7089 | |
| Cl | -2.5376 | 0.2855 | -0.2857 | |
| Mo | 1.7755 | -1.5741 | 1.2762 | |
| Cl | 1.7252 | -3.5659 | -0.2869 | |
| N | 3.9011 | -2.1496 | 1.3644 | |

| | | | |
|---|---|---|---|
| C | 4.2614 | -2.9371 | 2.3929 |
| C | 0.0431 | 0.6542 | -2.9703 |
| N | 0.0027 | 1.3001 | -1.7915 |
| C | -1.5361 | 1.9958 | 4.5105 |
| N | -0.2830 | 1.5617 | 4.2985 |
| C | 0.5305 | 2.1697 | 5.2379 |
| H | 1.5973 | 1.9679 | 5.2850 |
| N | -2.2651 | 0.2760 | 2.9865 |
| H | -2.6935 | 0.1962 | 2.0427 |
| H | -2.5954 | -0.5168 | 3.5512 |
| C | -2.6593 | 1.5725 | 3.6185 |
| H | -2.7940 | 2.3105 | 2.8068 |
| H | -3.6158 | 1.4851 | 4.1591 |
| N | -1.5568 | 2.8667 | 5.5559 |
| C | -2.7339 | 3.5817 | 6.0616 |
| H | -3.5528 | 2.8708 | 6.2461 |
| H | -3.0595 | 4.3416 | 5.3348 |
| H | -2.4669 | 4.0713 | 7.0069 |
| C | -0.2528 | 2.9905 | 6.0241 |
| H | -0.0113 | 3.6355 | 6.8647 |
| N | -0.3128 | -1.3978 | -1.7532 |
| H | 0.1745 | -2.2886 | -1.5449 |
| H | -1.3207 | -1.5939 | -1.8021 |
| C | 0.1703 | -0.8356 | -3.0484 |
| H | 1.2316 | -1.1184 | -3.1683 |
| H | -0.3828 | -1.2514 | -3.9071 |
| N | 0.0019 | 1.5495 | -3.9941 |
| C | 0.0528 | 1.2271 | -5.4243 |
| H | -0.7244 | 0.4880 | -5.6699 |
| H | 1.0427 | 0.8257 | -5.6894 |
| H | -0.1334 | 2.1447 | -5.9969 |
| C | -0.0683 | 2.6544 | -2.0676 |
| H | -0.1218 | 3.3964 | -1.2755 |
| C | -0.0648 | 2.8225 | -3.4369 |
| H | -0.1100 | 3.7122 | -4.0594 |
| N | 1.9199 | -3.1589 | 2.8980 |
| H | 1.2407 | -2.9318 | 3.6475 |
| H | 1.6221 | -4.0386 | 2.4561 |
| C | 3.2902 | -3.3055 | 3.4683 |
| H | 3.4663 | -4.3222 | 3.8576 |
| H | 3.3816 | -2.6015 | 4.3153 |
| N | 5.5818 | -3.2509 | 2.2999 |
| C | 6.3471 | -4.0571 | 3.2573 |
| H | 5.8228 | -5.0046 | 3.4514 |
| H | 6.4777 | -3.5057 | 4.2011 |
| H | 7.3310 | -4.2762 | 2.8232 |

| | | | |
|---|---|---|---|
| C | 6.0820 | -2.6300 | 1.1597 |
| C | 5.0280 | -1.9522 | 0.5837 |
| H | 4.9968 | -1.3553 | -0.3237 |
| H | 7.1236 | -2.7343 | 0.8679 |
| H | 2.8317 | 0.9858 | 1.9987 |
| O | 3.3855 | 1.3560 | 0.4582 |
| C | 3.1754 | 2.6124 | -0.1906 |
| H | 3.2241 | 2.5106 | -1.2932 |
| H | 2.1994 | 3.0606 | 0.0746 |
| H | 3.9873 | 3.2894 | 0.1331 |

**Stationary point: I1’’**

| | x | y | z |
|---|---|---|---|
| Mo | -0.7884 | 0.3075 | -1.2583 |
| Mo | -0.8312 | 0.3320 | 1.4110 |
| S | 1.6022 | 0.3040 | 1.7834 |
| S | -0.6938 | 2.2393 | 0.0903 |
| S | 1.5576 | 0.1587 | -1.7132 |
| S | -1.3522 | -1.5833 | 0.0978 |
| Cl | -0.9579 | -1.2202 | 3.4130 |
| Cl | -3.3176 | 0.5635 | -1.5553 |
| Mo | 1.0019 | -1.2921 | 0.0270 |
| Cl | 0.9864 | -3.2514 | -1.5945 |
| N | 3.1240 | -1.8659 | 0.1493 |
| C | 3.4609 | -2.6895 | 1.1573 |
| C | -0.7636 | 0.9644 | -4.2307 |
| N | -0.8131 | 1.6004 | -3.0468 |
| C | -2.3338 | 2.2547 | 3.2171 |
| N | -1.0813 | 1.8096 | 3.0258 |
| C | -0.2810 | 2.4018 | 3.9865 |
| H | 0.7826 | 2.1891 | 4.0539 |
| N | -3.0496 | 0.5472 | 1.6764 |
| H | -3.4711 | 0.4672 | 0.7288 |
| H | -3.3877 | -0.2419 | 2.2416 |
| C | -3.4425 | 1.8475 | 2.3004 |
| H | -3.5487 | 2.5871 | 1.4860 |
| H | -4.4120 | 1.7718 | 2.8193 |
| N | -2.3669 | 3.1174 | 4.2690 |
| C | -3.5473 | 3.8379 | 4.7589 |
| H | -4.3746 | 3.1320 | 4.9241 |
| H | -3.8545 | 4.6052 | 4.0320 |
| H | -3.2933 | 4.3189 | 5.7122 |
| C | -1.0713 | 3.2244 | 4.7638 |
| H | -0.8400 | 3.8604 | 5.6142 |
| N | -1.0700 | -1.1059 | -3.0326 |

| | | | |
|---|---|---|---|
| H | -0.5663 | -1.9900 | -2.8320 |
| H | -2.0738 | -1.3202 | -3.0834 |
| C | -0.5987 | -0.5208 | -4.3223 |
| H | 0.4692 | -0.7771 | -4.4433 |
| H | -1.1402 | -0.9414 | -5.1860 |
| N | -0.8548 | 1.8643 | -5.2475 |
| C | -0.8180 | 1.5531 | -6.6806 |
| H | -1.5928 | 0.8103 | -6.9232 |
| H | 0.1718 | 1.1614 | -6.9600 |
| H | -1.0167 | 2.4736 | -7.2439 |
| C | -0.9482 | 2.9519 | -3.3126 |
| H | -1.0259 | 3.6850 | -2.5142 |
| C | -0.9684 | 3.1295 | -4.6803 |
| H | -1.0577 | 4.0209 | -5.2956 |
| N | 1.1075 | -2.9294 | 1.5961 |
| H | 0.4145 | -2.7190 | 2.3376 |
| H | 0.8139 | -3.7938 | 1.1221 |
| C | 2.4639 | -3.1026 | 2.1922 |
| H | 2.6311 | -4.1355 | 2.5408 |
| H | 2.5371 | -2.4377 | 3.0719 |
| N | 4.7861 | -2.9889 | 1.0915 |
| C | 5.5289 | -3.8261 | 2.0400 |
| H | 5.0160 | -4.7907 | 2.1689 |
| H | 5.6117 | -3.3175 | 3.0128 |
| H | 6.5333 | -4.0068 | 1.6362 |
| C | 5.3141 | -2.3206 | -0.0086 |
| C | 4.2718 | -1.6287 | -0.5890 |
| H | 4.2627 | -0.9946 | -1.4713 |
| H | 6.3645 | -2.4063 | -0.2738 |
| H | 2.0175 | 1.3439 | 0.9848 |
| O | 2.5728 | 1.5508 | -1.0782 |
| C | 2.3784 | 2.7380 | -1.8605 |
| H | 2.4578 | 2.5377 | -2.9474 |
| H | 1.3952 | 3.2008 | -1.6559 |
| H | 3.1814 | 3.4363 | -1.5678 |

## Concerted mechanism

## Cluster $3^+$

**Stationary point: TS1**

| | | x | y | z |
|---|---|---|---|---|
| Mo | 0.4162 | 1.3920 | 0.9821 | |
| Mo | 1.0972 | -1.2560 | 0.9623 | |
| S | -0.5260 | -2.0392 | -0.5614 | |

| | | | |
|---|---|---|---|
| S | 1.9417 | 0.3847 | -0.4767 |
| S | -1.3502 | 1.2769 | -0.5740 |
| S | -0.0567 | -0.1466 | 2.7321 |
| Cl | 0.8172 | -3.3426 | 2.4333 |
| Cl | 2.2964 | 2.1852 | 2.5483 |
| Mo | -1.5488 | -0.5100 | 0.9222 |
| Cl | -3.2479 | 0.7629 | 2.3755 |
| N | -3.4285 | -1.0966 | -0.0676 |
| C | -3.9569 | -2.2672 | 0.3261 |
| C | 0.1224 | 4.3631 | 0.4095 |
| N | 0.8989 | 3.3289 | 0.0446 |
| C | 3.8479 | -2.4461 | 0.4739 |
| N | 2.5889 | -2.5776 | 0.0244 |
| C | 2.5818 | -3.6259 | -0.8793 |
| H | 1.6667 | -3.9513 | -1.3678 |
| N | 2.9821 | -1.0289 | 2.2207 |
| H | 3.0217 | -0.0439 | 2.5448 |
| H | 2.8816 | -1.6325 | 3.0464 |
| C | 4.2164 | -1.3811 | 1.4594 |
| H | 4.5495 | -0.4734 | 0.9243 |
| H | 5.0354 | -1.6999 | 2.1254 |
| N | 4.6538 | -3.3711 | -0.1180 |
| C | 6.0953 | -3.5272 | 0.1051 |
| H | 6.2959 | -3.6574 | 1.1793 |
| H | 6.6368 | -2.6452 | -0.2687 |
| H | 6.4389 | -4.4197 | -0.4332 |
| C | 3.8658 | -4.1231 | -0.9823 |
| H | 4.2850 | -4.9333 | -1.5730 |
| N | -0.7935 | 2.9484 | 2.1251 |
| H | -1.6807 | 2.5053 | 2.4293 |
| H | -0.2605 | 3.2054 | 2.9661 |
| C | -1.0682 | 4.1584 | 1.2931 |
| H | -1.9640 | 3.9515 | 0.6798 |
| H | -1.2815 | 5.0461 | 1.9114 |
| N | 0.6135 | 5.5207 | -0.1122 |
| C | -0.0027 | 6.8454 | -0.0038 |
| H | -0.2498 | 7.0610 | 1.0462 |
| H | -0.9174 | 6.8849 | -0.6154 |
| H | 0.7119 | 7.5967 | -0.3637 |
| C | 1.9209 | 3.8364 | -0.7369 |
| H | 2.6943 | 3.1951 | -1.1509 |
| C | 1.7515 | 5.2013 | -0.8459 |
| H | 2.3166 | 5.9637 | -1.3753 |
| N | -2.3262 | -2.3004 | 2.0974 |
| H | -1.5107 | -2.8466 | 2.4286 |
| H | -2.8197 | -1.9322 | 2.9208 |

| | | | |
|---|---|---|---|
| C | -3.2362 | -3.1611 | 1.2863 |
| H | -3.9334 | -3.7375 | 1.9171 |
| H | -2.6087 | -3.8814 | 0.7308 |
| N | -5.1563 | -2.4671 | -0.2874 |
| C | -6.0231 | -3.6394 | -0.1246 |
| H | -5.5386 | -4.5335 | -0.5452 |
| H | -6.9666 | -3.4540 | -0.6536 |
| H | -6.2372 | -3.8010 | 0.9427 |
| C | -5.3969 | -1.3694 | -1.1072 |
| C | -4.3193 | -0.5201 | -0.9559 |
| H | -4.1329 | 0.4559 | -1.3963 |
| H | -6.3008 | -1.2955 | -1.7061 |
| H | -0.6971 | 0.7590 | -1.9606 |
| Si | -0.7041 | 0.7147 | -3.8459 |
| C | -2.2293 | -0.3563 | -4.1337 |
| H | -1.9302 | -1.2969 | -4.6223 |
| H | -2.7331 | -0.6070 | -3.1854 |
| H | -2.9527 | 0.1670 | -4.7823 |
| C | 0.4266 | 0.9114 | -5.3524 |
| H | -0.0612 | 1.6132 | -6.0494 |
| H | 1.4079 | 1.3414 | -5.0887 |
| H | 0.5975 | -0.0447 | -5.8720 |
| C | -1.2379 | 2.5171 | -3.4884 |
| C | -0.2922 | 3.5656 | -3.4114 |
| C | -2.5920 | 2.8243 | -3.2054 |
| C | -0.6861 | 4.8757 | -3.0920 |
| H | 0.7694 | 3.3629 | -3.5947 |
| C | -2.9908 | 4.1329 | -2.8852 |
| H | -3.3511 | 2.0345 | -3.2396 |
| C | -2.0362 | 5.1631 | -2.8267 |
| H | 0.0630 | 5.6726 | -3.0494 |
| H | -4.0459 | 4.3483 | -2.6843 |
| H | -2.3456 | 6.1860 | -2.5868 |
| O | 0.4184 | -0.9218 | -3.2723 |
| H | 0.1830 | -1.1788 | -2.3234 |
| C | 1.8500 | -1.1003 | -3.5021 |
| H | 2.4005 | -0.1817 | -3.2443 |
| H | 2.2033 | -1.9267 | -2.8688 |
| H | 1.9875 | -1.3524 | -4.5617 |

**Stationary point: I1**

| | x | y | z |
|---|---|---|---|
| Mo | 0.4055 | 1.5014 | 0.1613 |
| Mo | 1.1154 | -1.0630 | 0.2581 |
| S | -0.4690 | -1.9677 | -1.3489 |

| | | | |
|---|---|---|---|
| S | 1.8997 | 0.4743 | -1.3437 |
| S | -1.4045 | 1.3229 | -1.4806 |
| S | -0.1086 | 0.0465 | 1.9811 |
| Cl | 0.7902 | -3.1713 | 1.6593 |
| Cl | 2.2151 | 2.4070 | 1.6635 |
| Mo | -1.5595 | -0.4288 | 0.1592 |
| Cl | -3.3028 | 0.9063 | 1.4386 |
| N | -3.3898 | -1.1609 | -0.8249 |
| C | -3.8719 | -2.3297 | -0.3699 |
| C | -0.0085 | 4.4407 | -0.5189 |
| N | 0.7969 | 3.4192 | -0.8594 |
| C | 3.9269 | -2.1536 | -0.1036 |
| N | 2.6936 | -2.3458 | -0.5997 |
| C | 2.7720 | -3.3892 | -1.5049 |
| H | 1.8958 | -3.7515 | -2.0361 |
| N | 2.9197 | -0.7532 | 1.5737 |
| H | 2.9128 | 0.2358 | 1.8895 |
| H | 2.8103 | -1.3520 | 2.4020 |
| C | 4.2035 | -1.0567 | 0.8740 |
| H | 4.5129 | -0.1421 | 0.3361 |
| H | 5.0044 | -1.3207 | 1.5842 |
| N | 4.7982 | -3.0375 | -0.6617 |
| C | 6.2381 | -3.1156 | -0.3930 |
| H | 6.6356 | -4.0185 | -0.8738 |
| H | 6.4133 | -3.1812 | 0.6913 |
| H | 6.7504 | -2.2295 | -0.7980 |
| C | 4.0810 | -3.8243 | -1.5567 |
| H | 4.5621 | -4.6120 | -2.1304 |
| N | -0.8918 | 3.0450 | 1.2363 |
| H | -1.7803 | 2.5871 | 1.5195 |
| H | -0.3879 | 3.3069 | 2.0926 |
| C | -1.1680 | 4.2547 | 0.4081 |
| H | -2.0892 | 4.0666 | -0.1730 |
| H | -1.3454 | 5.1477 | 1.0304 |
| N | 0.3726 | 5.5728 | -1.1701 |
| C | -0.2748 | 6.8848 | -1.0619 |
| H | -0.2753 | 7.2185 | -0.0130 |
| H | -1.3092 | 6.8295 | -1.4335 |
| H | 0.2898 | 7.6053 | -1.6670 |
| C | 1.7294 | 3.9133 | -1.7551 |
| H | 2.5079 | 3.2806 | -2.1731 |
| C | 1.4721 | 5.2533 | -1.9602 |
| H | 1.9621 | 6.0004 | -2.5792 |
| N | -2.2790 | -2.1570 | 1.4236 |
| H | -1.4464 | -2.6517 | 1.7956 |
| H | -2.7934 | -1.7544 | 2.2183 |

| | | | |
|---|---|---|---|
| C | -3.1476 | -3.1137 | 0.6771 |
| H | -3.8402 | -3.6504 | 1.3468 |
| H | -2.4928 | -3.8662 | 0.2012 |
| N | -5.0127 | -2.6549 | -1.0351 |
| C | -5.8151 | -3.8650 | -0.8240 |
| H | -5.2257 | -4.7590 | -1.0774 |
| H | -6.6990 | -3.8172 | -1.4726 |
| H | -6.1418 | -3.9209 | 0.2255 |
| C | -5.2648 | -1.6432 | -1.9560 |
| C | -4.2515 | -0.7175 | -1.8138 |
| H | -4.0884 | 0.2221 | -2.3348 |
| H | -6.1318 | -1.6746 | -2.6105 |
| H | -0.1875 | -1.1874 | -2.4749 |
| H | -0.7032 | 0.7515 | -2.5459 |

**Stationary point: TS2**

| | | x | y | z |
|---|---|---|---|---|
| Mo | 0.4928 | 1.4775 | -0.8332 | |
| Mo | 1.2207 | -1.1029 | -0.7329 | |
| S | -0.3613 | -1.9053 | -2.3355 | |
| S | 2.0240 | 0.4559 | -2.2784 | |
| S | -1.2424 | 1.2096 | -2.4676 | |
| S | -0.0048 | 0.0137 | 0.9835 | |
| Cl | 0.9136 | -3.1963 | 0.6921 | |
| Cl | 2.2885 | 2.4073 | 0.6816 | |
| Mo | -1.4620 | -0.4651 | -0.8313 | |
| Cl | -3.2174 | 0.8304 | 0.4880 | |
| N | -3.2869 | -1.1837 | -1.8295 | |
| C | -3.7754 | -2.3552 | -1.3878 | |
| C | 0.0375 | 4.4012 | -1.5347 | |
| N | 0.8618 | 3.3910 | -1.8633 | |
| C | 4.0191 | -2.2214 | -1.1046 | |
| N | 2.7827 | -2.3897 | -1.6026 | |
| C | 2.8430 | -3.4277 | -2.5154 | |
| H | 1.9610 | -3.7707 | -3.0498 | |
| N | 3.0397 | -0.8032 | 0.5782 | |
| H | 3.0464 | 0.1874 | 0.8857 | |
| H | 2.9218 | -1.3913 | 1.4129 | |
| C | 4.3177 | -1.1360 | -0.1194 | |
| H | 4.6553 | -0.2277 | -0.6507 | |
| H | 5.1088 | -1.4244 | 0.5921 | |
| N | 4.8747 | -3.1161 | -1.6694 | |
| C | 6.3135 | -3.2197 | -1.4033 | |
| H | 6.6953 | -4.1259 | -1.8906 | |
| H | 6.4888 | -3.2952 | -0.3198 | |

| | | | |
|---|---|---|---|
| H | 6.8397 | -2.3397 | -1.8035 |
| C | 4.1443 | -3.8846 | -2.5696 |
| H | 4.6120 | -4.6761 | -3.1491 |
| N | -0.8158 | 3.0105 | 0.2386 |
| H | -1.6945 | 2.5416 | 0.5325 |
| H | -0.3114 | 3.2929 | 1.0883 |
| C | -1.1183 | 4.2029 | -0.6051 |
| H | -2.0353 | 3.9862 | -1.1831 |
| H | -1.3148 | 5.0999 | 0.0057 |
| N | 0.3977 | 5.5320 | -2.1994 |
| C | -0.2742 | 6.8330 | -2.1071 |
| H | -0.2748 | 7.1828 | -1.0636 |
| H | -1.3094 | 6.7517 | -2.4714 |
| H | 0.2726 | 7.5545 | -2.7271 |
| C | 1.7846 | 3.8910 | -2.7657 |
| H | 2.5745 | 3.2686 | -3.1781 |
| C | 1.5027 | 5.2236 | -2.9860 |
| H | 1.9792 | 5.9721 | -3.6137 |
| N | -2.1891 | -2.2142 | 0.4149 |
| H | -1.3560 | -2.7113 | 0.7817 |
| H | -2.7057 | -1.8242 | 1.2141 |
| C | -3.0531 | -3.1576 | -0.3527 |
| H | -3.7472 | -3.7086 | 0.3035 |
| H | -2.3938 | -3.8981 | -0.8413 |
| N | -4.9228 | -2.6612 | -2.0508 |
| C | -5.7367 | -3.8654 | -1.8497 |
| H | -5.1579 | -4.7624 | -2.1166 |
| H | -6.6234 | -3.8008 | -2.4929 |
| H | -6.0585 | -3.9300 | -0.7992 |
| C | -5.1715 | -1.6349 | -2.9563 |
| C | -4.1498 | -0.7195 | -2.8073 |
| H | -3.9814 | 0.2256 | -3.3165 |
| H | -6.0420 | -1.6504 | -3.6066 |
| H | -0.1309 | -0.7662 | -3.4726 |
| H | -0.4200 | 0.2787 | -3.5162 |

## Cluster $2^+$

**Stationary point: Cluster $2^+$**

| | x | y | z |
|---|---|---|---|
| S | 1.2213 | 0.5573 | 0.8942 |
| Mo | -0.9090 | 1.5088 | 0.7810 |
| N | 0.0802 | 3.4997 | 0.1790 |
| C | 1.2406 | 3.5209 | -0.7569 |
| Mo | -0.2917 | -1.1205 | 1.4403 |

| | | | |
|---|---|---|---|
| S | -0.4720 | -2.0741 | -0.6839 |
| Mo | -2.2445 | -0.5878 | -0.4658 |
| N | -2.8530 | -1.3597 | -2.5476 |
| C | -1.9193 | -1.2499 | -3.7046 |
| N | 1.5945 | -2.3944 | 1.7887 |
| C | 2.2739 | -1.9250 | 3.0361 |
| C | 1.2458 | -1.6965 | 4.1327 |
| N | 0.1956 | -0.7326 | 3.6815 |
| C | -0.9010 | -0.6466 | 4.6898 |
| C | 2.5850 | -2.6049 | 0.6944 |
| Cl | -1.3136 | -3.1985 | 2.4518 |
| S | -2.3662 | -0.0382 | 1.8385 |
| N | -2.6913 | 3.0039 | 0.7915 |
| C | -2.1655 | 4.3991 | 0.6817 |
| C | -0.9811 | 4.4535 | -0.2700 |
| Cl | -0.5583 | 2.5569 | 3.0528 |
| S | -1.2391 | 1.2464 | -1.5045 |
| C | -3.7412 | 2.9236 | 1.8485 |
| N | -3.6134 | -2.4040 | 0.0270 |
| C | -4.3053 | -2.8538 | -1.2196 |
| C | -3.3727 | -2.7563 | -2.4165 |
| Cl | -4.5049 | 0.5116 | -0.7281 |
| C | -4.5774 | -2.3372 | 1.1639 |
| H | 1.1619 | -3.3080 | 1.9984 |
| H | 0.6137 | 0.2102 | 3.6290 |
| H | 0.4157 | 3.8224 | 1.1003 |
| H | -3.1561 | 2.7571 | -0.0970 |
| H | -3.6611 | -0.7464 | -2.7385 |
| H | -2.9093 | -3.1134 | 0.2861 |
| H | 2.0851 | -3.0705 | -0.1657 |
| H | 3.4059 | -3.2579 | 1.0392 |
| H | 2.9977 | -1.6331 | 0.3859 |
| H | 2.8110 | -0.9922 | 2.7930 |
| H | 3.0206 | -2.6688 | 3.3692 |
| H | 0.7317 | -2.6403 | 4.3802 |
| H | 1.7395 | -1.3249 | 5.0483 |
| H | -1.5223 | 0.2304 | 4.4643 |
| H | -0.4774 | -0.5395 | 5.7039 |
| H | -1.5088 | -1.5610 | 4.6351 |
| H | 2.0562 | 2.9144 | -0.3406 |
| H | 1.5936 | 4.5559 | -0.9090 |
| H | 0.9351 | 3.0943 | -1.7237 |
| H | -0.5790 | 5.4822 | -0.3123 |
| H | -1.2793 | 4.1635 | -1.2920 |
| H | -2.9624 | 5.0833 | 0.3394 |
| H | -1.8599 | 4.7099 | 1.6947 |

| | | | |
|---|---|---|---|
| H | -4.2766 | 1.9704 | 1.7455 |
| H | -4.4584 | 3.7558 | 1.7359 |
| H | -3.2593 | 2.9785 | 2.8351 |
| H | -1.6521 | -0.1958 | -3.8591 |
| H | -2.3930 | -1.6467 | -4.6196 |
| H | -1.0056 | -1.8230 | -3.4891 |
| H | -2.5033 | -3.4256 | -2.2986 |
| H | -3.9083 | -3.0538 | -3.3366 |
| H | -5.1799 | -2.1965 | -1.3588 |
| H | -4.6700 | -3.8900 | -1.1028 |
| H | -4.0139 | -2.2536 | 2.1027 |
| H | -5.1953 | -3.2520 | 1.1925 |
| H | -5.2224 | -1.4566 | 1.0335 |

**Stationary point: TS1**

| | | x | y | z |
|---|---|---|---|---|
| C | 1.4802 | 2.0249 | -1.7900 | |
| C | 0.6631 | 1.3701 | -0.8518 | |
| C | 1.1656 | 0.2678 | -0.1375 | |
| C | 2.4739 | -0.1827 | -0.3749 | |
| C | 3.3038 | 0.4491 | -1.3263 | |
| C | 2.7893 | 1.5690 | -2.0217 | |
| Si | 5.0566 | -0.1491 | -1.6753 | |
| C | 5.6949 | 0.4240 | -3.3956 | |
| C | 6.2474 | 0.8508 | -0.5664 | |
| O | 5.2312 | -1.6877 | -0.4434 | |
| C | 5.8383 | -1.7817 | 0.8902 | |
| C | 1.7989 | -0.6508 | -4.1407 | |
| N | 1.6903 | -1.9223 | -4.9018 | |
| C | 2.1804 | -1.7426 | -6.3003 | |
| C | 2.0405 | -3.0486 | -7.0640 | |
| N | 2.7697 | -4.1464 | -6.3622 | |
| C | 2.4679 | -5.4567 | -7.0071 | |
| Mo | 2.5422 | -3.8934 | -4.0574 | |
| S | 4.5821 | -2.7349 | -3.9865 | |
| Mo | 4.9773 | -4.9680 | -3.3952 | |
| N | 7.2169 | -4.4310 | -3.1967 | |
| C | 7.9215 | -5.5457 | -2.4947 | |
| C | 7.4596 | -6.8809 | -3.0560 | |
| N | 5.9755 | -7.0172 | -2.9481 | |
| C | 5.5199 | -8.2466 | -3.6584 | |
| S | 1.9036 | -3.1727 | -1.9183 | |
| Mo | 2.7814 | -5.3127 | -1.7245 | |
| N | 0.6520 | -6.2231 | -1.7327 | |
| C | 0.3524 | -7.4444 | -2.5339 | |

| | | | |
|---|---|---|---|
| Cl | 0.1443 | -4.5558 | -4.5998 |
| S | 3.0239 | -6.2092 | -3.9100 |
| Cl | 3.3394 | -7.6310 | -0.8413 |
| S | 4.9300 | -4.5857 | -1.0752 |
| N | 2.1174 | -5.1162 | 0.4794 |
| C | 2.5906 | -3.9918 | 1.3310 |
| Cl | 5.8185 | -5.4747 | -5.7393 |
| C | 0.6297 | -5.2376 | 0.5439 |
| C | 0.1700 | -6.3942 | -0.3290 |
| C | 7.6456 | -3.1121 | -2.6566 |
| H | 2.5175 | -5.9885 | 0.8602 |
| H | 0.1278 | -5.4491 | -2.1748 |
| H | 0.6912 | -2.1781 | -4.9516 |
| H | 3.7857 | -3.9969 | -6.4769 |
| H | 7.4924 | -4.4675 | -4.1910 |
| H | 5.7268 | -7.1420 | -1.9535 |
| H | 3.6881 | -3.9954 | 1.3601 |
| H | 2.1969 | -4.0880 | 2.3583 |
| H | 2.2459 | -3.0418 | 0.8975 |
| H | 0.2029 | -4.2817 | 0.1949 |
| H | 0.2995 | -5.3987 | 1.5869 |
| H | 0.5909 | -7.3441 | 0.0423 |
| H | -0.9316 | -6.4767 | -0.3079 |
| H | 0.4796 | -7.2102 | -3.5988 |
| H | -0.6877 | -7.7715 | -2.3562 |
| H | 1.0496 | -8.2421 | -2.2396 |
| H | 1.3634 | -0.7708 | -3.1402 |
| H | 1.2854 | 0.1699 | -4.6722 |
| H | 2.8589 | -0.3934 | -4.0248 |
| H | 1.6116 | -0.9425 | -6.8095 |
| H | 3.2362 | -1.4250 | -6.2453 |
| H | 2.4143 | -2.9335 | -8.0974 |
| H | 0.9797 | -3.3481 | -7.1165 |
| H | 3.1932 | -6.2018 | -6.6546 |
| H | 2.5430 | -5.3692 | -8.1060 |
| H | 1.4500 | -5.7640 | -6.7266 |
| H | 7.2235 | -2.3119 | -3.2793 |
| H | 8.7470 | -3.0306 | -2.6556 |
| H | 7.2726 | -2.9993 | -1.6286 |
| H | 7.6860 | -5.4640 | -1.4196 |
| H | 9.0163 | -5.4455 | -2.6130 |
| H | 7.7124 | -6.9529 | -4.1275 |
| H | 7.9632 | -7.7116 | -2.5292 |
| H | 4.4873 | -8.4680 | -3.3577 |
| H | 6.1662 | -9.1035 | -3.3960 |
| H | 5.5630 | -8.0663 | -4.7423 |

| | | | |
|---|---|---|---|
| H | 3.4058 | 2.0832 | -2.7681 |
| H | 1.0965 | 2.8871 | -2.3456 |
| H | -0.3590 | 1.7204 | -0.6745 |
| H | 0.5366 | -0.2441 | 0.5989 |
| H | 2.8530 | -1.0527 | 0.1704 |
| H | 4.6133 | -1.6702 | -2.6010 |
| H | 4.9603 | -2.6093 | -0.7869 |
| H | 5.2244 | -1.2152 | 1.6045 |
| H | 5.8594 | -2.8462 | 1.1649 |
| H | 6.8629 | -1.3893 | 0.8540 |
| H | 5.9626 | 0.8691 | 0.4971 |
| H | 7.2902 | 0.4963 | -0.6472 |
| H | 6.2246 | 1.8904 | -0.9364 |
| H | 5.0138 | 0.1658 | -4.2214 |
| H | 5.8440 | 1.5183 | -3.3708 |
| H | 6.6745 | -0.0401 | -3.6047 |

**Stationary point: I1**

| | | x | y | z |
|---|---|---|---|---|
| C | -1.6995 | 3.3316 | 2.6394 | |
| N | -1.4497 | 3.3064 | 1.1716 | |
| C | -0.5967 | 4.4652 | 0.7692 | |
| C | -0.3601 | 4.4371 | -0.7306 | |
| N | 0.2607 | 3.1413 | -1.1418 | |
| C | 0.3229 | 3.0578 | -2.6306 | |
| Mo | -0.6816 | 1.4002 | 0.0797 | |
| S | 1.1434 | 1.5972 | 1.6739 | |
| Mo | 1.5947 | -0.1499 | 0.0899 | |
| N | 2.6207 | -1.7983 | -1.1534 | |
| C | 2.5364 | -1.7557 | -2.6436 | |
| Mo | -0.9352 | -1.2606 | 0.0885 | |
| N | -2.8246 | -1.3660 | -1.2016 | |
| C | -2.7202 | -1.3287 | -2.6896 | |
| Cl | -2.6892 | 1.9580 | -1.3020 | |
| S | 0.0352 | -0.0143 | -1.6900 | |
| S | -1.9389 | 0.1743 | 1.6503 | |
| Cl | 3.1043 | 1.4070 | -1.1879 | |
| S | 0.8272 | -1.7861 | 1.6735 | |
| N | 3.6173 | -0.4644 | 1.1855 | |
| C | 4.1837 | -1.7819 | 0.7694 | |
| C | 4.0499 | -1.9341 | -0.7353 | |
| Cl | -0.3083 | -3.3636 | -1.1628 | |
| N | -2.2239 | -2.8558 | 1.1751 | |
| C | -3.6387 | -2.6939 | 0.7291 | |
| C | -3.6740 | -2.5233 | -0.7797 | |

| | | | |
|---|---|---|---|
| C | 3.7541 | -0.2706 | 2.6562 |
| C | -2.1496 | -3.0559 | 2.6490 |
| H | -2.3620 | 3.4147 | 0.7015 |
| H | 1.2425 | 3.1264 | -0.8160 |
| H | 4.1745 | 0.2718 | 0.7233 |
| H | 2.1090 | -2.6483 | -0.8624 |
| H | -1.8543 | -3.7102 | 0.7285 |
| H | -3.2937 | -0.4893 | -0.9132 |
| H | -2.4183 | 2.5444 | 2.9025 |
| H | -2.1000 | 4.3146 | 2.9441 |
| H | -0.7555 | 3.1480 | 3.1736 |
| H | 0.3555 | 4.3869 | 1.3220 |
| H | -1.0784 | 5.4175 | 1.0583 |
| H | -1.3179 | 4.5231 | -1.2719 |
| H | 0.2803 | 5.2848 | -1.0342 |
| H | 0.9944 | 2.2373 | -2.9143 |
| H | 0.7084 | 4.0038 | -3.0510 |
| H | -0.6885 | 2.8637 | -3.0163 |
| H | 3.4624 | 0.7553 | 2.9190 |
| H | 4.7979 | -0.4442 | 2.9718 |
| H | 3.0976 | -0.9790 | 3.1830 |
| H | 5.2462 | -1.8565 | 1.0656 |
| H | 3.6276 | -2.5720 | 1.3027 |
| H | 4.4561 | -2.9077 | -1.0630 |
| H | 4.6140 | -1.1380 | -1.2509 |
| H | 1.4958 | -1.9302 | -2.9469 |
| H | 3.1784 | -2.5389 | -3.0847 |
| H | 2.8652 | -0.7649 | -2.9895 |
| H | -1.1227 | -3.3299 | 2.9271 |
| H | -2.8382 | -3.8597 | 2.9638 |
| H | -2.4226 | -2.1200 | 3.1581 |
| H | -4.0512 | -1.8071 | 1.2399 |
| H | -4.2422 | -3.5723 | 1.0233 |
| H | -3.2653 | -3.4198 | -1.2769 |
| H | -4.7121 | -2.3818 | -1.1290 |
| H | -2.3322 | -0.3489 | -2.9967 |
| H | -3.7141 | -1.4846 | -3.1446 |
| H | -2.0317 | -2.1222 | -3.0154 |
| H | 0.5831 | 0.9418 | 2.7772 |
| H | 0.4300 | -1.0242 | 2.7794 |

**Stationary point: TS2**

| | | x | y | z |
|---|---|---|---|---|
| C | -1.6947 | 3.3142 | 2.6214 | |
| N | -1.4433 | 3.3143 | 1.1530 | |

| | | | |
|---|---|---|---|
| C | -0.5678 | 4.4634 | 0.7698 |
| C | -0.3331 | 4.4544 | -0.7311 |
| N | 0.2644 | 3.1543 | -1.1628 |
| C | 0.3213 | 3.0858 | -2.6524 |
| Mo | -0.6962 | 1.4174 | 0.0473 |
| S | 1.0874 | 1.5297 | 1.6315 |
| Mo | 1.5880 | -0.1398 | 0.0521 |
| N | 2.6240 | -1.7992 | -1.1820 |
| C | 2.5463 | -1.7692 | -2.6721 |
| Mo | -0.9449 | -1.2636 | 0.0583 |
| N | -2.8475 | -1.3865 | -1.2275 |
| C | -2.7499 | -1.3415 | -2.7159 |
| Cl | -2.6904 | 2.0010 | -1.3459 |
| S | 0.0177 | -0.0062 | -1.7192 |
| S | -1.9774 | 0.1821 | 1.5678 |
| Cl | 3.1131 | 1.3809 | -1.2589 |
| S | 0.8010 | -1.6857 | 1.6350 |
| N | 3.6040 | -0.4480 | 1.1552 |
| C | 4.1655 | -1.7737 | 0.7570 |
| C | 4.0469 | -1.9414 | -0.7480 |
| Cl | -0.3296 | -3.3572 | -1.2111 |
| N | -2.2133 | -2.8578 | 1.1560 |
| C | -3.6320 | -2.7227 | 0.7093 |
| C | -3.6763 | -2.5565 | -0.8004 |
| C | 3.7306 | -0.2386 | 2.6249 |
| C | -2.1343 | -3.0338 | 2.6334 |
| H | -2.3523 | 3.4477 | 0.6829 |
| H | 1.2452 | 3.1160 | -0.8374 |
| H | 4.1673 | 0.2803 | 0.6881 |
| H | 2.1006 | -2.6384 | -0.8824 |
| H | -1.8328 | -3.7134 | 0.7212 |
| H | -3.3287 | -0.5186 | -0.9364 |
| H | -2.4484 | 2.5532 | 2.8635 |
| H | -2.0541 | 4.3053 | 2.9497 |
| H | -0.7609 | 3.0741 | 3.1511 |
| H | 0.3826 | 4.3588 | 1.3213 |
| H | -1.0321 | 5.4194 | 1.0743 |
| H | -1.2901 | 4.5651 | -1.2692 |
| H | 0.3215 | 5.2951 | -1.0230 |
| H | 0.9780 | 2.2567 | -2.9460 |
| H | 0.7230 | 4.0292 | -3.0628 |
| H | -0.6939 | 2.9140 | -3.0385 |
| H | 3.4412 | 0.7913 | 2.8743 |
| H | 4.7710 | -0.4139 | 2.9502 |
| H | 3.0661 | -0.9382 | 3.1537 |
| H | 5.2242 | -1.8523 | 1.0654 |

| | | | |
|---|---|---|---|
| H | 3.5977 | -2.5540 | 1.2926 |
| H | 4.4494 | -2.9216 | -1.0603 |
| H | 4.6228 | -1.1555 | -1.2660 |
| H | 1.5046 | -1.9342 | -2.9776 |
| H | 3.1807 | -2.5637 | -3.1036 |
| H | 2.8878 | -0.7858 | -3.0263 |
| H | -1.0998 | -3.2707 | 2.9167 |
| H | -2.7997 | -3.8524 | 2.9595 |
| H | -2.4359 | -2.0990 | 3.1283 |
| H | -4.0610 | -1.8429 | 1.2189 |
| H | -4.2184 | -3.6109 | 1.0083 |
| H | -3.2552 | -3.4470 | -1.2977 |
| H | -4.7185 | -2.4330 | -1.1444 |
| H | -2.3727 | -0.3561 | -3.0189 |
| H | -3.7441 | -1.5049 | -3.1677 |
| H | -2.0552 | -2.1264 | -3.0490 |
| H | 0.4824 | 0.5210 | 2.7382 |
| H | 0.3906 | -0.5799 | 2.7381 |

## Cluster $1^+$

**Stationary point: Cluster $1^+$**

| | x | y | z |
|---|---|---|---|
| H | 1.1664 | 0.5142 | 0.6820 |
| N | 0.2550 | 0.9712 | 0.8036 |
| C | 0.3554 | 2.4212 | 0.4404 |
| C | -1.0511 | 3.0070 | 0.3782 |
| N | -1.9015 | 2.1640 | -0.5204 |
| H | -2.8927 | 2.3107 | -0.2955 |
| Mo | -1.3990 | -0.0468 | -0.3611 |
| S | 0.0568 | 0.1824 | -2.1669 |
| Mo | -1.9241 | -0.7421 | -2.9879 |
| Cl | -2.6552 | 1.5436 | -3.8191 |
| S | -0.6165 | -2.1061 | 0.4142 |
| Mo | -2.4558 | -2.5340 | -0.9497 |
| S | -3.5537 | -0.4532 | -1.2808 |
| Cl | -2.6079 | 0.3348 | 1.8388 |
| S | -1.2832 | -2.9806 | -2.9181 |
| N | -2.1328 | -4.6852 | -0.3184 |
| H | -1.1658 | -4.9998 | -0.4597 |
| N | -3.7506 | -2.7599 | 0.9044 |
| H | -4.6864 | -2.3787 | 0.7207 |
| Cl | -4.5439 | -3.5846 | -1.9433 |
| N | -0.9963 | -0.7786 | -5.0538 |
| C | -1.8434 | -1.5586 | -6.0116 |

| | | | |
|---|---|---|---|
| C | -3.3025 | -1.1686 | -5.8010 |
| N | -3.6514 | -1.3086 | -4.3520 |
| H | -4.4682 | -0.7251 | -4.1355 |
| H | -0.0374 | -1.1455 | -5.0538 |
| C | -2.5364 | -4.8955 | 1.1088 |
| C | -3.8635 | -4.1843 | 1.3499 |
| H | -2.7242 | -5.2572 | -0.9377 |
| H | -3.3390 | -2.1593 | 1.6365 |
| H | 0.0062 | 0.8754 | 1.7983 |
| H | -1.7938 | 2.4293 | -1.5123 |
| H | -0.9376 | 0.2038 | -5.3575 |
| H | -3.9230 | -2.2757 | -4.1135 |
| H | -1.7436 | -4.4699 | 1.7450 |
| H | -2.6275 | -5.9702 | 1.3403 |
| H | -4.6576 | -4.6508 | 0.7456 |
| H | -4.1508 | -4.2452 | 2.4124 |
| H | 0.9695 | 2.9709 | 1.1735 |
| H | 0.8491 | 2.4845 | -0.5428 |
| H | -1.0226 | 4.0524 | 0.0297 |
| H | -1.5150 | 2.9844 | 1.3770 |
| H | -1.6889 | -2.6282 | -5.7968 |
| H | -1.5406 | -1.3668 | -7.0549 |
| H | -3.4536 | -0.1109 | -6.0692 |
| H | -3.9653 | -1.7861 | -6.4290 |

**Stationary point: TS1**

| | | x | y | z |
|---|---|---|---|---|
| C | 4.8154 | 0.9280 | -0.7087 | |
| C | 4.0076 | 0.0866 | -1.5130 | |
| C | 4.0986 | -1.3080 | -1.2935 | |
| C | 4.9364 | -1.8401 | -0.2973 | |
| C | 5.7082 | -0.9820 | 0.5085 | |
| C | 5.6511 | 0.4047 | 0.2919 | |
| Si | 2.8558 | 0.8842 | -2.7850 | |
| S | 1.6617 | 1.0212 | 0.7467 | |
| Mo | -0.6020 | 1.5949 | 0.5366 | |
| N | 0.0544 | 3.7064 | 0.0042 | |
| Mo | 0.3669 | -0.8364 | 1.2849 | |
| S | 0.5583 | -1.8664 | -0.8295 | |
| Mo | -1.4574 | -0.6372 | -0.7732 | |
| N | -1.7456 | -1.5017 | -2.8535 | |
| N | 2.3713 | -1.7342 | 1.8100 | |
| C | 2.9034 | -1.1536 | 3.0795 | |
| C | 1.7688 | -1.0900 | 4.0984 | |
| N | 0.5924 | -0.3899 | 3.4934 | |

| | | | |
|---|---|---|---|
| Cl | -0.4126 | -3.0566 | 2.2762 |
| S | -1.9073 | -0.1356 | 1.5088 |
| N | -2.5435 | 2.7608 | 0.3679 |
| C | -2.3269 | 4.2333 | 0.2365 |
| C | -1.0742 | 4.4806 | -0.5972 |
| Cl | -0.6334 | 2.6768 | 2.8467 |
| S | -0.4798 | 1.2464 | -1.8010 |
| N | -2.5755 | -2.5740 | -0.4028 |
| C | -3.0704 | -3.1933 | -1.6722 |
| C | -2.0502 | -2.9645 | -2.7841 |
| Cl | -3.8232 | 0.1658 | -1.2809 |
| H | 2.2180 | -2.7462 | 1.9218 |
| H | 0.6655 | 0.6378 | 3.5741 |
| H | 0.3531 | 4.1443 | 0.8871 |
| H | -3.0599 | 2.3696 | -0.4377 |
| H | -2.5471 | -0.9947 | -3.2555 |
| H | -1.9301 | -3.2000 | 0.1037 |
| H | 3.2812 | -0.1447 | 2.8474 |
| H | 3.7413 | -1.7541 | 3.4733 |
| H | 1.4387 | -2.1093 | 4.3561 |
| H | 2.0995 | -0.5861 | 5.0217 |
| H | -0.8429 | 5.5588 | -0.6395 |
| H | -1.2128 | 4.1152 | -1.6282 |
| H | -3.2017 | 4.7294 | -0.2160 |
| H | -2.1864 | 4.6343 | 1.2535 |
| H | -1.1074 | -3.4900 | -2.5613 |
| H | -2.4403 | -3.3385 | -3.7463 |
| H | -4.0200 | -2.6951 | -1.9262 |
| H | -3.2664 | -4.2705 | -1.5415 |
| H | 1.1025 | 0.9200 | -2.0063 |
| C | 3.5863 | 0.9407 | -4.5359 |
| C | 2.6675 | 2.7532 | -2.3811 |
| H | 3.4935 | -2.0047 | -1.8800 |
| H | 4.9839 | -2.9243 | -0.1492 |
| H | 6.3512 | -1.3940 | 1.2925 |
| H | 6.2560 | 1.0816 | 0.9040 |
| H | 4.7812 | 2.0141 | -0.8440 |
| H | 2.8141 | 1.2053 | -5.2797 |
| H | 4.3515 | 1.7349 | -4.5576 |
| H | 4.0629 | -0.0002 | -4.8503 |
| H | 1.7903 | 3.1750 | -2.9004 |
| H | 2.5468 | 2.8793 | -1.2925 |
| H | 3.5640 | 3.3067 | -2.7143 |
| H | 0.8645 | 3.7259 | -0.6259 |
| H | -3.1260 | 2.5668 | 1.1909 |
| H | -0.2648 | -0.6525 | 3.9936 |

| | | | |
|---|---|---|---|
| H | 3.0629 | -1.6180 | 1.0543 |
| H | -3.3659 | -2.4057 | 0.2304 |
| H | -0.9481 | -1.3282 | -3.4768 |
| O | 1.7807 | -0.7421 | -3.2964 |
| H | 1.4289 | -1.1472 | -2.4219 |
| C | 2.1394 | -1.7905 | -4.2624 |
| H | 2.0075 | -1.3716 | -5.2678 |
| H | 3.1784 | -2.1229 | -4.1162 |
| H | 1.4467 | -2.6320 | -4.1106 |

**Stationary point: I1**

| | x | y | z |
|---|---|---|---|
| H | -3.2864 | 0.0935 | -2.4367 |
| N | -3.4462 | 0.2428 | -1.4337 |
| Mo | -1.5106 | 0.2488 | -0.2310 |
| S | -0.5858 | 1.4573 | -2.0303 |
| Mo | 0.9731 | 1.1924 | -0.2786 |
| N | 2.1785 | 2.6305 | -1.5654 |
| H | 2.1764 | 2.3680 | -2.5577 |
| C | -4.4082 | -0.7756 | -0.9075 |
| C | -4.4020 | -0.7121 | 0.6161 |
| N | -2.9944 | -0.8403 | 1.1059 |
| H | -2.9235 | -0.4732 | 2.0620 |
| S | -1.1361 | -1.8540 | -1.4274 |
| Mo | 0.5683 | -1.4752 | 0.2255 |
| N | 1.4816 | -3.4655 | -0.3997 |
| H | 1.4820 | -3.6065 | -1.4165 |
| Cl | -2.6415 | 2.2489 | 0.7634 |
| S | -0.0724 | 0.2981 | 1.6703 |
| N | 2.2810 | -1.7710 | 1.6619 |
| C | 2.8669 | -3.1458 | 1.5912 |
| C | 2.8698 | -3.6093 | 0.1397 |
| Cl | -0.6226 | -3.0352 | 1.8089 |
| S | 2.0522 | -0.6210 | -1.4834 |
| H | 1.9512 | -1.5883 | 2.6174 |
| N | 0.5361 | 3.2069 | 0.6120 |
| C | 1.4288 | 4.2831 | 0.0781 |
| C | 1.6769 | 4.0304 | -1.4041 |
| Cl | 3.0759 | 1.4058 | 1.1162 |
| H | 0.6065 | 3.1711 | 1.6358 |
| H | -3.8448 | 1.1871 | -1.3375 |
| H | -2.6970 | -1.8286 | 1.1725 |
| H | 0.8727 | -4.1945 | -0.0011 |
| H | 2.9875 | -1.0416 | 1.4689 |
| H | 3.1542 | 2.5670 | -1.2419 |

| | | | |
|---|---|---|---|
| H | -0.4595 | 3.3975 | 0.4048 |
| H | -4.0676 | -1.7647 | -1.2549 |
| H | -5.4252 | -0.6031 | -1.2988 |
| H | -4.7783 | 0.2658 | 0.9572 |
| H | -5.0455 | -1.5005 | 1.0407 |
| H | 3.2208 | -4.6524 | 0.0641 |
| H | 3.5336 | -2.9754 | -0.4709 |
| H | 3.8858 | -3.1669 | 2.0117 |
| H | 2.2251 | -3.8056 | 2.1972 |
| H | 0.7369 | 4.1128 | -1.9740 |
| H | 2.3964 | 4.7629 | -1.8076 |
| H | 2.3759 | 4.2267 | 0.6386 |
| H | 0.9858 | 5.2798 | 0.2383 |
| H | -0.5977 | -1.3558 | -2.6178 |
| H | 1.2765 | -0.6741 | -2.6457 |

**Stationary point: TS2**

| | x | y | z |
|---|---|---|---|
| H | 0.2304 | -0.1917 | 0.0489 |
| N | 0.0623 | -0.0473 | 1.0514 |
| Mo | 1.9863 | -0.0150 | 2.2636 |
| S | 2.9139 | 1.2327 | 0.5142 |
| Mo | 4.4931 | 0.9326 | 2.2176 |
| N | 5.7106 | 2.3488 | 0.9242 |
| H | 5.7083 | 2.0815 | -0.0669 |
| C | -0.8801 | -1.0879 | 1.5707 |
| C | -0.8947 | -1.0161 | 3.0938 |
| N | 0.5090 | -1.1085 | 3.6028 |
| H | 0.5603 | -0.7256 | 4.5541 |
| S | 2.4366 | -2.0477 | 1.0686 |
| Mo | 4.0740 | -1.7420 | 2.7302 |
| N | 4.9778 | -3.7304 | 2.0956 |
| H | 4.9633 | -3.8740 | 1.0792 |
| Cl | 0.8304 | 1.9702 | 3.2657 |
| S | 3.4317 | 0.0430 | 4.1605 |
| N | 5.7952 | -2.0515 | 4.1649 |
| C | 6.3925 | -3.4196 | 4.0692 |
| C | 6.3750 | -3.8695 | 2.6131 |
| Cl | 2.9166 | -3.2982 | 4.3524 |
| S | 5.4633 | -0.8940 | 1.0216 |
| H | 5.4689 | -1.8842 | 5.1243 |
| N | 4.0799 | 2.9631 | 3.1050 |
| C | 4.9816 | 4.0253 | 2.5572 |
| C | 5.2268 | 3.7567 | 1.0769 |
| Cl | 6.5821 | 1.1547 | 3.6318 |

| | | | |
|---|---|---|---|
| H | 4.1648 | 2.9279 | 4.1278 |
| H | -0.3573 | 0.8879 | 1.1480 |
| H | 0.8277 | -2.0887 | 3.6836 |
| H | 4.3768 | -4.4600 | 2.5051 |
| H | 6.4908 | -1.3121 | 3.9723 |
| H | 6.6849 | 2.2750 | 1.2498 |
| H | 3.0856 | 3.1686 | 2.9112 |
| H | -0.5105 | -2.0698 | 1.2321 |
| H | -1.8959 | -0.9429 | 1.1657 |
| H | -1.2982 | -0.0455 | 3.4243 |
| H | -1.5247 | -1.8169 | 3.5151 |
| H | 6.7312 | -4.9097 | 2.5230 |
| H | 7.0245 | -3.2257 | 1.9976 |
| H | 7.4187 | -3.4360 | 4.4718 |
| H | 5.7674 | -4.0915 | 4.6794 |
| H | 4.2895 | 3.8495 | 0.5046 |
| H | 5.9570 | 4.4759 | 0.6689 |
| H | 5.9288 | 3.9658 | 3.1169 |
| H | 4.5488 | 5.0280 | 2.7072 |
| H | 3.3228 | -1.4059 | -0.1336 |
| H | 4.3419 | -1.0264 | -0.1471 |

**Optimized with UPBE0-D3**

**Stationary point: Dimethylphenylsilane (1a)**

| | x | y | z |
|---|---|---|---|
| C | 5.8056 | 1.9602 | -2.3372 |
| C | 5.7751 | 0.6021 | -2.6867 |
| C | 6.1397 | -0.3354 | -1.7064 |
| C | 6.5191 | 0.0664 | -0.4281 |
| C | 6.5417 | 1.4224 | -0.1024 |
| C | 6.1841 | 2.3697 | -1.0592 |
| Si | 5.2593 | 0.0453 | -4.4101 |
| H | 5.0144 | 1.2927 | -5.2006 |
| C | 3.6767 | -0.9695 | -4.3281 |
| C | 6.6352 | -0.9556 | -5.2134 |
| H | 6.1294 | -1.3986 | -1.9407 |
| H | 6.7976 | -0.6762 | 0.3149 |
| H | 6.8376 | 1.7384 | 0.8942 |
| H | 6.2007 | 3.4275 | -0.8106 |
| H | 5.5303 | 2.7118 | -3.0742 |
| H | 3.3692 | -1.2948 | -5.3274 |
| H | 3.8262 | -1.8639 | -3.7137 |
| H | 2.8580 | -0.3911 | -3.8889 |
| H | 6.3430 | -1.2878 | -6.2149 |

| | | | |
|---|---|---|---|
| H | 7.5535 | -0.3670 | -5.3019 |
| H | 6.8609 | -1.8460 | -4.6166 |

**Stationary point: MeOH**

| | x | y | z |
|---|---|---|---|
| C | -1.1999 | -0.4927 | 0.0149 |
| H | -0.8302 | -1.5218 | 0.0254 |
| H | -0.8445 | -0.0159 | -0.9098 |
| H | -2.2983 | -0.5292 | -0.0191 |
| O | -0.7111 | 0.1411 | 1.1781 |
| H | -1.0351 | 1.0469 | 1.1716 |

**Stationary point: Methoxydimethylphenylsilane (2a)**

| | x | y | z |
|---|---|---|---|
| C | -8.0752 | -3.4444 | 0.5882 |
| C | -8.0231 | -3.6311 | 1.9683 |
| C | -6.7945 | -3.8389 | 2.5931 |
| C | -5.6242 | -3.8563 | 1.8375 |
| C | -5.6509 | -3.6711 | 0.4458 |
| C | -6.9006 | -3.4670 | -0.1613 |
| Si | -4.0591 | -3.6508 | -0.5577 |
| C | -2.7197 | -4.6493 | 0.2779 |
| O | -3.4619 | -2.0815 | -0.6126 |
| C | -4.2454 | -1.0279 | -1.1377 |
| C | -4.3942 | -4.2722 | -2.2947 |
| H | -6.9639 | -3.3262 | -1.2383 |
| H | -9.0308 | -3.2839 | 0.0963 |
| H | -8.9374 | -3.6168 | 2.5551 |
| H | -6.7493 | -3.9882 | 3.6686 |
| H | -4.6753 | -4.0207 | 2.3434 |
| H | -4.4354 | -1.1580 | -2.2117 |
| H | -5.2138 | -0.9453 | -0.6257 |
| H | -3.7019 | -0.0887 | -0.9984 |
| H | -3.4680 | -4.2776 | -2.8787 |
| H | -4.7841 | -5.2952 | -2.2684 |
| H | -5.1218 | -3.6521 | -2.8283 |
| H | -1.8031 | -4.6279 | -0.3203 |
| H | -2.4814 | -4.2587 | 1.2720 |
| H | -3.0269 | -5.6943 | 0.3880 |

**Stationary point: $H_2$**

| | x | y | z |
|---|---|---|---|
| H | 0.0000 | 0.0000 | -0.0222 |

| | | | |
|---|---|---|---|
| H | 0.0000 | 0.0000 | 0.7222 |

**Stationary point: Cluster $3^{+}$**

| | | x | y | z |
|---|---|---|---|---|
| C | 0.4158 | 1.6883 | 0.3327 | |
| N | 0.8228 | 0.3795 | 0.2873 | |
| C | 0.0110 | -0.2557 | -0.5493 | |
| N | -0.9048 | 0.5944 | -1.0447 | |
| C | -0.6585 | 1.8338 | -0.4981 | |
| Mo | 2.3217 | -0.8508 | 1.2924 | |
| N | 0.9603 | -2.3084 | 0.2310 | |
| C | 0.2012 | -1.6944 | -0.8748 | |
| C | -1.9391 | 0.2729 | -2.0129 | |
| Mo | 4.3885 | -0.7781 | 3.0846 | |
| N | 3.1316 | -0.7852 | 4.9627 | |
| C | 3.3093 | 0.4273 | 5.7841 | |
| C | 4.6849 | 0.9392 | 5.5436 | |
| N | 5.3478 | 0.6495 | 4.4304 | |
| C | 6.5500 | 1.3064 | 4.4912 | |
| C | 6.6003 | 2.0039 | 5.6644 | |
| N | 5.4151 | 1.7580 | 6.3204 | |
| C | 5.0122 | 2.3072 | 7.6038 | |
| Mo | 4.6843 | -2.1026 | 0.7083 | |
| N | 6.0492 | -3.7370 | 1.4648 | |
| C | 7.3785 | -3.7216 | 0.8254 | |
| C | 7.2155 | -3.1599 | -0.5423 | |
| N | 6.1953 | -2.3669 | -0.8473 | |
| C | 6.3573 | -1.9805 | -2.1530 | |
| C | 7.5003 | -2.5541 | -2.6326 | |
| N | 8.0285 | -3.3008 | -1.6036 | |
| C | 9.2649 | -4.0623 | -1.6453 | |
| S | 6.1504 | -0.5820 | 1.6078 | |
| S | 3.1498 | 1.0165 | 2.3658 | |
| S | 3.2073 | -2.7043 | 2.4355 | |
| Cl | 5.7290 | -2.3953 | 4.5135 | |
| S | 3.5097 | -0.6662 | -0.6634 | |
| Cl | 3.6879 | -4.1069 | -0.4934 | |
| Cl | 0.4411 | -1.0253 | 2.9915 | |
| H | 5.6491 | -1.3369 | -2.6510 | |
| H | 6.1389 | -3.6370 | 2.4803 | |
| H | 5.5793 | -4.6220 | 1.2826 | |
| H | 8.0342 | -3.0704 | 1.4147 | |
| H | 7.8290 | -4.7179 | 0.8097 | |
| H | 9.5621 | -4.1853 | -2.6856 | |
| H | 9.1094 | -5.0486 | -1.2051 | |

| | | | |
|---|---|---|---|
| H | 10.0547 | -3.5385 | -1.1025 |
| H | 7.9760 | -2.5073 | -3.5999 |
| H | 2.1498 | -0.8939 | 4.6913 |
| H | 3.3974 | -1.6130 | 5.4927 |
| H | 2.5766 | 1.1739 | 5.4570 |
| H | 3.1263 | 0.2266 | 6.8435 |
| H | 4.6820 | 1.5047 | 8.2661 |
| H | 4.2052 | 3.0309 | 7.4721 |
| H | 5.8701 | 2.8041 | 8.0542 |
| H | 7.2857 | 1.2266 | 3.7062 |
| H | 7.3627 | 2.6373 | 6.0901 |
| H | 1.5300 | -3.0865 | -0.1143 |
| H | 0.3235 | -2.6809 | 0.9328 |
| H | -0.7507 | -2.2063 | -1.0426 |
| H | 0.7945 | -1.7888 | -1.7916 |
| H | -1.4989 | 0.0975 | -2.9967 |
| H | -2.6314 | 1.1112 | -2.0727 |
| H | -2.4879 | -0.6143 | -1.6922 |
| H | 0.9087 | 2.4192 | 0.9545 |
| H | -1.2668 | 2.6909 | -0.7417 |

**Stationary point: TS1A$_{PBE}$**

| | x | y | z |
|---|---|---|---|
| Mo | 0.4257 | 1.3743 | 1.0035 |
| Mo | 1.0909 | -1.2554 | 0.9889 |
| S | -0.5206 | -2.0021 | -0.5195 |
| S | 1.9423 | 0.3617 | -0.4218 |
| S | -1.3003 | 1.2813 | -0.5511 |
| S | -0.0503 | -0.1449 | 2.7329 |
| Cl | 0.7958 | -3.3157 | 2.4562 |
| Cl | 2.2985 | 2.1459 | 2.5700 |
| Mo | -1.5204 | -0.4782 | 0.9339 |
| Cl | -3.2014 | 0.7846 | 2.3828 |
| N | -3.3997 | -1.0370 | -0.0527 |
| C | -3.9364 | -2.1884 | 0.3295 |
| C | 0.1662 | 4.3310 | 0.4335 |
| N | 0.9215 | 3.3009 | 0.0754 |
| C | 3.8044 | -2.4775 | 0.5030 |
| N | 2.5603 | -2.5856 | 0.0559 |
| C | 2.5444 | -3.6103 | -0.8549 |
| H | 1.6370 | -3.9173 | -1.3522 |
| N | 2.9618 | -1.0672 | 2.2398 |
| H | 3.0178 | -0.0964 | 2.5658 |
| H | 2.8452 | -1.6648 | 3.0555 |
| C | 4.1807 | -1.4365 | 1.4976 |

| | | | |
|---|---|---|---|
| H | 4.5436 | -0.5435 | 0.9757 |
| H | 4.9765 | -1.7781 | 2.1659 |
| N | 4.5949 | -3.3911 | -0.0900 |
| C | 6.0208 | -3.5637 | 0.1276 |
| H | 6.3412 | -4.4753 | -0.3745 |
| H | 6.2271 | -3.6589 | 1.1953 |
| H | 6.5747 | -2.7152 | -0.2792 |
| C | 3.8104 | -4.1150 | -0.9590 |
| H | 4.2175 | -4.9164 | -1.5558 |
| N | -0.7285 | 2.9391 | 2.1496 |
| H | -1.6070 | 2.5162 | 2.4660 |
| H | -0.1802 | 3.1780 | 2.9740 |
| C | -0.9962 | 4.1448 | 1.3436 |
| H | -1.9046 | 3.9688 | 0.7557 |
| H | -1.1709 | 5.0243 | 1.9704 |
| N | 0.6250 | 5.4639 | -0.1288 |
| C | 0.0220 | 6.7804 | -0.0313 |
| H | -0.2691 | 6.9825 | 1.0005 |
| H | -0.8565 | 6.8429 | -0.6781 |
| H | 0.7544 | 7.5246 | -0.3414 |
| C | 1.9042 | 3.7853 | -0.7474 |
| H | 2.6611 | 3.1433 | -1.1705 |
| C | 1.7263 | 5.1325 | -0.8862 |
| H | 2.2711 | 5.8760 | -1.4470 |
| N | -2.3303 | -2.2422 | 2.0946 |
| H | -1.5392 | -2.7952 | 2.4355 |
| H | -2.8196 | -1.8639 | 2.9036 |
| C | -3.2378 | -3.0819 | 1.2925 |
| H | -3.9414 | -3.6380 | 1.9190 |
| H | -2.6301 | -3.8113 | 0.7449 |
| N | -5.1197 | -2.3780 | -0.2832 |
| C | -5.9856 | -3.5356 | -0.1419 |
| H | -5.5527 | -4.4009 | -0.6484 |
| H | -6.9522 | -3.3040 | -0.5868 |
| H | -6.1315 | -3.7646 | 0.9151 |
| C | -5.3437 | -1.2896 | -1.0961 |
| C | -4.2710 | -0.4580 | -0.9385 |
| H | -4.0778 | 0.5083 | -1.3777 |
| H | -6.2358 | -1.2054 | -1.6970 |
| H | -0.6518 | 0.7417 | -1.9441 |
| Si | -0.6746 | 0.7127 | -3.8178 |
| C | -2.1356 | -0.4137 | -4.1391 |
| H | -1.7870 | -1.3510 | -4.5801 |
| H | -2.6821 | -0.6512 | -3.2217 |
| H | -2.8313 | 0.0653 | -4.8364 |
| C | 0.4624 | 0.9590 | -5.2961 |

| | | | |
|---|---|---|---|
| H | -0.0347 | 1.6523 | -5.9817 |
| H | 1.4238 | 1.4066 | -5.0237 |
| H | 0.6590 | 0.0243 | -5.8276 |
| C | -1.2953 | 2.4822 | -3.4983 |
| C | -0.4039 | 3.5632 | -3.4351 |
| C | -2.6563 | 2.7401 | -3.2693 |
| C | -0.8538 | 4.8539 | -3.1643 |
| H | 0.6605 | 3.4021 | -3.5920 |
| C | -3.1132 | 4.0301 | -3.0044 |
| H | -3.3777 | 1.9270 | -3.3076 |
| C | -2.2106 | 5.0911 | -2.9477 |
| H | -0.1440 | 5.6758 | -3.1265 |
| H | -4.1735 | 4.2070 | -2.8444 |
| H | -2.5641 | 6.0982 | -2.7437 |
| O | 0.5179 | -0.8654 | -3.2488 |
| H | 0.3102 | -1.1157 | -2.3174 |
| C | 1.9223 | -1.0282 | -3.5170 |
| H | 2.4750 | -0.1283 | -3.2372 |
| H | 2.2920 | -1.8757 | -2.9383 |
| H | 2.0376 | -1.2335 | -4.5799 |

**Stationary point: IA$_{PBE}$**

| | | x | y | z |
|---|---|---|---|---|
| Mo | 0.5884 | 1.2523 | 1.2155 | |
| Mo | 1.2070 | -1.3167 | 0.8859 | |
| S | -0.3673 | -1.8468 | -0.7988 | |
| S | 2.0640 | 0.4146 | -0.3993 | |
| S | -1.1281 | 1.4223 | -0.4497 | |
| S | 0.0531 | -0.4417 | 2.7582 | |
| Cl | 0.8438 | -3.5712 | 2.0494 | |
| Cl | 2.4843 | 1.8112 | 2.8442 | |
| Mo | -1.3770 | -0.5219 | 0.8947 | |
| Cl | -3.0794 | 0.6297 | 2.4148 | |
| N | -3.2422 | -0.9537 | -0.1965 | |
| C | -3.7763 | -2.1469 | 0.0237 | |
| C | 0.3053 | 4.2597 | 0.9660 | |
| N | 1.0776 | 3.2844 | 0.5080 | |
| C | 3.9287 | -2.4964 | 0.3100 | |
| N | 2.6932 | -2.5528 | -0.1683 | |
| C | 2.6915 | -3.4852 | -1.1731 | |
| H | 1.7909 | -3.7388 | -1.7111 | |
| N | 3.0515 | -1.3048 | 2.1830 | |
| H | 3.0971 | -0.3818 | 2.6301 | |
| H | 2.9238 | -2.0041 | 2.9113 | |
| C | 4.2822 | -1.5687 | 1.4178 | |

| | | | |
|---|---|---|---|
| H | 4.6304 | -0.6158 | 1.0025 |
| H | 5.0793 | -1.9708 | 2.0501 |
| N | 4.7285 | -3.3515 | -0.3547 |
| C | 6.1514 | -3.5443 | -0.1373 |
| H | 6.4693 | -4.4330 | -0.6807 |
| H | 6.3502 | -3.6928 | 0.9256 |
| H | 6.7142 | -2.6803 | -0.4976 |
| C | 3.9577 | -3.9835 | -1.3045 |
| H | 4.3741 | -4.7240 | -1.9696 |
| N | -0.5606 | 2.6800 | 2.5350 |
| H | -1.4428 | 2.2222 | 2.7876 |
| H | -0.0148 | 2.8068 | 3.3849 |
| C | -0.8246 | 3.9791 | 1.8928 |
| H | -1.7561 | 3.8952 | 1.3205 |
| H | -0.9600 | 4.7799 | 2.6265 |
| N | 0.6727 | 5.4396 | 0.4314 |
| C | 0.0285 | 6.7220 | 0.6507 |
| H | -0.1509 | 6.8726 | 1.7165 |
| H | -0.9199 | 6.7707 | 0.1104 |
| H | 0.6884 | 7.5107 | 0.2914 |
| C | 1.9787 | 3.8535 | -0.3529 |
| H | 2.7285 | 3.2655 | -0.8592 |
| C | 1.7341 | 5.1968 | -0.4126 |
| H | 2.2124 | 5.9923 | -0.9625 |
| N | -2.2103 | -2.4072 | 1.8059 |
| H | -1.4251 | -2.9979 | 2.0941 |
| H | -2.7200 | -2.1278 | 2.6418 |
| C | -3.0950 | -3.1463 | 0.8897 |
| H | -3.8091 | -3.7782 | 1.4266 |
| H | -2.4696 | -3.7989 | 0.2698 |
| N | -4.9272 | -2.2783 | -0.6629 |
| C | -5.7769 | -3.4551 | -0.7015 |
| H | -5.3086 | -4.2466 | -1.2908 |
| H | -6.7285 | -3.1832 | -1.1564 |
| H | -5.9620 | -3.8151 | 0.3121 |
| C | -5.1304 | -1.1055 | -1.3562 |
| C | -4.0789 | -0.2866 | -1.0532 |
| H | -3.8696 | 0.7196 | -1.3827 |
| H | -5.9947 | -0.9637 | -1.9862 |
| H | -0.4353 | 0.9754 | -1.5499 |
| Si | -0.8131 | -0.2696 | -4.5485 |
| C | -2.2405 | -1.4056 | -4.2040 |
| H | -1.9467 | -2.4439 | -4.3852 |
| H | -2.5807 | -1.3129 | -3.1678 |
| H | -3.0839 | -1.1711 | -4.8617 |
| C | -0.0169 | -0.4714 | -6.2147 |

| | | | |
|---|---|---|---|
| H | -0.7434 | -0.1788 | -6.9802 |
| H | 0.8650 | 0.1643 | -6.3350 |
| H | 0.2681 | -1.5112 | -6.4005 |
| C | -1.1546 | 1.4955 | -4.0605 |
| C | -0.1043 | 2.4072 | -3.8609 |
| C | -2.4679 | 1.9322 | -3.8311 |
| C | -0.3571 | 3.7060 | -3.4296 |
| H | 0.9272 | 2.1016 | -4.0210 |
| C | -2.7256 | 3.2364 | -3.4125 |
| H | -3.3019 | 1.2487 | -3.9716 |
| C | -1.6698 | 4.1213 | -3.2046 |
| H | 0.4683 | 4.3905 | -3.2578 |
| H | -3.7489 | 3.5581 | -3.2414 |
| H | -1.8676 | 5.1348 | -2.8667 |
| O | 0.4382 | -0.8093 | -3.4034 |
| H | 0.1553 | -1.0873 | -2.4114 |
| C | 1.8425 | -1.0182 | -3.6839 |
| H | 2.2856 | -0.0799 | -4.0168 |
| H | 2.3055 | -1.3324 | -2.7498 |
| H | 1.9445 | -1.7921 | -4.4446 |

**Stationary point: TS1B$_{PBE}$**

| | x | y | z |
|---|---|---|---|
| Mo | 0.6522 | 1.3208 | 1.2229 |
| Mo | 1.2702 | -1.2382 | 0.8996 |
| S | -0.3068 | -1.7905 | -0.7991 |
| S | 2.1326 | 0.4855 | -0.3852 |
| S | -1.0690 | 1.5020 | -0.4442 |
| S | 0.0968 | -0.3716 | 2.7622 |
| Cl | 0.8830 | -3.4973 | 2.0343 |
| Cl | 2.5253 | 1.8806 | 2.8608 |
| Mo | -1.3231 | -0.4463 | 0.8925 |
| Cl | -3.0221 | 0.7095 | 2.4050 |
| N | -3.1901 | -0.8643 | -0.1972 |
| C | -3.7306 | -2.0561 | 0.0162 |
| C | 0.3728 | 4.3276 | 0.9628 |
| N | 1.1461 | 3.3495 | 0.5118 |
| C | 3.9979 | -2.4139 | 0.3512 |
| N | 2.7680 | -2.4700 | -0.1412 |
| C | 2.7793 | -3.3960 | -1.1517 |
| H | 1.8856 | -3.6474 | -1.7019 |
| N | 3.0932 | -1.2342 | 2.2177 |
| H | 3.1342 | -0.3151 | 2.6732 |
| H | 2.9566 | -1.9389 | 2.9393 |
| C | 4.3356 | -1.4917 | 1.4681 |

| | | | |
|---|---|---|---|
| H | 4.6883 | -0.5359 | 1.0636 |
| H | 5.1235 | -1.8961 | 2.1103 |
| N | 4.8067 | -3.2632 | -0.3094 |
| C | 6.2277 | -3.4536 | -0.0780 |
| H | 6.5530 | -4.3400 | -0.6208 |
| H | 6.4159 | -3.6048 | 0.9864 |
| H | 6.7922 | -2.5872 | -0.4299 |
| C | 4.0478 | -3.8909 | -1.2716 |
| H | 4.4732 | -4.6264 | -1.9365 |
| N | -0.5106 | 2.7518 | 2.5280 |
| H | -1.3976 | 2.2972 | 2.7709 |
| H | 0.0253 | 2.8747 | 3.3847 |
| C | -0.7616 | 4.0539 | 1.8858 |
| H | -1.6922 | 3.9790 | 1.3109 |
| H | -0.8928 | 4.8541 | 2.6209 |
| N | 0.7415 | 5.5040 | 0.4222 |
| C | 0.0961 | 6.7875 | 0.6317 |
| H | -0.0903 | 6.9426 | 1.6956 |
| H | -0.8487 | 6.8337 | 0.0848 |
| H | 0.7585 | 7.5745 | 0.2734 |
| C | 2.0491 | 3.9136 | -0.3507 |
| H | 2.8002 | 3.3231 | -0.8522 |
| C | 1.8045 | 5.2564 | -0.4183 |
| H | 2.2841 | 6.0488 | -0.9717 |
| N | -2.1619 | -2.3330 | 1.7911 |
| H | -1.3797 | -2.9289 | 2.0775 |
| H | -2.6711 | -2.0561 | 2.6283 |
| C | -3.0505 | -3.0647 | 0.8719 |
| H | -3.7654 | -3.6971 | 1.4070 |
| H | -2.4290 | -3.7159 | 0.2468 |
| N | -4.8914 | -2.1702 | -0.6561 |
| C | -5.7505 | -3.3402 | -0.6975 |
| H | -5.2953 | -4.1281 | -1.3018 |
| H | -6.7054 | -3.0556 | -1.1372 |
| H | -5.9256 | -3.7106 | 0.3140 |
| C | -5.0955 | -0.9871 | -1.3313 |
| C | -4.0342 | -0.1795 | -1.0328 |
| H | -3.8220 | 0.8289 | -1.3530 |
| H | -5.9665 | -0.8316 | -1.9488 |
| H | -0.3684 | 1.0783 | -1.5442 |
| Si | -0.8294 | -0.2008 | -4.4817 |
| C | -2.2683 | -1.3288 | -4.1461 |
| H | -1.9798 | -2.3674 | -4.3358 |
| H | -2.6068 | -1.2446 | -3.1087 |
| H | -3.1124 | -1.0860 | -4.7998 |
| C | -0.0662 | -0.3988 | -6.1674 |

| | | | |
|---|---|---|---|
| H | -0.8034 | -0.1011 | -6.9206 |
| H | 0.8167 | 0.2334 | -6.2989 |
| H | 0.2138 | -1.4386 | -6.3618 |
| C | -1.1853 | 1.5736 | -4.0227 |
| C | -0.1364 | 2.4897 | -3.8362 |
| C | -2.4977 | 2.0127 | -3.7942 |
| C | -0.3885 | 3.7933 | -3.4184 |
| H | 0.8950 | 2.1825 | -3.9953 |
| C | -2.7558 | 3.3210 | -3.3876 |
| H | -3.3314 | 1.3269 | -3.9254 |
| C | -1.7007 | 4.2097 | -3.1925 |
| H | 0.4370 | 4.4807 | -3.2581 |
| H | -3.7791 | 3.6433 | -3.2165 |
| H | -1.8988 | 5.2266 | -2.8651 |
| O | 0.4020 | -0.7166 | -3.3407 |
| H | 0.1092 | -1.0591 | -2.2548 |
| C | 1.7910 | -0.9332 | -3.6515 |
| H | 2.2469 | 0.0018 | -3.9806 |
| H | 2.2738 | -1.2647 | -2.7325 |
| H | 1.8834 | -1.6985 | -4.4238 |

**Stationary point: I1**

| | x | y | z |
|---|---|---|---|
| Mo | 0.3930 | 1.4831 | 0.1632 |
| Mo | 1.1092 | -1.0478 | 0.2590 |
| S | -0.4595 | -1.9574 | -1.3318 |
| S | 1.8607 | 0.4730 | -1.3220 |
| S | -1.4039 | 1.3011 | -1.4588 |
| S | -0.1085 | 0.0423 | 1.9635 |
| Cl | 0.7995 | -3.1386 | 1.6480 |
| Cl | 2.1718 | 2.3952 | 1.6668 |
| Mo | -1.5343 | -0.4344 | 0.1615 |
| Cl | -3.2763 | 0.8865 | 1.4360 |
| N | -3.3617 | -1.1594 | -0.8161 |
| C | -3.8468 | -2.3110 | -0.3704 |
| C | -0.0015 | 4.4026 | -0.5330 |
| N | 0.7873 | 3.3857 | -0.8588 |
| C | 3.8980 | -2.1324 | -0.1175 |
| N | 2.6779 | -2.3193 | -0.6027 |
| C | 2.7570 | -3.3363 | -1.5182 |
| H | 1.8896 | -3.6937 | -2.0510 |
| N | 2.9029 | -0.7578 | 1.5620 |
| H | 2.9024 | 0.2144 | 1.8891 |
| H | 2.7867 | -1.3587 | 2.3758 |
| C | 4.1733 | -1.0613 | 0.8759 |

| | | | |
|---|---|---|---|
| H | 4.5034 | -0.1532 | 0.3582 |
| H | 4.9566 | -1.3463 | 1.5842 |
| N | 4.7639 | -2.9900 | -0.6860 |
| C | 6.1921 | -3.0640 | -0.4320 |
| H | 6.5804 | -3.9710 | -0.8930 |
| H | 6.3784 | -3.1078 | 0.6425 |
| H | 6.7011 | -2.1964 | -0.8576 |
| C | 4.0551 | -3.7584 | -1.5820 |
| H | 4.5322 | -4.5284 | -2.1680 |
| N | -0.8890 | 3.0288 | 1.2097 |
| H | -1.7697 | 2.5839 | 1.4941 |
| H | -0.3869 | 3.2850 | 2.0574 |
| C | -1.1527 | 4.2267 | 0.3921 |
| H | -2.0681 | 4.0547 | -0.1859 |
| H | -1.3180 | 5.1123 | 1.0127 |
| N | 0.3801 | 5.5164 | -1.1829 |
| C | -0.2614 | 6.8176 | -1.1108 |
| H | -0.4502 | 7.0824 | -0.0691 |
| H | -1.2030 | 6.8113 | -1.6641 |
| H | 0.4070 | 7.5597 | -1.5452 |
| C | 1.7130 | 3.8618 | -1.7513 |
| H | 2.4843 | 3.2300 | -2.1633 |
| C | 1.4651 | 5.1879 | -1.9647 |
| H | 1.9572 | 5.9203 | -2.5855 |
| N | -2.2756 | -2.1395 | 1.4131 |
| H | -1.4650 | -2.6325 | 1.8001 |
| H | -2.7905 | -1.7250 | 2.1885 |
| C | -3.1365 | -3.0853 | 0.6807 |
| H | -3.8300 | -3.6051 | 1.3484 |
| H | -2.4963 | -3.8431 | 0.2142 |
| N | -4.9725 | -2.6315 | -1.0320 |
| C | -5.7633 | -3.8367 | -0.8530 |
| H | -5.2670 | -4.6917 | -1.3172 |
| H | -6.7367 | -3.6874 | -1.3183 |
| H | -5.9086 | -4.0311 | 0.2107 |
| C | -5.2113 | -1.6323 | -1.9493 |
| C | -4.2059 | -0.7195 | -1.8036 |
| H | -4.0415 | 0.2117 | -2.3229 |
| H | -6.0678 | -1.6590 | -2.6048 |
| H | -0.1752 | -1.1981 | -2.4455 |
| H | -0.7059 | 0.7658 | -2.5180 |

**Stationary point: TS2**

| | x | y | z |
|---|---|---|---|
| Mo | 0.4749 | 1.4634 | -0.8195 |

| | | | |
|---|---|---|---|
| Mo | 1.2101 | -1.0902 | -0.7145 |
| S | -0.3544 | -1.8893 | -2.2888 |
| S | 1.9852 | 0.4509 | -2.2412 |
| S | -1.2380 | 1.1850 | -2.4232 |
| S | -0.0048 | 0.0156 | 0.9798 |
| Cl | 0.9236 | -3.1570 | 0.7166 |
| Cl | 2.2489 | 2.4008 | 0.6889 |
| Mo | -1.4411 | -0.4696 | -0.8153 |
| Cl | -3.1931 | 0.8027 | 0.5091 |
| N | -3.2572 | -1.1807 | -1.8114 |
| C | -3.7496 | -2.3349 | -1.3796 |
| C | 0.0386 | 4.3655 | -1.5366 |
| N | 0.8437 | 3.3590 | -1.8549 |
| C | 3.9832 | -2.2063 | -1.1058 |
| N | 2.7580 | -2.3693 | -1.5880 |
| C | 2.8155 | -3.3818 | -2.5101 |
| H | 1.9403 | -3.7196 | -3.0429 |
| N | 3.0229 | -0.8074 | 0.5774 |
| H | 3.0382 | 0.1685 | 0.8906 |
| H | 2.8998 | -1.3932 | 1.4011 |
| C | 4.2847 | -1.1446 | -0.1090 |
| H | 4.6426 | -0.2456 | -0.6240 |
| H | 5.0591 | -1.4529 | 0.5993 |
| N | 4.8309 | -3.0760 | -1.6828 |
| C | 6.2590 | -3.1760 | -1.4355 |
| H | 6.6299 | -4.0855 | -1.9055 |
| H | 6.4489 | -3.2317 | -0.3622 |
| H | 6.7805 | -2.3136 | -1.8564 |
| C | 4.1055 | -3.8266 | -2.5805 |
| H | 4.5668 | -4.6015 | -3.1728 |
| N | -0.8083 | 3.0005 | 0.2323 |
| H | -1.6778 | 2.5464 | 0.5336 |
| H | -0.3002 | 3.2788 | 1.0696 |
| C | -1.1033 | 4.1796 | -0.6017 |
| H | -2.0183 | 3.9770 | -1.1709 |
| H | -1.2847 | 5.0708 | 0.0061 |
| N | 0.3947 | 5.4767 | -2.2045 |
| C | -0.2685 | 6.7675 | -2.1401 |
| H | -0.4147 | 7.0603 | -1.0990 |
| H | -1.2337 | 6.7270 | -2.6494 |
| H | 0.3645 | 7.5080 | -2.6270 |
| C | 1.7532 | 3.8391 | -2.7617 |
| H | 2.5323 | 3.2165 | -3.1732 |
| C | 1.4794 | 5.1576 | -2.9902 |
| H | 1.9534 | 5.8903 | -3.6246 |
| N | -2.1904 | -2.1948 | 0.4196 |

| | | | |
|---|---|---|---|
| H | -1.3789 | -2.6906 | 0.8005 |
| H | -2.7069 | -1.7932 | 1.2006 |
| C | -3.0470 | -3.1264 | -0.3353 |
| H | -3.7459 | -3.6568 | 0.3181 |
| H | -2.4028 | -3.8759 | -0.8099 |
| N | -4.8767 | -2.6394 | -2.0462 |
| C | -5.6760 | -3.8413 | -1.8828 |
| H | -5.1901 | -4.6917 | -2.3659 |
| H | -6.6517 | -3.6758 | -2.3376 |
| H | -5.8146 | -4.0533 | -0.8216 |
| C | -5.1079 | -1.6280 | -2.9518 |
| C | -4.0968 | -0.7234 | -2.7945 |
| H | -3.9250 | 0.2124 | -3.3032 |
| H | -5.9634 | -1.6416 | -3.6089 |
| H | -0.0976 | -0.7282 | -3.4339 |
| H | -0.3731 | 0.2530 | -3.4760 |

## 10. References


(1) Gutiérrez-Blanco, M.; Guillamón, E.; Safont, V. S.; Algarra, A. G.; Fernández-Trujillo, M. J.; Junge, K.; Basallote, M. G.; Llusar, R.; Beller, M. Efficient (Z)-Selective Semihydrogenation of Alkynes Catalyzed by Air-Stable Imidazolyl Amino Molybdenum Cluster Sulfides. *Inorg. Chem. Front.* **2023**, *10* (6), 1786–1794. https://doi.org/10.1039/d2qi02755k.

(2) Corporation, W. MassLynx 4.1: Getting Started Guide. *ReVision* **2005**, 94.

(3) Man on the Moon series X104 kit https://manonthemoontech.com/.

(4) Ito, S.; White, F. J.; Okunishi, E.; Aoyama, Y.; Yamano, A.; Sato, H.; Ferrara, J. D.; Jasnowski, M.; Meyer, M. Structure Determination of Small Molecule Compounds by an Electron Diffractometer for 3D ED/MicroED. *CrystEngComm* **2021**, *23* (48), 8622–8630. https://doi.org/10.1039/D1CE01172C.

(5) *No Title*; Rigaku Oxford Diffraction, CrysAlisPro software system, Rigaku Corporation, Wrocław, Poland (version 1.171.43.53a), 2023.

(6) Dolomanov, O. V.; Bourhis, L. J.; Gildea, R. J.; Howard, J. A. K.; Puschmann, H. OLEX2 : A Complete Structure Solution, Refinement and Analysis Program. *J. Appl. Crystallogr.* **2009**, *42* (2), 339–341. https://doi.org/10.1107/S0021889808042726.

(7) *No Title*; Rigaku Oxford Diffraction, AutoChem 6 software system in conjunction with OLEX2, Rigaku Corporation, Wrocław, Poland (version 1.5-ac6-009), 2023.

(8) Sheldrick, G. M. SHELXT – Integrated Space-Group and Crystal-Structure Determination. *Acta Crystallogr. Sect. A Found. Adv.* **2015**, *C71* (1), 3–8. https://doi.org/10.1107/S2053273314026370.

(9) Sheldrick, G. M. Crystal Structure Refinement with SHELXL. *Acta Crystallogr. Sect. C Struct. Chem.* **2015**, *A71* (1), 3–8. https://doi.org/10.1107/S2053229614024218.

(10) Hirshfeld, F. L. Can X-Ray Data Distinguish Bonding Effects from Vibrational Smearing? *Acta Crystallogr. Sect. A* **1976**, *32* (2), 239–244. https://doi.org/10.1107/S0567739476000533.

(11) Thorn, A.; Dittrich, B.; Sheldrick, G. M. Enhanced Rigid-Bond Restraints. *Acta Crystallogr. Sect. A Found. Crystallogr.* **2012**, *68* (4), 448–451. https://doi.org/10.1107/S0108767312014535.

(12) Garcia-Borràs, M.; Kan, S. B. J.; Lewis, R. D.; Tang, A.; Jimenez-Osés, G.; Arnold, F. H.; Houk, K. N. Origin and Control of Chemoselectivity in Cytochrome c Catalyzed Carbene Transfer into Si-H and N-H Bonds. *J. Am. Chem. Soc.* **2021**, *143* (18), 7114–7123. https://doi.org/10.1021/jacs.1c02146.

(13) Frisch, M. J.; Trucks, G. W.; Schlegel, H. B.; Scuseria, G. E.; Robb, M. A.; Cheeseman, J. R.; Scalmani, G.; Barone, V.; Petersson, G. A.; Nakatsuji, H.; Li, X.; Caricato, M.; Marenich, A. V.; Bloino, J.; Janesko, B. G.; Gomperts, R.; Mennucci, B.; Hratchian, H. P.; Ortiz, J. V.; Izmaylov, A. F.; Sonnenberg, J. L.; Williams-Young, D.; Ding, F.; Lipparini, F.; Egidi, F.; Goings, J.; Peng, B.; Petrone, A.; Henderson, T.; Ranasinghe, D.; Zakrzewski, V. G.; Gao, J.; Rega, N.; Zheng, G.; Liang, W.; Hada, M.; Ehara, M.; Toyota, K.; Fukuda, R.; Hasegawa, J.; Ishida, M.; Nakajima, T.; Honda, Y.; Kitao, O.; Nakai, H.; Vreven, T.; Throssell, K.; Montgomery Jr., J. A.; Peralta, J. E.; Ogliaro, F.; Bearpark, M. J.; Heyd, J. J.; Brothers, E. N.; Kudin, K. N.; Staroverov, V. N.; Keith, T. a.; Kobayashi, R.; Normand, J.; Raghavachari, K.; Rendell, A. P.; Burant, J. C.; Iyengar, S. S.; Tomasi, J.; Cossi, M.; Millam, J. M.; Klene, M.; Adamo, C.; Cammi, R.; Ochterski, J. W.; Martin, R. L.; Morokuma, K.; Farkas, O.; Foresman, J. B.; Fox, D. J. G16_C.02. *Gaussian 16* Revision C.02. Gaussian, Inc., Wallingford CT, 201.

(14) Perdew, J. P. Density-Functional Approximation for the Correlation Energy of the Inhomogeneous Electron Gas. *Phys. Rev. B* **1986**, *33* (12), 8822–8824. https://doi.org/10.1103/PhysRevB.33.8822.

(15) Becke, A. D. Density-Functional Exchange-Energy Approximation with Correct Asymptotic Behavior. *Phys. Rev. A* **1988**, *38* (6), 3098–3100. https://doi.org/10.1103/PhysRevA.38.3098.

(16) Adamo, C.; Barone, V. Toward Reliable Density Functional Methods without Adjustable Parameters: The PBE0 Model. *J. Chem. Phys.* **1999**, *110* (13), 6158–6170. https://doi.org/10.1063/1.478522.

(17) Andrae, D.; Häubermann, U.; Dolg, M.; Stoll, H.; Preub, H. Energy-Adjustedab Initio Pseudopotentials for the Second and Third Row Transition Elements. *Theor. Chim. Acta* **1990**, *77* (2), 123–141. https://doi.org/10.1007/BF01114537.

(18) Höllwarth, A.; Böhme, M.; Dapprich, S.; Ehlers, A. W.; Gobbi, A.; Jonas, V.; Köhler, K. F.; Stegmann, R.; Veldkamp, A.; Frenking, G. A Set of D-Polarization Functions for Pseudo-Potential Basis Sets of the Main Group Elements Al□Bi and f-Type Polarization Functions for Zn, Cd, Hg. *Chem. Phys. Lett.* **1993**, *208* (3–4), 237–240. https://doi.org/10.1016/0009-2614(93)89068-S.

(19) Hehre, W. J.; Ditchfield, R.; Pople, J. A. Self—Consistent Molecular Orbital Methods. XII. Further Extensions of Gaussian—Type Basis Sets for Use in Molecular Orbital Studies of Organic Molecules. *J. Chem. Phys.* **1972**, *56* (5), 2257–2261. https://doi.org/10.1063/1.1677527.

(20) Hariharan, P. C.; Pople, J. A. The Influence of Polarization Functions on Molecular Orbital Hydrogenation Energies. *Theor. Chim. Acta* **1973**, *28* (3), 213–222. https://doi.org/10.1007/BF00533485.

(21) Tomasi, J.; Mennucci, B.; Cammi, R. Quantum Mechanical Continuum Solvation Models. *Chem. Rev.* **2005**, *105* (8), 2999–3094. https://doi.org/10.1021/cr9904009.

(22) Cossi, M.; Scalmani, G.; Rega, N.; Barone, V. New Developments in the Polarizable Continuum Model for Quantum Mechanical and Classical Calculations on Molecules in Solution. *J. Chem. Phys.* **2002**, *117* (1), 43–54. https://doi.org/10.1063/1.1480445.

(23) Lu, T.; Chen, F. Multiwfn: A Multifunctional Wavefunction Analyzer. *J. Comput. Chem.* **2012**, *33* (5), 580–592. https://doi.org/10.1002/jcc.22885.

(24) Humphrey, W.; Dalke, A.; Schulten, K. VMD: Visual Molecular Dynamics. *J. Mol. Graph.* **1996**, *14* (1), 33–38. https://doi.org/10.1016/0263-7855(96)00018-5.

(25) Momma, K.; Izumi, F. VESTA 3 for Three-Dimensional Visualization of Crystal, Volumetric and Morphology Data. *J. Appl. Crystallogr.* **2011**, *44* (6), 1272–1276. https://doi.org/10.1107/S0021889811038970.

(26) Contreras-García, J.; Johnson, E. R.; Keinan, S.; Chaudret, R.; Piquemal, J. P.; Beratan, D. N.; Yang, W. NCIPLOT: A Program for Plotting Noncovalent Interaction Regions. *J. Chem. Theory Comput.* **2011**, *7* (3), 625–632. https://doi.org/10.1021/ct100641a.

(27) Logallo, A.; Mu, M.; García-Melchor, M.; Hevia, E. Building Square Planar Cobalt (II) Complexes via Sodium Mediated Cobaltation of Fluoroarenes. *Angew. Chemie Int. Ed.* **2022**, *61* (49), e202213246. https://doi.org/10.1002/anie.202213246.
